# Discovery of Novel Quinoline-Based Proteasome Inhibitors for Human African Trypanosomiasis (HAT)

**DOI:** 10.1021/acs.jmedchem.2c00791

**Published:** 2022-08-22

**Authors:** Dennis C. Koester, Vanessa M. Marx, Sarah Williams, Jan Jiricek, Maxime Dauphinais, Olivier René, Sarah L. Miller, Lei Zhang, Debjani Patra, Yen-Liang Chen, Harry Cheung, Jonathan Gable, Suresh B. Lakshminarayana, Colin Osborne, Jean-Rene Galarneau, Upendra Kulkarni, Wendy Richmond, Angela Bretz, Linda Xiao, Frantisek Supek, Christian Wiesmann, Srinivas Honnappa, Celine Be, Pascal Mäser, Marcel Kaiser, Ryan Ritchie, Michael P. Barrett, Thierry T. Diagana, Christopher Sarko, Srinivasa P. S. Rao

**Affiliations:** † Global Discovery Chemistry, Novartis Institutes for Biomedical Research, Emeryville, California 94608, United States; ‡ Novartis Institutes for Tropical Diseases, Emeryville, California 94608, United States; § Lead Discovery, Novartis Institutes for Tropical Diseases, Emeryville, California 94608, United States; ∥ Pharmacokinetic Sciences, Novartis Institutes for Tropical Diseases, Emeryville, California 94608, United States; ⊥ Pharmacokinetic Sciences, Pharmacology and Comparative Medicine, Novartis Institutes for Tropical Diseases, Emeryville, California 94608, United States; # Preclinical Safety, Novartis Institutes for Biomedical Research, Cambridge, Massachusetts 02139, United States; ∇ Chemical and Pharmaceutical Profiling, Novartis Institutes for Biomedical Research, Cambridge, Massachusetts 02139, United States; ○ Global Discovery Chemistry, Novartis Institutes for Biomedical Research, San Diego, California 92121, United States; ◆ Pharmacology, Novartis Institutes for Tropical Diseases, Emeryville, California 94608, United States; ¶ Novartis Institutes for Biomedical Research, San Diego, California 92121, United States; ⋈ Novartis Institutes for Biomedical Research, 4056 Basel, Switzerland; ⧓ Swiss Tropical and Public Health Institute, Kreuzstrasse 2, 4123 Allschwil, Switzerland; ⧖ University of Basel, CH 4000 Basel, Switzerland; ● University of Glasgow, University Place, Glasgow G12 8TA, U.K.

## Abstract

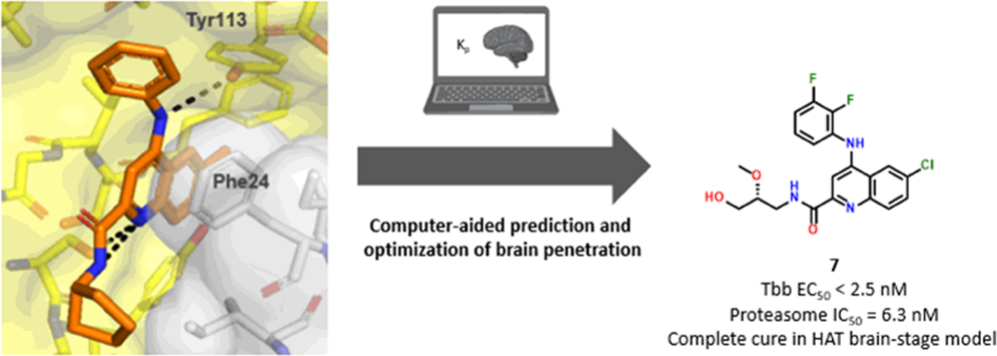

Human African Trypanosomiasis (HAT) is a vector-borne disease caused by kinetoplastid parasites of the *Trypanosoma* genus. The disease proceeds in two stages, with a hemolymphatic blood stage and a meningo-encephalic brain stage. In the latter stage, the parasite causes irreversible damage to the brain leading to sleep cycle disruption and is fatal if untreated. An orally bioavailable treatment is highly desirable. In this study, we present a brain-penetrant, parasite-selective 20S proteasome inhibitor that was rapidly optimized from an HTS singleton hit to drug candidate compound **7** that showed cure in a stage II mouse efficacy model. Here, we describe hit expansion and lead optimization campaign guided by cryo-electron microscopy and an *in silico* model to predict the brain-to-plasma partition coefficient *K*_p_ as an important parameter to prioritize compounds for synthesis. The model combined with in vitro and in vivo experiments allowed us to advance compounds with favorable unbound brain-to-plasma ratios (*K*_p,uu_) to cure a CNS disease such as HAT.

## Introduction

Human African Trypanosomiasis (HAT), also known as African sleeping sickness, is a devastating neglected tropical disease caused by protozoa parasites of the *Trypanosoma brucei* (Tb) genus and transmitted by the Tsetse fly (*Glossina* genus).^1−4^ The disease proceeds in two stages. In the first stage, the parasites multiply in the subcutaneous tissue, the blood, and the lymphatic system.^3−5^ In the second stage, the parasites cross the blood–brain barrier (BBB) to invade the central nervous system (CNS).^6,7^ At this stage, patients typically display disturbances of the sleep cycle, which gives the disease its name. African sleeping sickness is fatal if untreated. Gambiense HAT infections can often linger for months or even years without symptoms and become chronic.^3^ Patients are often in an advanced CNS stage of the disease when symptoms emerge. Two subspecies of the parasite are observed in the field with *T. bruceigambiense* accounting for 97% of the reported cases and *T. bruceirhodiense* for the remaining 3%.^1−3^ The most recent epidemic started in 1970 and lasted until the late 1990s.^1,3^ In 2019, the WHO published new guidelines for the treatment of sleeping sickness with the approval of 10-day oral dosing of fexinidazole.^8,9^ Prior to the approval of fexinidazole, the standard of care as of 2009 was oral nifurtimox combined with an IV infusion of eflornithine.^10^

Tropical disease drug discovery is challenging and very few parasite drug targets have been validated, yet some breakthroughs have emerged for the treatment of sleeping sickness.^11−13^ A few interesting approaches have been reported elsewhere.^14−16^ The 20S proteasome core consists of four stacked rings arranged in αββα-configuration. Each ring consists of seven different subunits with β1, β2, and β5 being the catalytically active subunits that cleave cellular proteins selected for degradation.^17,18^ The proteasome was previously demonstrated to be a validated target for combatting parasitic diseases including HAT, Chagas disease, and Leishmaniasis.^19−21^ The compounds discussed here are *T. b. brucei*-specific chymotrypsin-like proteasome inhibitors, which are binding at the interface of the β4 and the β5 subunit with more than 1000× selectivity against the human proteasome. For all compounds presented in this paper which we profiled in a biochemical assay on the human proteasome, we measured activities of >10 μM (details in the Supporting Information).

## Results and Discussion

Our starting point was compound **1**, which was discovered as a singleton hit from a biochemical HTS screen with 3 million compounds against *Leishmania tarentolae* 20S proteasome. This compound showed promising inhibition of the *T. b. brucei* chymotrypsin activity. Compound **1** not only had <1 μM cellular potency against *T. b. brucei* but also an attractive *in vitro* ADME profile, particularly the high passive permeability and low P-gp efflux as assessed by MDCK-MDR1, which struck us as an ideal starting point for a compound requiring brain penetration. After rapidly screening different tertiary and secondary amines, we noted a steep increase in activity with the latter (*e.g.*, compound **2**) (Table 1).

**Table 1:**
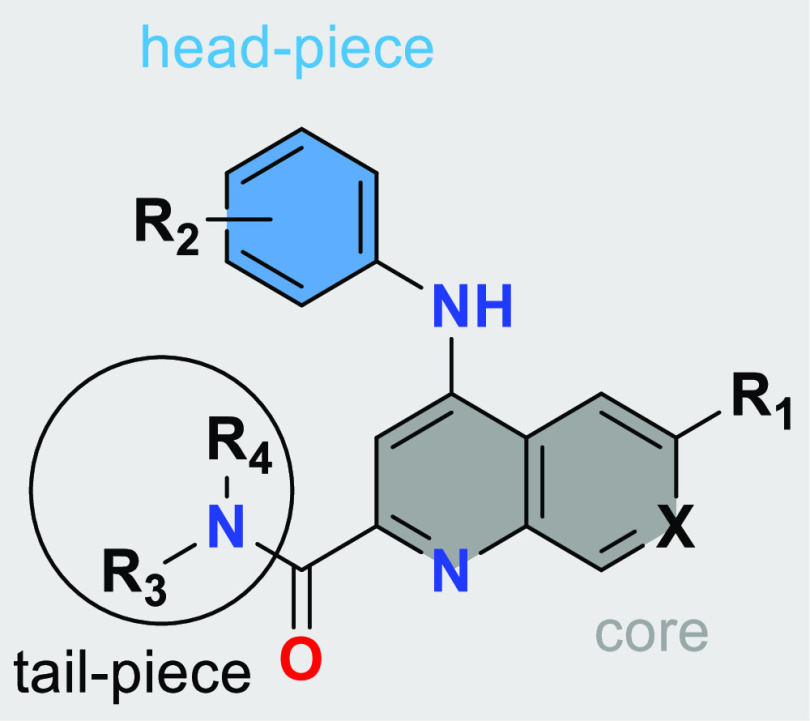

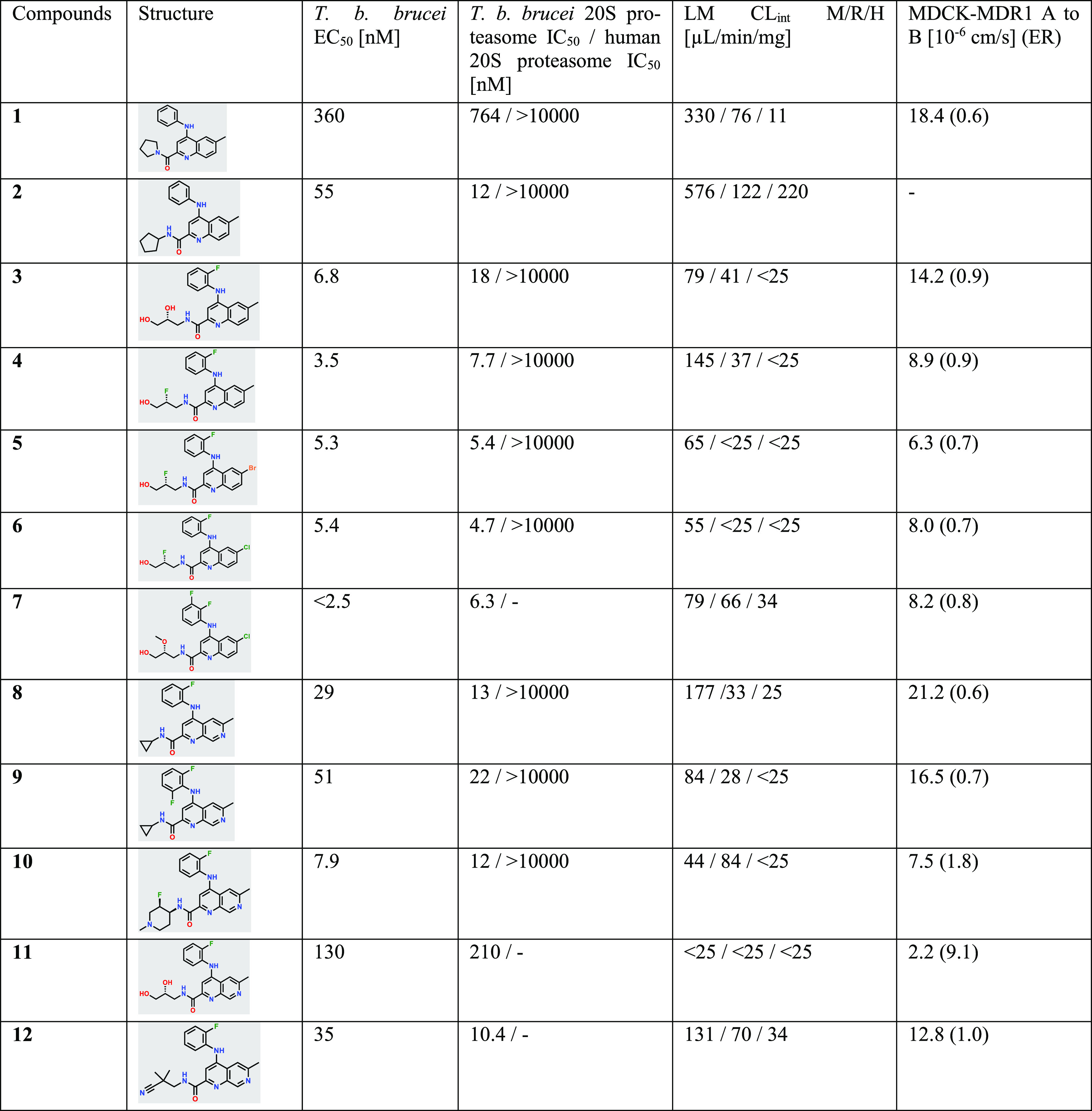
SAR (Biochemical and Cellular) and ADME Properties in Quinoline and Naphthyridine Series^a^

a Details on the biochemical and cellular assay as well as on clearance (Cl_int_) and permeability (MDCK-MDR1) can be found in the Experimental Section.

**Figure 1:**
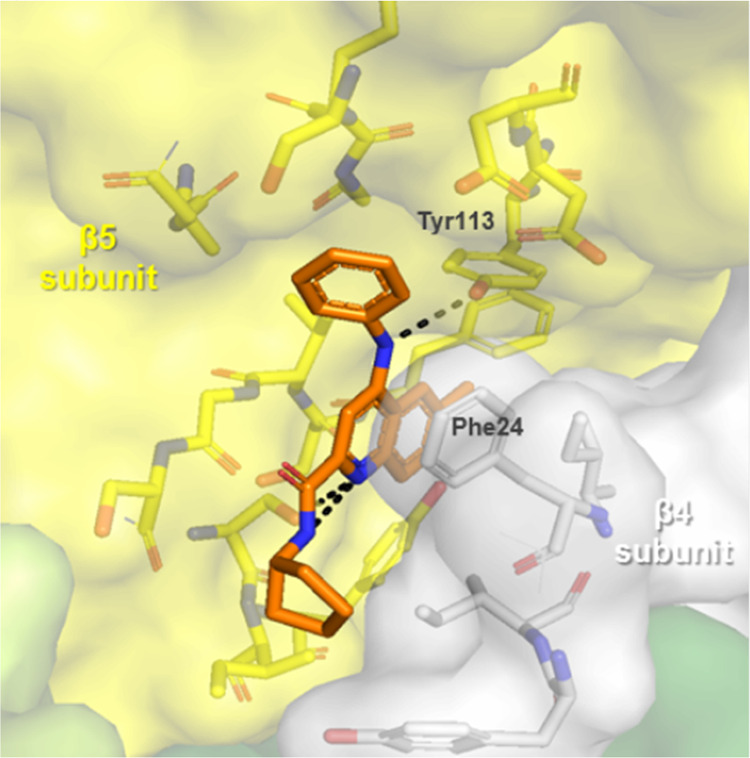
Cryo-EM structure of compound **2**. The atomic model has been deposited in the Protein Data Bank with the accession code PDB ID 7ZYJ. The 260,000 particles in the best two-dimensional (2D) classes were used for three-dimensional (3D) refinement using C2-symmetry. The resulting refinement of best particles gave rise to a reconstruction with an overall resolution of 2.8 Å.

We could rationalize the improvement through the use of computational modeling and the subsequent cryo-EM structure of compound **2** bound with *L. tarentolae* 20S proteasome. The newly introduced N–H (R4) is masked in an intramolecular H-bond, retaining the desired permeability properties, while at the same time restricting the conformation of the tail-piece (R3) to fit into the channel pocket of the binding site. The main interactions responsible for the potency were hypothesized to be the aniline N–H interaction with Tyr113 and noncovalent molecular interactions including π-stacking of the quinoline aromatic system with Phe24 (Figure 1).

Upon identifying secondary amides as more active, we screened a variety of different amide tail-pieces (R3/R4, Tables 1 and 4). The initially explored aliphatic carbocycles suffered from low metabolic stability. We therefore increased the polarity of the side chain, leading us to explore aminopropanediol-derived tail-pieces. This allowed us to identify compounds with improved activity and stability while maintaining a favorable efflux profile (*e.g.*, compound **3**). Based on that initial success, we investigated derivatives such as aminofluoropropanols and aminomethoxypropanols (*e.g.*, compounds **4**–**7**). We next turned our attention to the impact of different head-pieces (R2), as the stereo-electronic properties of the aniline should have a dramatic effect on potency due to the involvement of this vector in the primary protein interaction responsible for activity. We found that electron-poor anilines, particularly those containing an *ortho*-fluorine, greatly improved potency as well as metabolic stability. By introducing an additional nitrogen to the quinoline core leading to a 1,7-naphthyridine, we could improve solubility and liver microsome clearance in all species (compare compounds **3** and **11**). 1,5- and 1,8-Naphthyridines suffered from loss of activity (see the Supporting Information). The introduction of a basic amine into the amide side chain of a 1,7-naphthyridine led to compound **10**, which displayed improved activity and balanced *in vitro* ADME properties. Due to our initial success with aminopropanediol-derived tail-pieces in the quinoline scaffold, we attempted to transfer the SAR to the 1,7-naphthyridine core. Unfortunately, this led to a decrease in activity, low permeability, and high efflux as seen in compound **11**.

The application of the cryo-EM structure allowed us to identify the minimum pharmacophore. We found that vinyl- and ethoxy-pyridines show good activity and balanced physicochemical properties (compounds **13**–**15**, Table 2). Although the pyridines were identified as the minimal pharmacophore required for *T. b. brucei* growth inhibition, we could not achieve single-digit nanomolar potency. Moreover, these compounds showed moderate to high liver microsomal clearance, indicating potential issues with *in vivo* pharmacokinetics. Interestingly, all compounds from the series displayed high permeability and low efflux, likely due to their lower molecular weight and smaller size.

**Table 2:**
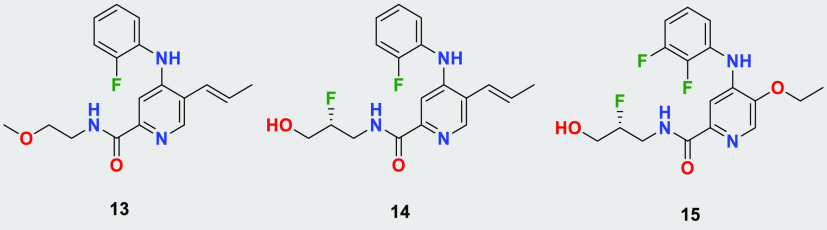
SAR in Pyridine Series^a^

compound	*T. b. brucei* EC_50_ [nM]	*T. b. brucei* proteasome IC_50_/human proteasome [nM]	LM CL_int_ M/R/H [μL/min/mg]	MDCK-MDR1 A to B (ER) [10^–6^ cm/s]
**13**	46	17/>10 000	394/114/62	13.5 (0.6)
**14**	13	19/>10 000	91/48/<25	12.8 (0.6)
**15**	60		95/43/<25	13.8 (0.7)

a Details on the biochemical and cellular assay as well as on clearance (Cl_int_) and permeability (MDCK-MDR1) can be found in the Experimental Section.

**Figure 2:**
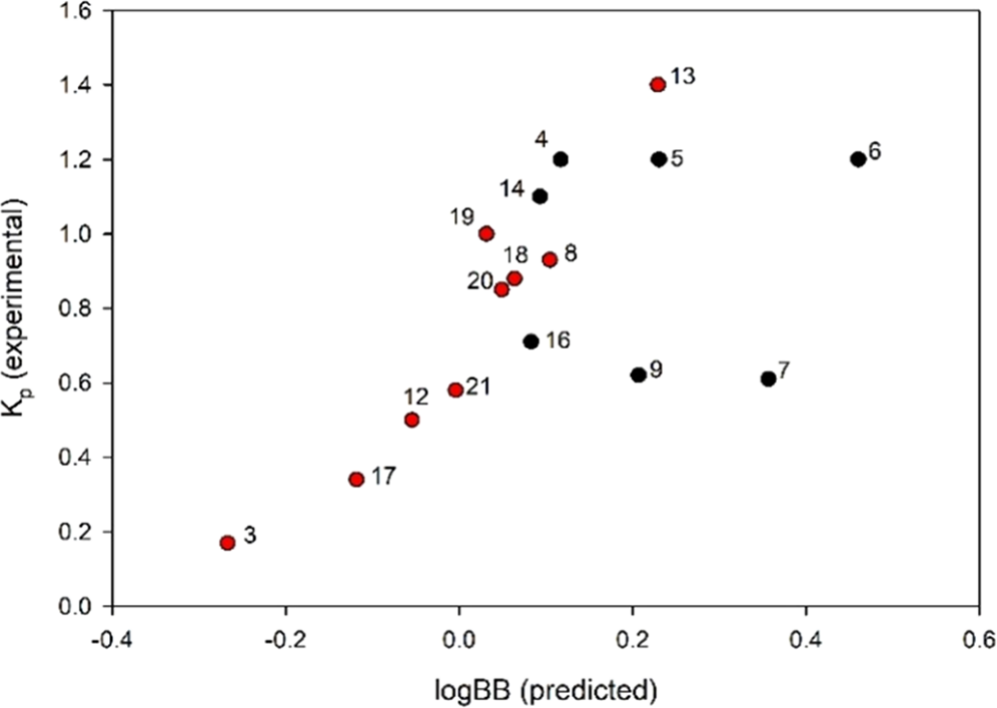
Correlation of log BB and measured *K*_p_ (@ 5 min time point), red: initial set of compounds to build model, black: compounds prioritized based on model.^25^

The most promising compounds were selected for mouse brain pharmacokinetics (PK), plasma protein binding (PPB), and brain tissue binding (BTB) measurements to determine the total and unbound brain-to-plasma ratio (*K*_p_ and *K*_p,uu_). In keeping with the 3R principles with respect to animal welfare (Reduction, Refinement, Replacement), we sought an *in silico* model capable of predicting *K*_p_ to minimize the number of animal experiments required to identify a compound with satisfactory brain penetration. Initially, we found that a number of *in silico* models, including the central nervous system multiparametric optimization (CNS MPO) model,^22,23^ did not correlate with our experimental results. However, when we employed a model provided through StarDrop^24^ we found better qualitative correlation between the predicted log BB (blood-to-brain ratio) and the experimentally measured *K*_p_ (*r*^2^ = 0.91)^25^ for a variety of compounds across different chemotypes (see Figure 2 and Table 3). The model allowed us to predict *K*_p_ prior to synthesis to prioritize compounds with higher predicted log BB values (*i.e.*, log BB – 0.1, which translated to experimental *K*_p_ > 0.3 in mouse in our initial validation set), which helped rapid SAR generation of compounds in the desired property space.

**Table 3:** Predicted and Measured *K*_p_ and *K*_p,uu_ for Modeling and Follow-Up Set of Compounds^a^

compound	log BB (predicted)	*K*_p_ (measured)	*f*_u_ plasma mouse [%]	*f*_u_ rat brain [%]	*K*_p,uu_
**3**	–0.27	0.17	3.0	4.0	0.22
**4**	0.11	1.2	2.0	1.5	0.90
**5**	0.23	1.2	0.8	0.6	0.90
**6**	0.46	1.2	1.2	0.7	0.70
**7**	0.36	0.61	1.0	0.9	0.55
**8**	0.10	0.93	5.5	3.0	0.51
**9**	0.21	0.62	7.3	4.3	0.37
**12**	–0.05	0.50	4.5	4.0	0.44
**13**	0.23	1.4	3.9	3.0	1.1
**14**	0.09	1.1	7.6	4.1	0.59
**16**	0.08	0.71	2.3	4.3	1.3
**17**	–0.12	0.34	10.3	8.7	0.28
**18**	0.06	0.88	8.7	8.8	0.89
**19**	0.03	1.0	11.8	5.6	0.47
**20**	0.05	0.85	1.8	1.5	0.71
**21**	0.00	0.58	4.9	4.6	0.54

a *K*_p,uu_ was calculated using measured *K*_p_, *f*_u,brain_ from BTB and *f*_u,plasma_ from PPB using the following equation: .

After investigating potency, *in vitro* ADME properties and determining *K*_p,uu_ for compounds in the quinoline, naphthyridine, and pyridine series, we selected the most promising leads for PK studies in rodents (Tables 1–4). We found that all compounds generally had low to moderate clearance and that the *in vivo* clearance in the mouse was indeed higher than that in the rat, as predicted by *in vitro* liver microsomes (Table 5). Compounds **5**, **6**, and **7** had the best *in vivo* profiles in both rodent species, exhibiting low clearance and good oral exposures with good to excellent oral bioavailability at low doses. However, they also exhibited high plasma protein binding in mouse with free fractions of <1%. Due to the limitations of the PPB and the BTB assays with compounds that show >99% binding, we advanced tool compounds with <99% binding to avoid additional challenges with respect to establishing *in vitro–in vivo* correlation (IVIVC) for unbound clearance (Cl_u_) and PKPD for unbound brain-to-plasma ratios (*K*_p,uu_) required to achieve complete clearance of parasites. As a result, compound 4 was initially selected to establish PKPD for the quinoline scaffold.

**Table 4:**
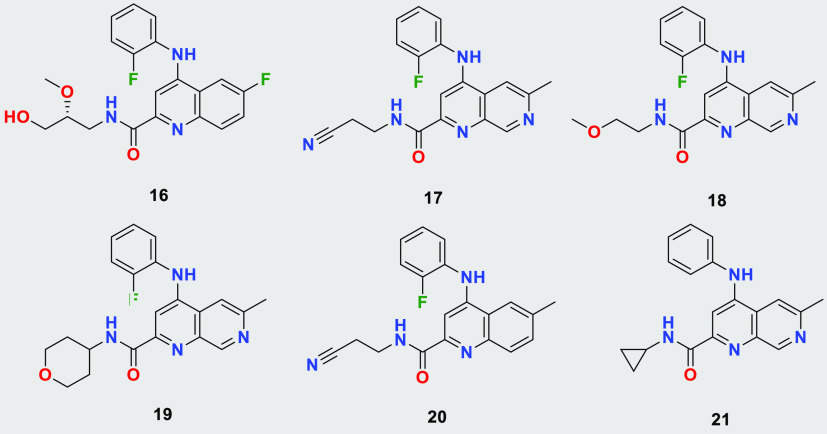
Structures of Tested Quinolines and Naphthyridines^a^

compound	*T. b. brucei* EC_50_ [nM]	LM CL_int_ M/R/H [μL/min/mg]	MDCK-MDR1 A to B (ER) [10^–6^ cm/s]
**16**	37	43/43/<25	7.5 (1.0)
**17**	94	87/<25/<25	13.2 (0.8)
**18**	40	151/52/<25	20.0 (0.6)
**19**	95	51/46/<25	18.2 (0.7)
**20**	14	588/59/40	9.2 (0.5)
**21**	53	414/62/55	12.4 (0.6)

a Details on the biochemical and cellular assay as well as on clearance (Cl_int_) and permeability (MDCK-MDR1) can be found in the Experimental Section.

**Table 5:** ADME and PK Data for Lead Compounds^a^

cmp	**4**	**5**	**6**	**7**	**9**	**14**
solubility pH 6.8 μM	120	22	7	12	150	370
LM CL_int_ (*m*/*r*) [μL/min/mg]	145/37	65/<25	55/<25	79/66	84/28	91/48
LM scaled CL_int_ [mL/min/kg] (*m*/*r*)	571/67	256/45	217/45	311/119	331/50	358/86
*f*_u_ plasma [%] (*m*/*r*)	1.4/3.9	0.6/1.3	0.9/1.4	1.0/1.6	7.3/10.6	7.6/20.1
CL_p_ [mL/min/kg] (*m*/*r*)	37/22	12/5	19/7	17/9	14/19	66/29
CL_u_ [mL/min/kg] (*m*/*r*)	2643/564	2000/385	2111/500	1700/563	192/179	868/144
AUC_PO_ [μM·h] (*m*/*r*)	3.6/6.3	4.9*/16*	6.8/23	5.2*/17*	5.4*/14	0.8/2.5
AUC_IV_ [μM·h] (*m*/*r*)	1.2/2.0	3.2/7.8	2.3/6.3	2.3/4.4	3.3/2.5	0.7/1.6
%F *m*/*r*	58/63	50*/69*	60/76	76*/128*	53*/109	22/30

a Solubility: Miniaturized shake flask solubility as described in the Experimental Section; Cl_int_ assay described in the Experimental Section; *f*_u,plasma_ from PPB; LM scaled Cl_int_ = (LM Cl_int_·SF1·SF2)/1000 with SF1 (mg protein per g liver) *m*,*r* = 45, SF2 (g liver per kg BW); *m* = 87.5, SF2 *r* = 40. Unbound clearance: Cl_u_ = Cl_p_/*f*_u_, with Cl_p_ observed clearance. Mouse and rat PK 1 mpk IV/5 mpk PO. *(**5, 7, 9** PK for 1 mpk IV and 3 mpk PO).

Therefore, we evaluated compound **4** in a stage I HAT mouse efficacy model, where mice were infected with *T. b. brucei* STIB975 and treated from day 3 to day 6 after the infection was established.^26−29^ Compound **4** showed full cure at 3 and 10 mg/kg (mpk) bid as well as 10 mpk qd. At the lowest dose of 1 mpk bid, four out of six animals were cured (Table 6). Compounds **5**, **6**, and **7** from the quinoline series were subsequently evaluated at 3 mpk qd and with the exception of **5** all achieved complete cure of the stage I infection at this low dose (Table 6). However, compounds from the naphthyridine series showed partial cure only at higher doses of 10 mpk bid (data not shown). We hypothesized that higher doses were required due to the lower potency and therefore higher concentrations needed to be achieved to see a parasiticidal effect. Despite displaying good PK properties in mouse, compounds such as **9** were not able to achieve and maintain the required concentrations for cure for a long enough period of time. We were also able to demonstrate sterile cure with a compound from the pyridine series (*e.g.*, compound **14**) at 30 mpk bid with 4 d dosing. Due to **14** being 5-fold more potent than **9**, lower concentrations were required. However, compound **14** is 4-fold less potent compared to **4** and displays inferior mouse PK with lower exposures, requiring bid dosing and higher doses to achieve cure (see the Supporting Information).

**Table 6:**
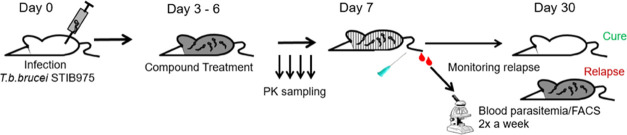
Efficacy of Proteasome Inhibitors in Hemolymphatic Mouse Model^a^

cmp	**4**	**4**	**4**	**4**	**5**	**6**	**7**
dose [mg/kg]	10 bid	3 bid	1 bid	10 qd	3 qd	3 qd	3 qd
cure	6/6	6/6	4/6	6/6	5/6	6/6	6/6

a cmp: compound; bid: twice a day; qd: once a day.

With stage I data in hand, we set out to predict curative doses for the stage II efficacy model. Animals were infected with *T. b. brucei* and allowed for infection to reach the brain. Treatment started from day 21 to day 27 post-infection.^26−29^ It was hypothesized that we could use *K*_p,uu_ to translate the results from our stage I study to the stage II study. Our model compound **4** showed a dose-dependent response. While we did not see cure with 10 mpk bid, we were able to cure one animal at 30 mpk qd, two animals at 60 mpk qd, and ultimately all six animals with 150 mpk qd (Table 7). Compounds **5** and **6** also achieved full cure at 100 mpk qd doses. To our delight, our frontrunner compound **7** achieved cure at a dose of only 15 mpk bid in the CNS model, likely due to superior potency as well as PK properties.

**Table 7:**
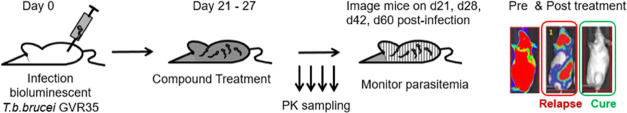
Efficacy of Proteasome Inhibitors in Meningo-encephalic Mouse Model^a^

cmp	**4**	**4**	**4**	**4**	**5**	**6**	**7**
dose [mg/kg]	10 bid	30 qd	60 qd	150 qd	100 qd	100 qd	15 bid
cure	0/6	1/6	2/6	6/6	6/6	6/6	6/6

a cmp: compound; bid: twice a day; qd: once a day.

Further *in vitro* safety profiling established that the 6-methyl substituent on the quinoline ring of compound **4** had genotoxic potential in the TK6 *in vitro* mammalian cell gene mutation assay, making it undesirable for further development. Although the 6-bromo compound **5** was negative in the absence of rat liver S9, a positive signal resulted in the presence of rat liver S9. Fortunately, 6-chloro quinolines **6** and **7** did not show this genotoxic potential (Table 8). During the lead optimization campaign, we also monitored the hERG binding and automated patch clamp (q-patch) signal to address any proarrhythmic potential of the compounds linked to the inhibition of this cardiac ion channel. We found that the key to minimizing binding was the 6-substituent on the quinoline. While methyl-, bromo-, and iodo-substituents showed a higher risk for hERG inhibition, hERG binding as well as inhibition potential was attenuated by fluoro- and chloro-substituents. Compounds, in general, represent high risk for ventricular arrhythmias if the safety margin (EC_50_/free *C*_max_) for the hERG channel is less than 30.^30^ Considering the free *C*_max_ in mouse at the efficacious dose, compound **7** showed the best safety profile with respect to a potential hERG liability (Table 8) and was therefore chosen to be progressed into a 7-day oral gavage *in vivo* pilot toxicology study. In this study, the bone marrow was identified as a target organ of toxicity but only at the highest dose tested (300 mg/kg/day). The exposure multiples between the efficacious dose (15 mg/kg bid) and the rat TK at 100 and 300 mg/kg are approximately 5-fold and 15-fold, respectively.

**Table 8:** *In Vitro* Safety Profiling of Proteasome Inhibitors^31^ a

cmp	**4**	**5**	**6**	**7**
MNT −S9	positive	negative	negative	negative
MNT +S9	negative	positive	negative	negative
hERG binding [μM]	10.9	4.1	13.8	>30
q-patch [μM]	7.8	0.9	1.9	12.7
efficacious dose [mg/kg]	150 qd	100 qd	100 qd	15 bid
*fC*_max_[μM]	0.22	0.12	0.20	0.07
TI (hERG)	50	33	70	403
TI (q-patch)	36	7	10	170

a cmp: compound; MNT: micronucleus test; +S9: in the presence of the rat liver S9 fraction; −S9: in the absence of rat liver S9 fraction, hERG: human ether-a-go-go-related gene; q-patch: automated patch clamp; TI: therapeutic index.

In summary, we have identified and characterized a selective 20S proteasome inhibitor to combat human African trypanosomiasis. The program started out with a singleton HTS hit that exhibited superior physicochemical properties required for brain penetration. Guided by a cryo-EM structure of an active compound bound to the proteasome, we were able to rapidly optimize the potency of candidate compounds. We employed a StarDrop model to help predict the brain-to-plasma partition coefficient *K*_p_ and used these data to select compounds for synthesis, PK and efficacy studies. We ultimately identified a candidate molecule **7**, that cured a stage II mouse model of HAT. Therefore, we believe that this molecule has the potential to be a clinical candidate for African sleeping sickness.

## Experimental Section

All materials and reagents used were of the best commercially available grade and used without further purification. Normal-phase column chromatography was carried out using prepacked silica gel cartridges on a Combiflash Rf separation system by Teledyne ISCO. ^1^H NMR spectra were determined on a Varian 400 or Bruker 300 or 400 and 500 MHz NMR spectrometers. The following abbreviations are used: s = singlet, d = doublet, dd = doublet of doublets, t = triplet, q = quartet, m = multiplet, bs = broad singlet. Preparative HPLC was performed on a Waters Prep HPLC system using a C18 reversed-phase column eluting with gradient mixtures of water/acetonitrile containing a modifier 0.05% trifluoroacetic acid. HPLC analysis showed that all compounds were >95% pure. The purity of the final compounds was confirmed by UPLC-MS and UPLC. Additionally, all final compounds presented with *in vivo* data have been confirmed by ^1^H NMR, LCMS, and HRMS in the Supporting Information.

### *N*-Cyclopentyl-6-methyl-4-(phenylamino)quinoline-2-carboxamide (**2**)

In a microwave vial 4-chloro-*N*-cyclopentyl-6-methylquinoline-2-carboxamide (61 mg, 0.21 mmol) was dissolved in methanol (1.0 mL). Aniline (0.039 mL, 0.43 mmol) and *p*-toluenesulfonic acid monohydrate (1.0 mg, 5.3 μmol) were added to the solution, and the vial was sealed and placed in the microwave. The reaction was heated to 125 °C for 40 min. The reaction mixture was filtered through a 0.45 μm poly(tetrafluoroethylene) syringe-tip filter, and the product was purified by reversed-phase HPLC using 0.1% trifluoroacetic acid water and acetonitrile to give *N*-cyclopentyl-6-methyl-4-(phenylamino)quinoline-2-carboxamide (20 mg, 21%) as a pale yellow solid. LCMS (ESI): *m*/*z* = 346.4 [M + H]^+^; ^1^H NMR (500 MHz, DMSO-*d*_6_) δ 9.01 (s, 1H), 8.46 (s, 1H), 8.12 (d, *J* = 8.7 Hz, 1H), 7.85 (d, *J* = 8.6 Hz, 1H), 7.58 (t, *J* = 7.7 Hz, 2H), 7.51 (d, *J* = 7.8 Hz, 2H), 7.40 (d, *J* = 11.6 Hz, 2H), 4.24–4.20 (m, 1H), 2.58 (s, 3H), 2.02–1.88 (m, 2H), 1.70 (ddd, *J* = 6.9, 4.4, 2.3 Hz, 2H), 1.62–1.51 (m, 4H).

### Example 1 (Details in the Supporting Information)

#### (*R*)-*N*-(2,3-Dihydroxypropyl)-4-((2-fluorophenyl)amino)-6-methylquinoline-2-carboxamide (**3**)

To a solution of 4-((2-fluorophenyl)amino)-6-methylquinoline-2-carboxylic acid (60 mg, 0.20 mmol) and *N*,*N*-diisopropylethylamine (0.14 mL, 0.81 mmol) in dimethylformamide (1.0 mL) was added pivaloyl chloride (0.050 mL, 0.41 mmol), and the reaction was stirred at room temperature for 10 min. (*R*)-3-Aminopropane-1,2-diol (46 mg, 0.51 mmol) was then added, and the reaction was stirred at room temperature for 1 h. The reaction mixture was then partitioned between dichloromethane and water, the organic layer was isolated and concentrated, and the product was purified by reversed-phase HPLC using 0.05% formic acid in water and acetonitrile to give (*R*)-*N*-(2,3-dihydroxypropyl)-4-((2-fluorophenyl)amino)-6-methylquinoline-2-carboxamide (27 mg, 0.074 mmol, 36% yield) as a white solid. LCMS (ESI): *m*/*z* = 370.4 [M + H]^+^; ^1^H NMR (500 MHz, DMSO-*d*_6_) δ 9.05 (s, 1H), 8.66 (t, *J* = 6.0 Hz, 1H), 8.29 (s, 1H), 7.90 (d, *J* = 8.6 Hz, 1H), 7.65 (dd, *J* = 8.8, 1.8 Hz, 1H), 7.50 (td, *J* = 7.9, 1.9 Hz, 1H), 7.46–7.30 (m, 3H), 7.06 (d, *J* = 2.9 Hz, 1H), 4.97 (d, *J* = 4.8 Hz, 1H), 4.66 (t, *J* = 5.7 Hz, 1H), 3.68–3.57 (m, 1H), 3.51 (ddd, *J* = 13.4, 6.6, 4.6 Hz, 1H), 3.44–3.36 (m, 1H), 3.32–3.27 (m, 1H), 3.23–3.14 (m, 1H), 2.57 (s, 3H).

Compound **1** was prepared according to the procedure of Example 1, using aniline and pyrrolidine as the starting material to give (6-methyl-4-(phenylamino)quinolin-2-yl)(pyrrolidin-1-yl)methanone as a pale yellow solid. LCMS (ESI): *m*/*z* = 332.1 [M + H]^+^; ^1^H NMR (300 MHz, chloroform-*d*) δ 7.94 (d, *J* = 8.1 Hz, 1H), 7.70 (s, 1H), 7.55 (dd, *J* = 8.4, 1.8 Hz, 1H), 7.45–7.7.38 (m, 3H), 7.35–7.29 (m, 2H), 3.17 (t, *J* = 7.2 Hz, 1H), 6.64 (s, 1H), 3.75 (t, *J* = 6.8 Hz, 2H), 3.66 (t, *J* = 6.8 Hz, 2H), 2.58 (s, 3H), 2.0–1.85 (m, 4H).

Compound **4** was prepared according to the procedure of Example 1, differing by the last peptide coupling step: To a solution of 4-((2-fluorophenyl)amino)-6-methylquinoline-2-carboxylic acid (2.0 g, 6.8 mmol) and (*R*)-3-amino-2-fluoropropan-1-ol (0.94 g, 10 mmol) in dimethylformamide (volume: 12 mL) were added triethylamine (2.8 mL, 20 mmol) and propylphosphonic anhydride in ethyl acetate (6.0 mL, 13 mmol), and the resulting mixture was stirred at room temperature for 30 min. The reaction was diluted with ethyl acetate, washed with water and brine, dried over magnesium sulfate, filtered, and concentrated. The resulting crude oil was purified by column chromatography (30–90% [9:1 ethyl acetate:methanol] in heptanes) to afford the title compound as an off-white solid (1.5 g, 4.1 mmol, 60% yield). LCMS (ESI): *m*/*z* = 372.3 [M + H]^+^; ^1^H NMR (500 MHz, chloroform-*d*) δ 8.71–8.54 (m, 1H), 7.96 (d, *J* = 8.6 Hz, 1H), 7.84 (s, 1H), 7.75 (s, 1H), 7.60 (dd, *J* = 8.7, 1.8 Hz, 1H), 7.55 (td, *J* = 8.0, 1.7 Hz, 1H), 7.25–7.19 (m, 2H), 7.19–7.13 (m, 1H), 6.67 (s, 1H), 4.82–4.66 (m, 1H), 3.97–3.66 (m, 4H), 3.49 (s, 1H), 2.61 (d, *J* = 0.9 Hz, 3H).

Compound **5** was prepared according to the procedure of Example 1, using 4-bromoaniline as the starting material, peptide coupling step: To a solution of 6-bromo-4-((2-fluorophenyl)amino)quinoline-2-carboxylic acid (30 mg, 0.083 mmol) and (*R*)-3-amino-2-fluoropropan-1-ol (11.60 mg, 0.125 mmol) in DMF (volume: 1 mL) were added triethylamine (0.035 mL, 0.249 mmol) and propylphosphonic anhydride in DMF (0.097 mL, 0.166 mmol), and the resulting mixture was stirred at room temperature for 30 min. The mixture was filtered through a syringe-tip filter and purified by reversed-phase column chromatography to afford (*R*)-6-bromo-*N*-(2-fluoro-3-hydroxypropyl)-4-((2-fluorophenyl)amino)quinoline-2-carboxamide (21.1 mg, 0.048 mmol, 57.6% yield) as a pale yellow solid. LCMS (ESI): *m*/*z* = 436.2 [M + H]^+^; ^1^H NMR(400 MHz, DMSO-*d*_6_) δ 9.27 (s, 1H), 8.91 (t, *J* = 6.1 Hz, 1H), 8.79 (d, *J* = 1.6 Hz, 1H), 7.94 (d, *J* = 1.2 Hz, 2H), 7.57–7.31 (m, 4H), 7.07 (d, *J* = 2.8 Hz, 1H), 4.67 (dt, *J* = 54.2, 7.3 Hz, 1H), 3.71–3.45 (m, 5H).

Compound **6** was prepared according to the procedure of Example 1, using 4-chloroaniline as the starting material and (*R*)-3-amino-2-fluoropropan-1-ol to give (*R*)-6-chloro-*N*-(2-fluoro-3-hydroxypropyl)-4-((2-fluorophenyl)amino)quinoline-2-carboxamide (18% yield) as a pale yellow solid. LCMS (ESI): *m*/*z* = 392.4 [M + H]^+^; ^1^H NMR (500 MHz, DMSO-*d*_6_) δ 9.27 (s, 1H), 8.93 (t, *J* = 6.2 Hz, 1H), 8.65 (d, *J* = 2.3 Hz, 1H), 8.03 (d, *J* = 9.0 Hz, 1H), 7.84 (dd, *J* = 9.0, 2.3 Hz, 1H), 7.58–7.32 (m, 4H), 7.08 (d, *J* = 2.8 Hz, 1H), 4.79–4.55 (m, 1H), 3.68–3.53 (m, 4H).

Compound **7** was prepared according to the procedure of Example 1, using 4-chloroaniline as the starting material and (*R*)-3-amino-2-methoxypropan-1-ol to give (*R*)-6-chloro-4-((2,3-difluorophenyl)amino)-*N*-(3-hydroxy-2-methoxypropyl)quinoline-2-carboxamide (53% yield) as an off-white solid. LCMS (ESI): *m*/*z* = 422.3 [M + H]^+^; ^1^H NMR (500 MHz, chloroform-*d*) δ 8.50 (s, 1H), 8.03 (dd, *J* = 9.0, 0.5 Hz, 1H), 7.98 (d, *J* = 2.2 Hz, 1H), 7.90 (s, 1H), 7.71 (dd, *J* = 9.0, 2.2 Hz, 1H), 7.31–7.26 (m, 1H), 7.18–7.12 (m, 1H), 7.06–6.97 (m, 1H), 6.58 (s, 1H), 3.82 (ddd, *J* = 14.3, 7.2, 4.0 Hz, 1H), 3.69–3.58 (m, 3H), 3.53–3.50 (m, 1H), 3.49 (s, 3H).

Compound **16** was prepared according to the procedure of Example 1, using 4-fluoroaniline as the starting material and (*R*)-3-amino-2-methoxypropan-1-ol to give (*R*)-6-fluoro-4-((2-fluorophenyl)amino)-*N*-(3-hydroxy-2-methoxypropyl)quinoline-2-carboxamide. Peptide coupling step: To a solution of 6-fluoro-4-((2-fluorophenyl)amino)quinoline-2-carboxylic acid (0.572 g, 1.699 mmol) and (*R*)-3-amino-2-methoxypropan-1-ol (0.196 g, 1.869 mmol) in DMF (volume: 4 mL) were added triethylamine (0.710 mL, 5.10 mmol) and propylphosphonic anhydride in ethyl acetate (1.983 mL, 3.40 mmol), and the resulting mixture was stirred at room temperature for 30 min. The reaction was diluted with ethyl acetate, washed with water and brine, dried over magnesium sulfate, filtered, and concentrated. The resulting crude oil was purified by column chromatography (30–50% [3:1 AcOEt:EtOH]/heptanes). Combined fractions were concentrated and lyophilized to afford (*R*)-6-fluoro-4-((2-fluorophenyl)amino)-*N*-(3-hydroxy-2-methoxypropyl)quinoline-2-carboxamide (425 mg, 1.70 mmol, 64%) as an off-white solid. LCMS (ESI): *m*/*z* = 388.3 [M + H]^+^; ^1^H NMR (500 MHz, DMSO-*d*_6_) δ 9.10 (s, 1H), 8.70 (t, *J* = 5.8 Hz, 1H), 8.30 (dd, *J* = 10.8, 2.8 Hz, 1H), 8.07 (dd, *J* = 9.3, 5.6 Hz, 1H), 7.74 (ddd, *J* = 9.3, 8.1, 2.8 Hz, 1H), 7.54–7.32 (m, 4H), 7.08 (d, *J* = 2.8 Hz, 1H), 3.53–3.42 (m, 3H), 3.40–3.36 (m, 2H), 3.35 (s, 4H).

Compound **20** was prepared according to the procedure of Example 1, using 3-aminopropanenitrile to give *N*-(2-cyanoethyl)-4-((2-fluorophenyl)amino)-6-methylquinoline-2-carboxamide.

Peptide coupling step: To 4-((2-fluorophenyl)amino)-6-methylquinoline-2-carboxylic acid (20 mg, 0.060 mmol) in DCM (volume: 1 mL) were added 2-(3*H*-[1,2,3]triazolo[4,5-*b*]pyridin-3-yl)-1,1,3,3-tetramethylisouronium hexafluorophosphate(V) [HATU] (28.6 mg, 0.075 mmol) and *N*-ethyl-*N*-isopropylpropan-2-amine (0.052 mL, 0.301 mmol). The reaction mixture was stirred at room temperature for 15 min before adding 3-aminopropanenitrile (6.32 mg, 0.090 mmol). The resulting mixture was stirred for an additional 15 min. Additional equivalents for HATU, *N*-ethyl-*N*-isopropylpropan-2-amine, and aminopropanenitrile were needed to achieve full conversion. The reaction mixture was concentrated to dryness. Redissolved in MeOH and filtered through a syringe-tip filter. The crude material was purified by reversed-phase chromatography to afford *N*-(2-cyanoethyl)-4-((2-fluorophenyl)amino)-6-methylquinoline-2-carboxamide (6.6 mg, 0.019 mmol, 31% yield) as an off-white solid. LCMS (ESI): *m*/*z* = 349.4 [M + H]^+^; ^1^H NMR (500 MHz, DMSO-*d*_6_) δ 9.10–9.02 (m, 2H), 8.29 (t, *J* = 1.6 Hz, 1H), 7.93 (d, *J* = 8.6 Hz, 1H), 7.67 (dd, *J* = 8.8, 1.8 Hz, 1H), 7.50 (td, *J* = 7.9, 1.8 Hz, 1H), 7.47–7.32 (m, 3H), 7.05 (d, *J* = 2.9 Hz, 1H), 3.55 (q, *J* = 6.5 Hz, 2H), 2.81 (t, *J* = 6.6 Hz, 2H), 2.58 (d, *J* = 0.9 Hz, 3H).

### Example 2 (Details in the Supporting Information)

Compound **8**: 4-Chloro-*N*-cyclopropyl-6-methyl-1,7-naphthyridine-2-carboxamide (28 mg, 0.11 mmol), 2-fluoroaniline (18 mg, 0.16 mmol), sodium *tert*-butoxide (12 mg, 0.13 mmol), and BrettPhos Pd G3 (1.9 mg, 2.1 μmol) were suspended in dioxane (1 mL). The mixture was stirred at 100 °C for 2 h in the microwave. The crude mixture was diluted with methanol and filtered through a 0.45 μm poly(tetrafluoroethylene) syringe-tip filter. The product was purified by reversed-phase HPLC using 0.1% trifluoroacetic acid water and acetonitrile to give *N*-cyclopropyl-4-((2-fluorophenyl)amino)-6-methyl-1,7-naphthyridine-2-carboxamide (2.2%) as a tan solid. LCMS (ESI): *m*/*z* = 337.4 [M + H]^+^; ^1^H NMR (500 MHz, methanol-*d*_4_) δ 9.24 (s, 1H), 8.12 (s, 1H), 7.50 (t, *J* = 8.0 Hz, 1H), 7.43–7.37 (m, 1H), 7.34 (dd, *J* = 13.3, 5.3 Hz, 3H), 2.89 (tt, *J* = 7.6, 4.0 Hz, 1H), 2.77 (s, 3H), 0.93–0.81 (m, 2H), 0.78–0.67 (m, 2H).

### Example 3 (Details in the Supporting Information)

Compound **17**: Propylphosphonic anhydride in ethyl acetate (458 mg, 0.720 mmol) and triethylamine (0.201 mL, 1.440 mmol) were added to a solution of 4-((2-fluorophenyl)amino)-6-methyl-1,7-naphthyridine-2-carboxylic acid (107 mg, 0.360 mmol) in DCM (volume: 2 mL). The reaction was stirred at room temperature for 15 min before 3-aminopropanenitrile (31.5 mg, 0.450 mmol) was added. The mixture was stirred for an additional 45 min at room temperature. Water and DCM were added to the reaction mixture. The phases were separated, and the aqueous layer was extracted with DCM. The combined organic layers were dried over magnesium sulfate, filtered, and concentrated. The resulting crude oil was purified by column chromatography (30–100% EtOAc in heptanes). Combined fractions were concentrated and lyophilized to afford *N*-(2-cyanoethyl)-4-((2-fluorophenyl)amino)-6-methyl-1,7-naphthyridine-2-carboxamide (35 mg, 0.099 mmol, 28%) as an off-white solid. LCMS (ESI): *m*/*z* = 350.3 [M + H]^+^; ^1^H NMR (500 MHz, methanol-*d*_4_) δ 9.28 (s, 1H), 8.14 (s, 1H), 7.50 (t, *J* = 8.0 Hz, 1H), 7.43–7.29 (m, 4H), 3.71 (t, *J* = 6.6 Hz, 2H), 2.82 (t, *J* = 6.7 Hz, 2H), 2.78 (s, 3H).

Compound **9** was prepared according to the procedure of Example 3, using 2,6-difluoroaniline as the starting material and cyclopropanamine to give *N*-cyclopropyl-4-((2,6-difluorophenyl)amino)-6-methyl-1,7-naphthyridine-2-carboxamide.

Peptide coupling step: Propylphosphonic anhydride in ethyl acetate (202 mg, 0.317 mmol) and triethylamine (0.088 mL, 0.634 mmol) were added to a solution of 4-((2,6-difluorophenyl)amino)-6-methyl-1,7-naphthyridine-2-carboxylic acid (50 mg, 0.159 mmol) in DCM (volume: 1 mL). The reaction was stirred at room temperature for 15 min before cyclopropanamine (11.32 mg, 0.198 mmol) was added. The mixture was stirred for an additional 45 min at room temperature. Water and DCM were added to the reaction mixture. The phases were separated, and the aqueous layer was extracted with DCM. The combined organic layers were dried over magnesium sulfate, filtered, and concentrated. The resulting crude oil was purified by column chromatography (30–100% EtOAc in heptanes). Combined fractions were concentrated and lyophilized to afford *N*-cyclopropyl-4-((2,6-difluorophenyl)amino)-6-methyl-1,7-naphthyridine-2-carboxamide (10 mg, 0.027 mmol, 17%) as an off-white solid. LCMS (ESI): *m*/*z* = 355.3 [M + H]^+^; ^1^H NMR (500 MHz, methanol-*d*_4_) δ 9.27 (s, 1H), 8.15 (s, 1H), 7.52–7.43 (m, 1H), 7.27–7.19 (m, 2H), 7.17 (d, *J* = 2.0 Hz, 1H), 2.94–2.86 (m, 1H), 2.78 (d, *J* = 1.7 Hz, 3H), 0.85 (t, *J* = 6.7 Hz, 2H), 0.73 (dd, *J* = 4.2, 2.2 Hz, 2H).

Compound **11** was prepared according to the procedure of Example 3, using 2-fluoroaniline as the starting material.

Peptide coupling step: A mixture of 4-((2-fluorophenyl)amino)-6-methyl-1,7-naphthyridine-2-carboxylic acid (25 mg, 0.084 mmol), 2-(3*H*-[1,2,3]triazolo[4,5-*b*]pyridin-3-yl)-1,1,3,3-tetramethylisouronium hexafluorophosphate(V) (38 mg, 0.10 mmol), and (*R*)-3-aminopropane-1,2-diol (9.1 mg, 0.10 mmol) was dissolved in dichloromethane (500 μL). *N*-Ethyl-*N*-isopropylpropan-2-amine (44 μL, 0.25 mmol) was added, and the reaction was stirred at room temperature for 30 min. The mixture was concentrated to dryness, redissolved in dimethyl sulfoxide, and filtered through a 0.45 μm poly(tetrafluoroethylene) syringe-tip filter. The product was purified by reversed-phase HPLC (0.1% formic acid water and acetonitrile) to afford (*R*)-*N*-(2,3-dihydroxypropyl)-4-((2-fluorophenyl)amino)-6-methyl-1,7-naphthyridine-2-carboxamide (13.2 mg, 0.031 mmol, 37% yield) as a yellow solid. LCMS (ESI): *m*/*z* = 371.4 [M + H]^+^; ^1^H NMR (500 MHz, DMSO) δ 9.32 (s, 1H), 9.24 (s, 1H), 8.70–8.67 (m, 1H), 8.22 (s, 1H), 7.51–7.36 (m, 4H), 7.14 (s, 1H), 4.97 (s, 1H), 4.68–4.65 (m, 1H), 3.63–3.59 (m, 1H), 3.53–3.46 (m, 1H), 3.41–3.37 (m, 1H), 3.32–3.28 (m, 1H), 3.25–3.17 (m, 1H), 2.69 (s, 3H).

Compound **12** was prepared according to the procedure of Example 3, using 2-fluoroaniline as the starting material. Peptide coupling step: A mixture of 4-((2-fluorophenyl)amino)-6-methyl-1,7-naphthyridine-2-carboxylic acid (25 mg, 0.084 mmol), 2-(3*H*-[1,2,3]triazolo[4,5-*b*]pyridin-3-yl)-1,1,3,3-tetramethylisouronium hexafluorophosphate(V) (38 mg, 0.10 mmol) and 3-amino-2,2-dimethylpropanenitrile (9.9 mg, 0.10 mmol) was dissolved in dichloromethane (500 μL). *N*-Ethyl-*N*-isopropylpropan-2-amine (44 μL, 0.25 mmol) was added, and the reaction was stirred at room temperature for 30 min. The mixture was concentrated to dryness, redissolved in dimethyl sulfoxide, and filtered through a 0.45 μm poly(tetrafluoroethylene) syringe-tip filter. The product was purified by reversed-phase HPLC (0.1% formic acid water and acetonitrile) to afford *N*-(2-cyano-2-methylpropyl)-4-((2-fluorophenyl)amino)-6-methyl-1,7-naphthyridine-2-carboxamide (13.4 mg, 0.031 mmol, 37% yield) as a yellow solid. LCMS (ESI): *m*/*z* = 378.4 [M + H]^+^; ^1^H NMR (500 MHz, DMSO) δ 9.35 (s, 1H), 9.30 (s, 1H), 9.06 (t, *J* = 6.8 Hz, 1H), 8.25 (d, *J* = 0.9 Hz, 1H), 7.53 (td, *J* = 8.0, 1.8 Hz, 1H), 7.50–7.41 (m, 2H), 7.38 (td, *J* = 7.5, 1.8 Hz, 1H), 7.15 (d, *J* = 2.9 Hz, 1H), 3.52 (d, *J* = 6.8 Hz, 2H), 2.72 (s, 3H), 1.34 (s, 6H).

Compound **18** was prepared according to the procedure of Example 3, using 2-methoxyethan-1-amine, to give the title compound (56% yield) as a brown solid. LCMS (ESI): *m*/*z* = 355.4 [M + H]^+^; ^1^H NMR (400 MHz, chloroform-*d*) δ 9.38 (s, 1H), 8.50–8.40 (m, 1H), 7.95 (s, 1H), 7.59 (s, 1H), 7.58–7.50 (m, 1H), 7.26–7.22 (m, 3H). 6.64 (s, 1H), 3.70–3.68 (m, 2H), 3.62–3.59 (m, 2H), 3.43 (3H), 2.79 (s, 3H).

Compound **19** was prepared according to the procedure of Example 3, using 2-fluoroaniline as the starting material. Peptide coupling step: To a solution of 4-((2-fluorophenyl)amino)-6-methyl-1,7-naphthyridine-2-carboxylic acid (85 mg, 0.29 mmol) in dichloromethane (1.4 mL) were added pivaloyl chloride (53 μL, 0.43 mmol) and triethylamine (120 μL, 0.86 mmol). The mixture was stirred at room temperature for 5 min. Then, tetrahydro-2*H*-pyran-4-amine (36 μL, 0.34 mmol) was added. The reaction was further stirred at room temperature for 5 min. The reaction was then diluted with dichloromethane and water. The phases were separated, and the aqueous layer was further extracted with dichloromethane. The combined organic layers were dried over magnesium sulfate, filtered, and concentrated. The resulting crude oil was purified by column chromatography (0–100% ethyl acetate in heptanes) and lyophilized to afford 4-((2-fluorophenyl)amino)-6-methyl-*N*-(tetrahydro-2*H*-pyran-4-yl)-1,7-naphthyridine-2-carboxamide (76 mg, 0.20 mmol, 69% yield) as a white solid. LCMS (ESI): *m*/*z* = 381.4 [M + H]^+^; ^1^H NMR (500 MHz, CDCl3) δ 9.41 (s, 1H), 8.17 (d, *J* = 8.3 Hz, 1H), 7.97 (s, 1H), 7.62 (s, 1H), 7.56 (t, *J* = 7.5 Hz, 1H), 7.27–7.20 (m, 3H), 6.67 (s, 1H), 4.26–4.14 (m, 1H), 4.06 (d, *J* = 12.3 Hz, 2H), 3.59 (t, *J* = 11.5 Hz, 2H), 2.83 (s, 3H), 2.05 (d, *J* = 12.9 Hz, 2H), 1.75 (qd, *J* = 11.6, 4.5 Hz, 2H).

### Example 4 (Details See the Supporting Information)

Compound **10**: To a solution of 4-((2-fluorophenyl)amino)-*N*-((3*R*,4*S*)-3-fluoropiperidin-4-yl)-6-methyl-1,7-naphthyridine-2-carboxamide (94 mg, 0.24 mmol) in methanol (6.8 mL) were added formaldehyde (0.53 mL, 7.1 mmol) and acetic acid (0.081 mL, 1.4 mmol), and the reaction was stirred at room temperature for 5 min. Sodium triacetoxyborohydride (150 mg, 0.71 mmol) was then added, and the reaction was stirred at room temperature for 30 min. The product was purified by reversed-phase HPLC (0.1% trifluoroacetic acid water and acetonitrile) to give the title compound (59.4 mg, 0.14 mmol, 59% yield) as a white solid. LCMS (ESI): *m*/*z* = 412.4 [M + H]^+^; ^1^H NMR (500 MHz, DMSO-*d*_6_) δ 9.38 (s, 1H), 9.24 (s, 1H), 8.45–8.43 (m, 1H), 8.21 (s, 1H), 7.50–7.30 (m, 4H), 7.07–7.11 (m, 1H), 4.80–4.70 (m, 1H), 4.0–3.80 (m, 1H), 3.10–3.00 (m, 1H), 2.80–2.76 (m, 1H), 2.68 (s, 3H), 2.54 (s, 1H), 2.18 (s, 3H), 2.15–2.00 (m, 1H), 1.95–1.85 (m, 1H), 1.75–1.65 (m, 1H).

### Example 5 (Details in the Supporting Information)

Compound **21**: To ethyl 6-methyl-4-(phenylamino)-1,7-naphthyridine-2-carboxylate (33 mg, 0.11 mmol) in 1,4-dioxane (250 μL) was added cyclopropylamine (11 μL, 0.16 mmol) followed by a 1.0 M solution of lithium bis(trimethylsilyl)amide in THF (322 μL, 0.32 mmol). The reaction was stirred for 15 min at room temperature. The crude mixture was diluted with methanol and filtered through a 0.45 μm poly(tetrafluoroethylene) syringe-tip filter. The product was purified by reversed-phase HPLC (0.1% trifluoroacetic acid water and acetonitrile) to afford *N*-cyclopropyl-6-methyl-4-(phenylamino)-1,7-naphthyridine-2-carboxamide (10.7 mg, 0.024 mmol, 23% yield) as a bright yellow solid. LCMS (ESI): *m*/*z* = 319.3 [M + H]^+^; ^1^H NMR (500 MHz, DMSO) δ 9.43 (s, 1H), 9.27 (s, 1H), 8.77 (d, *J* = 4.8 Hz, 1H), 8.25 (s, 1H), 7.63 (s, 1H), 7.56–7.48 (m, 2H), 7.45–7.39 (m, 2H), 7.32–7.25 (m, 1H), 2.93–2.84 (m, 1H), 2.71 (s, 3H), 0.77–0.66 (m, 4H).

### Example 6 (Details in the Supporting Information)

Compound **13**: A solution of 5-bromo-4-((2-fluorophenyl)amino)-*N*-(2-methoxyethyl)picolinamide (150 mg, 0.41 mmol), (*E*)-prop-1-en-1-ylboronic acid (53 mg, 0.61 mmol) and potassium carbonate (173 mg, 0.82 mmol) in 1,4-dioxane (5.0 mL) and water (2.0 mL) was sparged with argon for 10 min. Then, [1,1′-bis(diphenylphosphino)ferrocene]dichloropalladium(II)-dichloromethane (50 mg, 0.061 mmol) was added. The resulting mixture was heated at 95 °C for 3 h. The reaction was diluted with ethyl acetate and washed with water and brine. The organic layer was dried over anhydrous sodium sulfate and concentrated. The resulting crude oil was purified by column chromatography (0-40% ethyl acetate in hexane) to afford (*E*)-4-((2-fluorophenyl)amino)-*N*-(2-methoxyethyl)-5-(prop-1-en-1-yl)picolinamide (60 mg, 0.18 mmol, 47% yield) as a pale yellow gum. LCMS (ESI): *m*/*z* = 330.4 [M + H]^+^; ^1^H NMR (400 MHz, DMSO) δ 8.55–8.48 (m, 1H), 8.38 (s, 1H), 8.24 (s, 1H), 7.37–7.27 (m, 4H), 7.05 (d, *J* = 3.1 Hz, 1H), 6.74 (dd, *J* = 15.7, 1.4 Hz, 1H), 6.40–6.30 (m, 1H), 3.43–3.38 (m, 4H), 3.25 (s, 3H), 1.94 (dd, *J* = 6.6, 1.7 Hz, 3H).

### Example 7 (Details in the Supporting Information)

Compound **14**: To a solution of (*E*)-4-((2-fluorophenyl)amino)-5-(prop-1-en-1-yl)picolinic acid (50 mg, 0.18 mmol) and (*R*)-3-amino-2-fluoropropan-1-ol (26 mg, 0.28 mmol) in dimethylformamide (800 μL) were added triethylamine (77 μL, 0.55 mmol) and a 50% weight solution of 2,4,6-tripropyl-1,3,5,2,4,6-trioxatriphosphinane 2,4,6-trioxide in dimethylformamide (214 μL, 0.37 mmol). The reaction was stirred at room temperature for 3 h. The mixture was filtered through a 0.45 μm poly(tetrafluoroethylene) syringe-tip filter. The product was purified by reversed-phase HPLC (0.1% formic acid water and acetonitrile) to afford (*R*,*E*)-*N*-(2-fluoro-3-hydroxypropyl)-4-((2-fluorophenyl)amino)-5-(prop-1-en-1-yl)picolinamide (29 mg, 0.082 mmol, 45% yield) as a white solid.

LCMS (ESI): *m*/*z* = 348.3 [M + H]^+^; ^1^H NMR (500 MHz, DMSO) δ 8.74 (t, *J* = 6.2 Hz, 1H), 8.40 (s, 1H), 8.27 (s, 1H), 7.40–7.26 (m, 4H), 7.06 (d, *J* = 3.0 Hz, 1H), 6.75 (d, *J* = 15.7 Hz, 1H), 6.37 (dq, *J* = 15.7, 6.6 Hz, 1H), 4.70–4.55 (m, 1H), 3.63–3.41 (m, 5H), 1.95 (dd, *J* = 6.6, 1.8 Hz, 3H).

### Example 8 (Details in the Supporting Information)

Compound **15**: To a suspension of 4-((2,3-difluorophenyl)amino)-5-ethoxypicolinic acid (29.4 mg, 0.10 mmol) and (*R*)-3-amino-2-fluoropropan-1-ol (13.97 mg, 0.150 mmol) in DMF (volume: 0.5 mL) were added DIPEA (0.052 mL, 0.300 mmol) and HATU (45.6 mg, 0.120 mmol). The mixture was stirred at RT for 1 h. LCMS indicated full conversion. The mixture was diluted with DCM, separated with water, washed with water and brine, dried over Na_2_SO_4_, filtered, and concentrated. The was purified by reversed-phase HPLC (0.1% trifluoroacetic acid water and acetonitrile), lyophilized, and freebased with EtOAc/NaHCO_3_ to afford the desired product (18.6 mg, 0.050 mmol, 50% yield) as a yellow powder. LCMS (ESI): *m*/*z* = 370.2 [M + H]^+^; ^1^H NMR (500 MHz, DMSO-*d*_6_) δ 8.67 (s, 1H), 8.15–8.03 (m, 1H), 7.38–7.07 (m, 4H), 4.72–4.50 (m, 1H), 4.28 (q, *J* = 7.0 Hz, 2H), 3.63–3.42 (m, 4H), 1.42 (t, *J* = 6.9 Hz, 3H).

### Cryo-EM Sample Preparation, Data Acquisition, and Image Processing

Cryo-EM sample preparation and data acquisition methods were as described previously.^32^*L. tarentolae* 20S proteasome (4 mg/mL) was incubated with 60 μM concentration of compound 2 at 4 °C for 20 min; 4 μL aliquots of complex were applied to glow-discharged 300-mesh Quantifoil R 1.2/1.3 grids (Quantifoil, Micro Tools GmbH, and Germany). The grids were glow-discharged for 90 s at 15mA in a PELCO easiGlowTM glow discharger in the presence of pentylamine (Fluka 77060) right before use. Grids were blotted for 3 s and plunged into liquid ethane using Leica EM GP Plunger operated at 4 °C and 85% humidity.

High-resolution images were collected with a Cs-corrected FEI Titan Krios TEM operated at 300 kV equipped with a Quantum-LS Gatan Image Filter (GIF) and recorded on a K2-Summit direct electron detector (Gatan GmbH). Images were acquired automatically (with EPU, Thermo Fisher) in electron-counting mode (nominal post-GIF magnification of ×130,000 and calibrated pixel size of 0.86 Å). Exposures of 7 s were dose-fractionated into 40 frames. The total exposure dose was ∼40 e^–^/Å^2^. Defocus values varied from −0.8 to −2.5 μm.

Micrographs were drift-corrected using UNBLUR^33^ before estimating CTF parameters using CTFFIND4.^34^ Particle picking was carried out using cisTEM.^35^ Picked particles were extracted into boxes of 300 × 300 pixels. Micrographs with severe drift or ice contamination were discarded based upon inspection of the power spectra. A total of 7000 micrographs were acquired from which 1 million particles were extracted for processing using CisTEM software package.^35^ The 260,000 particles in the best 2D classes were used for 3D refinement using C2-symmetry. The resulting refinement of best particles gave rise to reconstruction with an overall resolution of 2.8 Å. The resolution values reported are based on the gold standard Fourier shell correlation curve (FSC) at 0.143 criterion. The cryo-EM structures of 20S proteasome (PDB ID code 6TD5) were manually fitted into the final cryo-EM map using the program Coot.^36^ The resultant atomic model was subjected to multiple cycles of model rebuilding using the program Coot and real space refinement against the map using the program Phenix.^37^ This process resulted in an atomic model of the proteosome–compound 2 complex that fit well into the cryo-EM density. Structural illustrations were prepared with PyMOL (www.pymol.org).

### Biological Profiling

#### High-Throughput Screening

*Leishmania torantolae* parasites were grown to log phase, and cells were harvested by centrifugation and stored at −80 °C freezer. 20S proteasome was purified using the protocol described earlier.^20,32^ The purified proteasome was used for single-point chymotrypsin activity inhibition screen using 3 million compound library. Chymotrypsin activity was measured using a fluorescent probe as described earlier. Hits obtained were further confirmed in dose–response assay.

#### Chymotrypsin Inhibition Activity

20S proteasome was isolated from *T. bruceibrucei* (*T. b. brucei*) Lister 427 by following protocols described earlier.^20^ Chymotrypsin inhibition activity of compounds was measured using rhodamine-labeled fluorogenic chymotrypsin substrate by incubating varying concentrations of compounds with *T. b. brucei* 20S proteasome. IC_50_ was determined using GraphPad Prism software.

#### Parasite Growth Inhibition Studies

Growth inhibition studies for all compounds were carried out using bloodstream form of *T. b. brucei* Lister 427 strain as described earlier.^21,29^ Briefly, 10-point threefold dilutions of compounds with starting concentration of 50 μM were added into 384-well white plates. These plates were incubated for 48 h with 1 × 10^4^/mL of *T. brucei* Lister 427 parasites grown using Hirumi-9 media supplemented with 10% FBS and 10% serum plus. Cell Titer Glo was added to measure the ATP levels as a surrogate for cell viability by quantifying the luminescence. The EC_50_ values were determined using HELIOS software.

### *In Vivo* Pharmacokinetic Analysis

*In vivo* pharmacokinetics (PK) for compounds were generated using BALB/c mice and Wistar rats using standard procedure described elsewhere.^21^ For intravenous PK studies compounds were dosed at 1 mg/kg dose formulated in 75% 2.5 mg/mL PEG300 and 25% D5W (5% dextrose in distilled water). For oral PK studies, compounds were dosed at 5 mg/kg formulated using a simple suspension of 0.5% v/v methyl cellulose tween 80. Each group had three animals, blood samples were collected at six time points post dosing, and compound levels were monitored using LCMS-MS. Various PK parameters were calculated using WIONLIN software. Brain-to-plasma ratio determination was carried out using mice dosed intravenously with 1 mg/kg of compounds, and blood and brain samples were collected at post 5 min and 60 min. Compound concentration was determined using the LCMS-MS method. All of the in-life studies were carried out under protocols approved by IACUC (Institutional animal care and use committee), following animal ethics guidelines of NITD, Emeryville, CA.

### Mouse Efficacy Studies

Efficacy of compounds to eradicate *T. b. brucei* infection in mice models of human African trypanosomiasis were carried out using protocols described elsewhere.^21,29^

#### Stage I Mouse Efficacy

Briefly, for hemolymphatic mice model (Stage I), NMRI mice were infected with bloodstream form of *T. b. brucei* STIB975 strain. Three days post infection, the mice were orally gavaged for 4 days with varying concentration of test compound. Each group of compound treatment had six mice that were monitored for blood parasitemia over a period of 31 days. At the end of 30 days, if the mice were clear of parasites, they were considered as cure. *In vivo* efficacy studies in mice were conducted at the Swiss Tropical and Public Health Institute (Basel) (License number 2813), according to the rules and regulations for the protection of animal rights (“Tierschutzverordnung”) of the Swiss “Bundesamt für Veterinärwesen”. They were approved by the veterinary office of Canton Basel-Stadt, Switzerland.

#### Stage II Mouse Efficacy

For meningoncephalic mice model (stage II), CD1 mice were infected with bioluminescent strain of *T. b. brucei* GVR35 and allowed for 21 days for the infection to reach the brain. On day 21, a group of six mice were treated orally with varying concentration of test compound for a period of 7 days. The mice were monitored weekly for parasitemia relapse using IVIS imaging system for bioluminescence. The mice were considered cured at the end of 90 days post infection, if no bioluminescence was detected. All animal procedures were undertaken in adherence to experimental guidelines and procedures approved by The Home Office of the UK government. All work was covered by Home Office Project Licence PPL60/4442 entitled “Molecular Genetics of Trypanosomes and Leishmania”. All animal protocols received approval from the University of York and University of Glasgow Ethics Committees.

### Determination of Microsomal Stability, Permeability, Plasma Protein Binding, and Brain Tissue Binding

Intrinsic metabolic clearance for compounds was determined in mouse, rat, and human liver microsomes using the compound-depletion method and LCMS-MS quantification.^38^ Permeability for compounds was assessed using MDR1-MDCK cell line. The extraction ratio was calculated using A–B and B–A permeability as described elsewhere.^39^ Mice plasma protein binding was determined using mouse blood and brain tissue binding was determined using rat brain tissue homogenates using rapid equilibrium dialysis approach.^40^

### Solubility Assay

Equilibrium solubility was determined using a miniaturized shake flask approach.^41^ Aliquots of 10 mM DMSO compound solution were dispensed in triplicate in 96-well plates. The DMSO was removed using a GeneVac HT4X evaporator for approximately 1.5 h. Media (pH 6.8 phosphate buffer) was added to each well to achieve a target concentration of 0.75–1 mM. The plate was sealed and shaken for a minimum of 16 h, then centrifuged for phase separation. The supernatant was centrifuged a second time in a new plate. Finally, an aliquot of the second supernatant was transferred to another plate for further dilution and subsequent analysis. Quantification of solubility was performed using high-performance liquid chromatography and MS/MS using a standard calibration curve prepared from the original DMSO compound solution. Experimental variability was determined from different days and experimentalists, with a log standard deviation of 0.25.

## Supporting Information Available

Experimental procedures and ^1^H NMR spectra for all mentioned compounds (PDF)
Molecular formula strings (CSV)

## Notes

The cryo-EM map is available from the Electron Microscopy Data Band with accession code EMD-15025. The atomic model has been deposited in the Protein Data Bank with the accession code PDB ID 7ZYJ.

## Abbreviations Used

3R: reduction refinement, replacement
ADME: absorption, distribution, metabolism, and excretion
AUC: area under the curve
BTB: brain tissue binding
Cl_int_: intrinsic clearance
CL_u_: unbound clearance
cmp: compound
CNS MPO: central nervous system multiparameter optimization
ER: efflux ratio
HAT: human African trypanosomiasis
hERG: human ether-a-go-go-related gene
HTS: high-throughput screening
IV: intravenous
IVIVC: *in vitro*–*in vivo* correlation
LM: liver microsomes
log BB: logarithm of blood-to-brain ratio
MDCK-MDR1: Madin Darby kindey cells with MDR1 gene
MNT: micronucleus test
mpk: mg/kg
PK: pharmacokinetics
PKPD: pharmacokinetics-pharmacodynamics
PPB: plasma protein binding
P-gp: P-glycoprotein
SAR: structure–activity relationship
SF: scaling factor
*T.b.b*.: Trypanosoma brucei brucei
WHO: World Health Organization
