# Supplementary material for: Discovery of Novel Quinoline-Based Proteasome Inhibitors for Human African Trypanosomiasis (HAT)
Source: J Med Chem. 2022 Aug 22;65(17):11776–87. doi: 10.1021/acs.jmedchem.2c00791 (PMC9469205; doi:10.1021/acs.jmedchem.2c00791)
Supplement: Supplementary file 1 — jm2c00791_si_001.pdf [file jm2c00791_si_001.pdf]

Supporting Information

Discovery of novel quinoline-based proteasome inhibitors for Human African Trypanosomiasis (HAT)

Dennis C. Koester[*,†], Vanessa M. Marx[†], Sarah Williams[†], Jan Jiricek[†], Maxime Dauphinais[†], Olivier René[†], Sarah L. Miller[†], Lei Zhang[†], Debjani Patra[‡], Yen-Liang Chen[※], Harry Cheung[※], Jonathan Gable[※], Suresh B. Lakshminarayana[×], Colin Osborne[÷], Jean-Rene Galarneau[˜], Upendra Kulkarni[±], Wendy Richmond[°], Angela Bretz[°], Linda Xiao[′], Frantisek Supek[<], Christian Wiesmann[>], Srinivas Honnappa[>], Celine Be[>], Pascal Mäser[ᴸ], Marcel Kaiser[ᴸ], Ryan Ritchie[=], Michael P. Barrett[=], Thierry T. Diagana[‡], Christopher Sarko[†], Srinivasa P. S. Rao[‡]

[†]Global Discovery Chemistry, Novartis Institutes for Biomedical Research, Emeryville, California, 94608, United States; [‡]Novartis Institutes for Tropical Diseases, Emeryville, California, 94608, United States; [※] Lead Discovery, Novartis Institutes for Tropical Diseases, Emeryville, California, 94608, United States; [×] Pharmacokinetic Sciences, Novartis Institutes for Tropical Diseases, Emeryville, California, 94608, United States; [÷] Pharmacokinetic Sciences, Pharmacology and Comparative Medicine, Novartis Institutes for Tropical Diseases, Emeryville, California, 94608, United States; [˜] Preclinical Safety, Novartis Institutes for Biomedical Research, Cambridge, Massachusetts, 02139, United States; [±] Chemical and Pharmaceutical Profiling, Novartis Institutes for Biomedical Research, Cambridge, Massachusetts, 02139, United States; [°]Global Discovery Chemistry, Novartis Institutes for Biomedical Research, San Diego, California, 92121, United States; [′] Pharmacology, Novartis Institutes for Tropical Diseases, Emeryville, California, 94608, United States; [<] Novartis Institutes for Biomedical Research, San Diego, California, 92121, United States; [>] Novartis Institutes for Biomedical Research, Basel, Switzerland; [ᴸ] Swiss Tropical and Public Health Institute, Kreuzstrasse 2, 4123 Allschwil, Switzerland; University of Basel, CH 4000 Basel, Switzerland; [=] University of Glasgow, University Place, Glasgow G12 8TA, UK

Email: dennis.c.koester@gmail.com

Table of Content

1) **Synthesis and Characterization of Final Compounds and Intermediates**
2) **Potency and clearance of additional compounds**
3) **Predicted and measured K_p and $K_{p,uu}$**

1) Synthesis and Characterization of Final Compounds and Intermediates

***N*-Cyclopentyl-6-methyl-4-(phenylamino)quinoline-2-carboxamide** (Compound **2**)

Scheme S1. Synthesis of compound **2**.

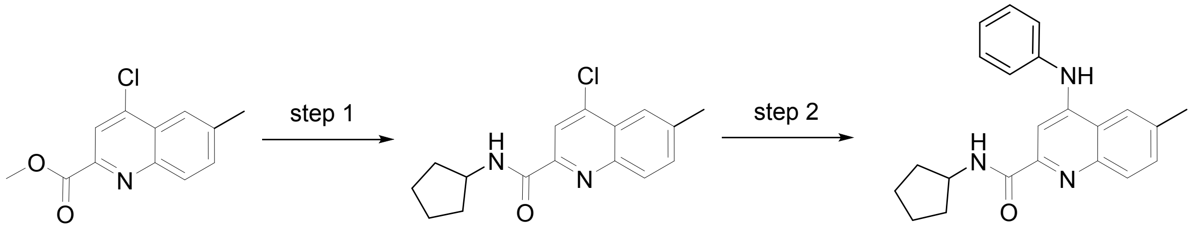

<u>Step 1:</u> **4-chloro-*N*-cyclopentyl-6-methylquinoline-2-carboxamide**

Cyclopentanamine (20 mg, 0.23 mmol) and lithium bis(trimethylsilyl)amide (233 µl, 0.23 mmol) were added to a solution of methyl 4-chloro-6-methylquinoline-2-carboxylate (50 mg, 0.21 mmol) in dioxane (2.1 mL). The reaction was stirred at room temperature for 5 min. The reaction was quenched with saturated aqueous ammonium chloride solution and extracted with ethyl acetate. The organic layers were combined, dried over sodium sulfate, filtered and concentrated. The crude material was used in the next steps without further purification. LCMS (ESI): *m*/*z* = 289.1 $[M+H]^+$

<u>Step 2:</u> ***N*-Cyclopentyl-6-methyl-4-(phenylamino)quinoline-2-carboxamide**

In a microwave vial 4-chloro-*N*-cyclopentyl-6-methylquinoline-2-carboxamide (61 mg, 0.21 mmol) was dissolved in methanol (1.0 mL). Aniline (0.039 mL, 0.43 mmol) and *p*-toluenesulfonic acid monohydrate (1.0 mg, 5.3 µmol) were added to the solution and the vial was sealed and placed in the microwave. The reaction was heated to 125 °C for 40 min. The reaction mixture was filtered through a 0.45 µm polytetrafluoroethylene syringe-tip filter and the product was purified by reverse-phase HPLC using 0.1% trifluoroacetic acid water and acetonitrile to give *N*-cyclopentyl-6-methyl-4-(phenylamino)quinoline-2-carboxamide (20 mg, 21%) as a pale yellow solid. LCMS (ESI): *m*/*z* = 346.4 $[M+H]^+$; ^{1}H NMR (500 MHz, DMSO-*d6*) δ 9.01 (s, 1H), 8.46 (s, 1H), 8.12 (d, *J* = 8.7 Hz, 1H), 7.85 (d, *J* = 8.6 Hz, 1H), 7.58 (t, *J* = 7.7 Hz, 2H), 7.51 (d, *J* = 7.8 Hz, 2H), 7.40 (d, *J* = 11.6 Hz, 2H), 4.24 - 4.20 (m, 1H), 2.58 (s, 3H), 2.02 - 1.88 (m, 2H), 1.70 (ddd, *J* = 6.9, 4.4, 2.3 Hz, 2H), 1.62 - 1.51 (m, 4H).

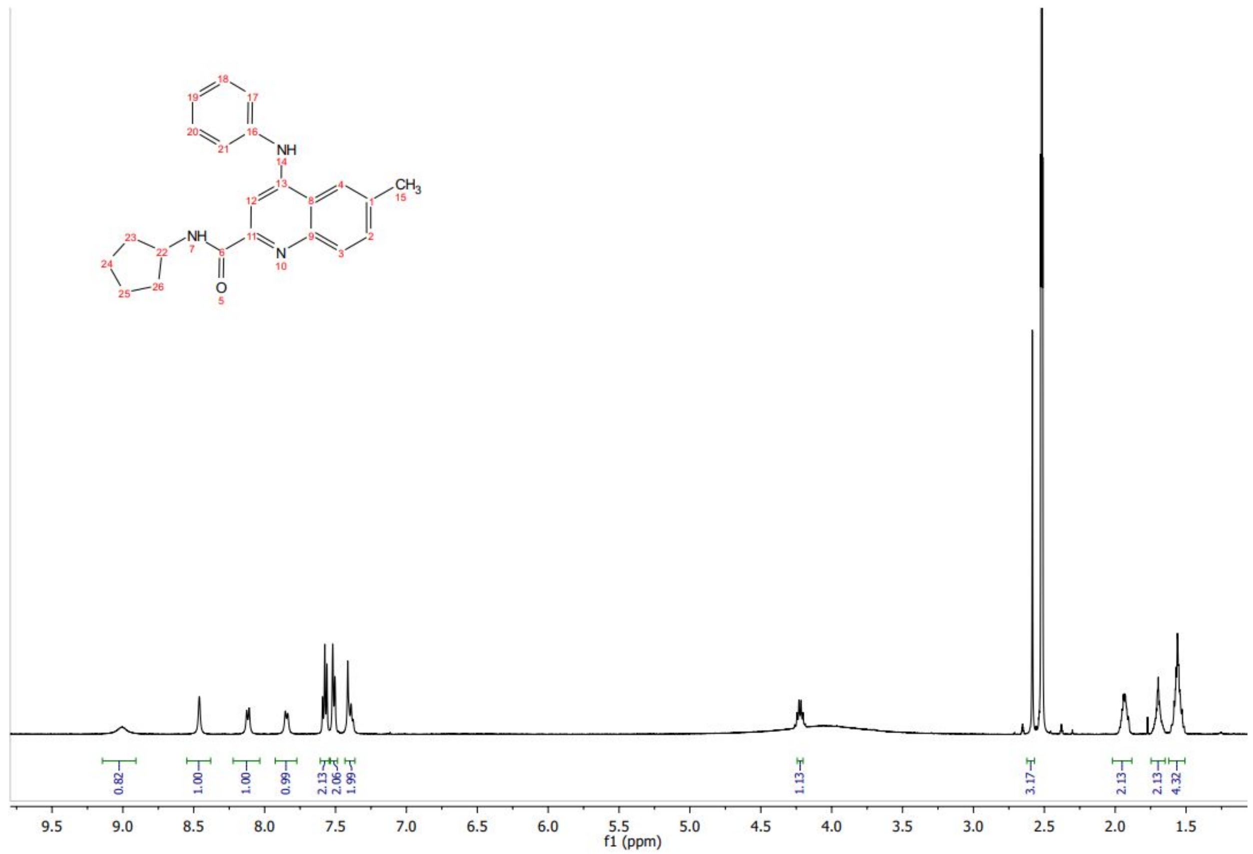

Example 1: (*R*)-*N*-(2,3-Dihydroxypropyl)-4-((2-fluorophenyl)amino)-6-methylquinoline-2-carboxamide (Compound **3**)

Scheme S2. Synthesis of compound **3**.

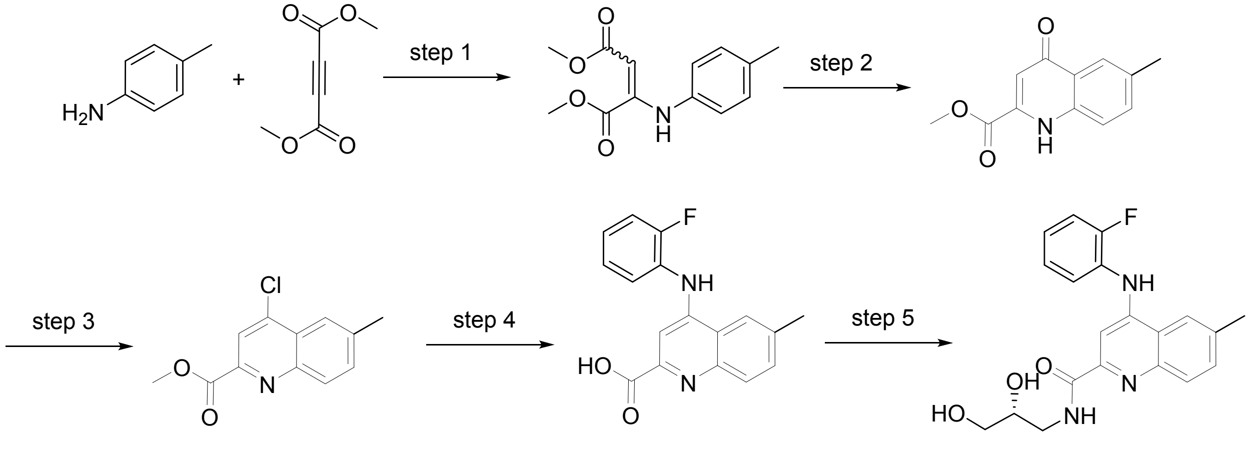

Step 1: **Dimethyl 2-(*p*-tolylamino)but-2-enedioate**

To a 0 °C solution of *p*-toluidine (4.0 g, 37 mmol) in methanol (75 mL) was added dimethyl but-2-ynedioate (9.2 mL, 75 mmol). The mixture was left to warm to room temperature and stirred for 15 min. Solvent was removed under vacuum and the crude material was taken to the next step without further purification.

Step 2: **Methyl 6-methyl-4-*oxo*-1,4-dihydroquinoline-2-carboxylate**

Dimethyl 2-(*p*-tolylamino)but-2-enedioate (9.3 g, 37 mmol) was dissolved in Eaton's reagent (50 mL, 37 mmol) and the mixture was stirred at 50 °C for 120 min. The reaction was then poured into a saturated aqueous sodium bicarbonate solution which caused precipitation. The solid was filtered off and washed with water and ether to afford the title compound.

Step 3: **Methyl 4-chloro-6-methylquinoline-2-carboxylate**

Methyl 6-methyl-4-*oxo*-1,4-dihydroquinoline-2-carboxylate (8.1 g, 37 mmol) was dissolved in phosphoryl chloride (20 mL, 215 mmol) and the mixture was stirred at 100 °C for 45 min. The reaction was then poured into a saturated aqueous sodium bicarbonate solution which caused precipitation. The solid was filtered off and washed with water to afford title compound.

Step 4: **4-((2-Fluorophenyl)amino)-6-methylquinoline-2-carboxylic acid**

Methyl 4-chloro-6-methylquinoline-2-carboxylate (2.0 g, 8.5 mmol), 2-fluoroaniline (0.86 mL, 8.9 mmol) and *p*-toluenesulfonic acid monohydrate (32 mg, 0.17 mmol) were dissolved in methanol (10 mL). The mixture was stirred under microwave irradiation at 125 °C for 45 min. Then, 2.0 M lithium hydroxide (8.5 mL, 17 mmol) was added and the resulting mixture was stirred at room temperature for 30 min. The reaction was concentrated to half volume and then acidified to pH 3 with 1 M aqueous hydrogen chloride. The precipitate was recovered to afford title compound as a yellow solid (2.5 g, 8.6 mmol, >99% yield).

Step 5: **(*R*)-*N*-(2,3-Dihydroxypropyl)-4-((2-fluorophenyl)amino)-6-methylquinoline-2-carboxamide**

To a solution of 4-((2-fluorophenyl)amino)-6-methylquinoline-2-carboxylic acid (60 mg, 0.20 mmol) and *N*,*N*-diisopropylethylamine (0.14 mL, 0.81 mmol) in dimethylformamide (1.0 mL) was added pivaloyl chloride (0.050 mL, 0.41 mmol) and the reaction was stirred at room temperature for 10 min. (*R*)-3-Aminopropane-1,2-diol (46 mg, 0.51 mmol) was then added and the reaction was stirred at room temperature for 1 hour. The reaction mixture was then partitioned between dichloromethane and water, the organic layer was isolated and concentrated and the product was purified by reverse-phase HPLC using 0.05% formic acid in water and acetonitrile to give (*R*)-*N*-(2,3-Dihydroxypropyl)-4-((2-fluorophenyl)amino)-6-methylquinoline-2-carboxamide (27 mg, 0.074 mmol, 36% yield) as a white solid. LCMS (ESI): *m*/*z* = 370.4 $[M+H]^+$; ^{1}H NMR (500 MHz, DMSO-*d*6) δ 9.05 (s, 1H), 8.66 (t, *J* = 6.0 Hz, 1H), 8.29 (s, 1H), 7.90 (d, *J* = 8.6 Hz, 1H), 7.65 (dd, *J* = 8.8, 1.8 Hz, 1H), 7.50 (td, *J* = 7.9, 1.9 Hz, 1H), 7.46 - 7.30 (m, 3H), 7.06 (d, *J* = 2.9 Hz, 1H), 4.97 (d, *J* = 4.8 Hz, 1H), 4.66 (t, *J* = 5.7 Hz, 1H), 3.68 - 3.57 (m, 1H), 3.51 (ddd, *J* = 13.4, 6.6, 4.6 Hz, 1H), 3.44 - 3.36 (m, 1H), 3.32 - 3.27 (m, 1H), 3.23 - 3.14 (m, 1H), 2.57 (s, 3H).

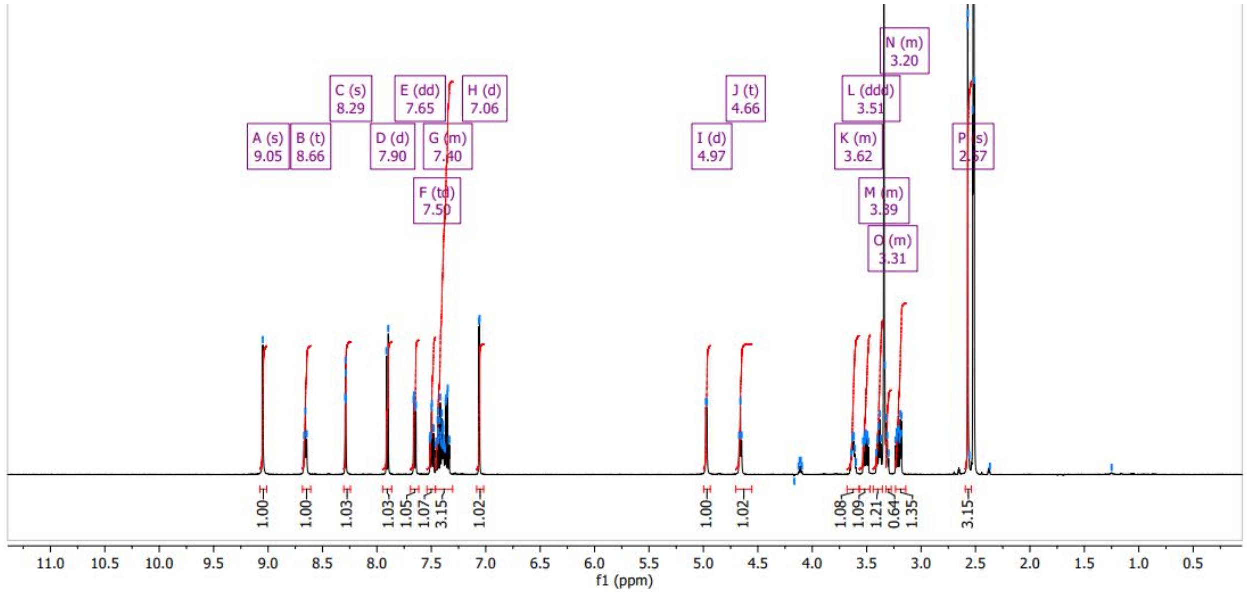

(6-methyl-4-(phenylamino)quinolin-2-yl)(pyrrolidin-1-yl)methanone (compound **1**)

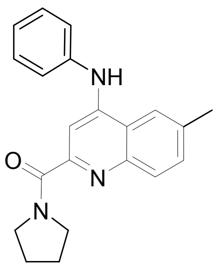

The title compound **1** was prepared according to the procedure of Example 1, using aniline and pyrrolidine as the starting material to give (6-methyl-4-(phenylamino)quinolin-2-yl)(pyrrolidin-1-yl)methanone as a pale yellow solid. LCMS (ESI): m/z = 332.1 [M+H]+; 1H NMR (300 MHz, Chloroform-d) δ 7.94 (d, J = 8.1 Hz, 1H), 7.70 (s, 1H), 7.55 (dd, J = 8.4, 1.8 Hz, 1H), 7.45 – 7.7.38 (m, 3H), 7.35 – 7.29 (m, 2H), 3.17 (t, J = 7.2 Hz, 1H), 6.64 (s, 1H), 3.75 (t, J = 6.8 Hz, 2H), 3.66 (t, J = 6.8 Hz, 2H), 2.58 (s, 3H), 2.0 – 1.85 (m, 4H).

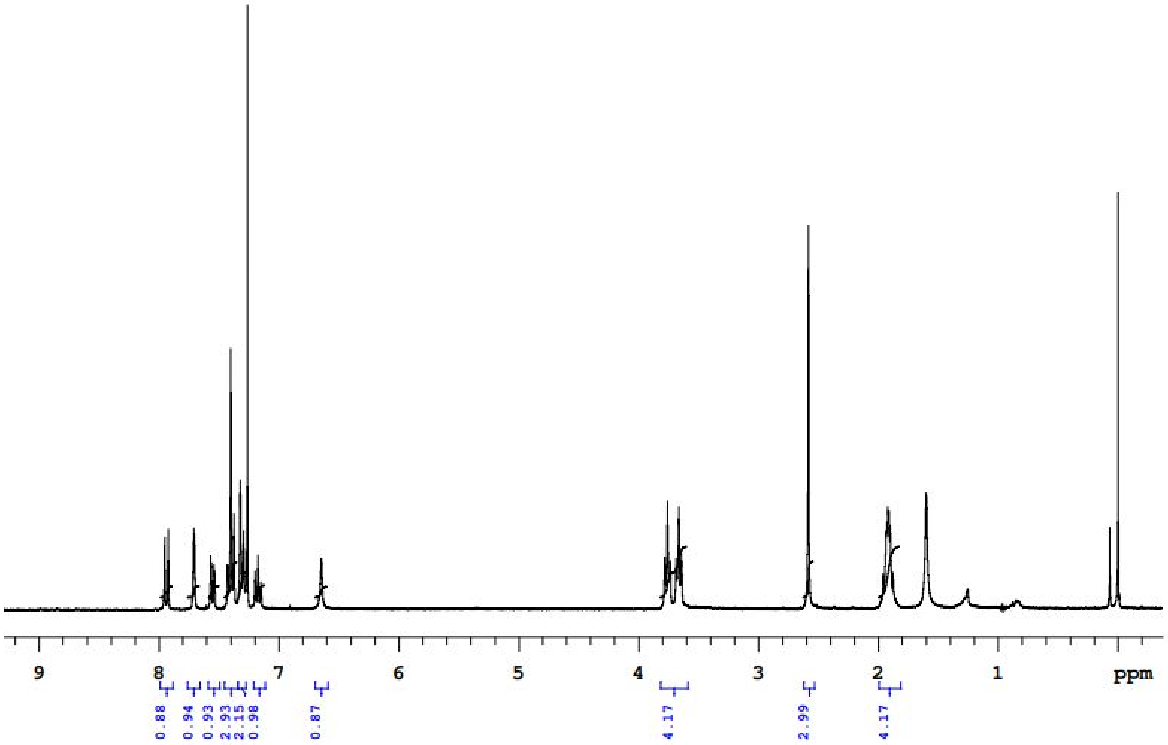

(*R*)-*N*-(2-fluoro-3-hydroxypropyl)-4-((2-fluorophenyl)amino)-6-methylquinoline-2-carboxamide (Compound **4**)

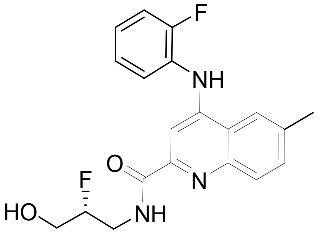

The title compound was prepared according to the procedure of Example 1, differing by the last peptide coupling step: To a solution of 4-((2-fluorophenyl)amino)-6-methylquinoline-2-carboxylic acid (2.0 g, 6.8 mmol) and (*R*)-3-amino-2-fluoropropan-1-ol (0.94 g, 10 mmol) in dimethylformamide (Volume: 12 mL) was added triethylamine (2.8 mL, 20 mmol) and propylphosphonic anhydride in ethyl acetate (6.0 mL, 13 mmol) and the resulting mixture was stirred at room temperature for 30 min. The reaction was diluted with ethyl acetate, washed with water and brine, dried over magnesium sulfate, filtered and concentrated. The resulting crude oil was purified by column chromatography (30-90% [9:1 ethyl acetate:methanol] in heptanes) to afford title compound as an offwhite solid (1.5 g, 4.1 mmol, 60% yield). LCMS (ESI): *m*/*z* = 372.3 $[M+H]^+$; ^{1}H NMR (500 MHz, Chloroform-*d*) δ 8.71 – 8.54 (m, 1H), 7.96 (d, *J* = 8.6 Hz, 1H), 7.84 (s, 1H), 7.75 (s, 1H), 7.60 (dd, *J* = 8.7, 1.8 Hz, 1H), 7.55 (td, *J* = 8.0, 1.7 Hz, 1H), 7.25 – 7.19 (m, 2H), 7.19 – 7.13 (m, 1H), 6.67 (s, 1H), 4.82 – 4.66 (m, 1H), 3.97 – 3.66 (m, 4H), 3.49 (s, 1H), 2.61 (d, *J* = 0.9 Hz, 3H).

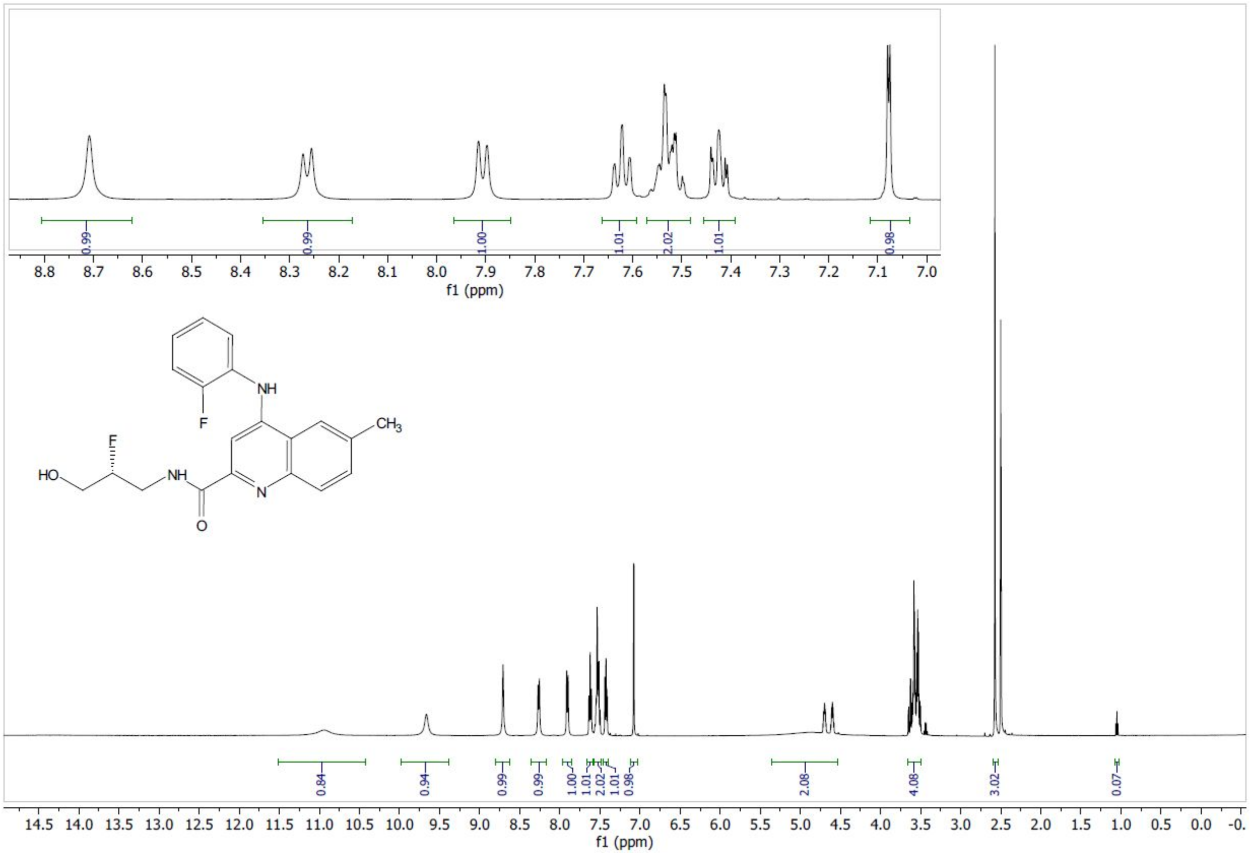

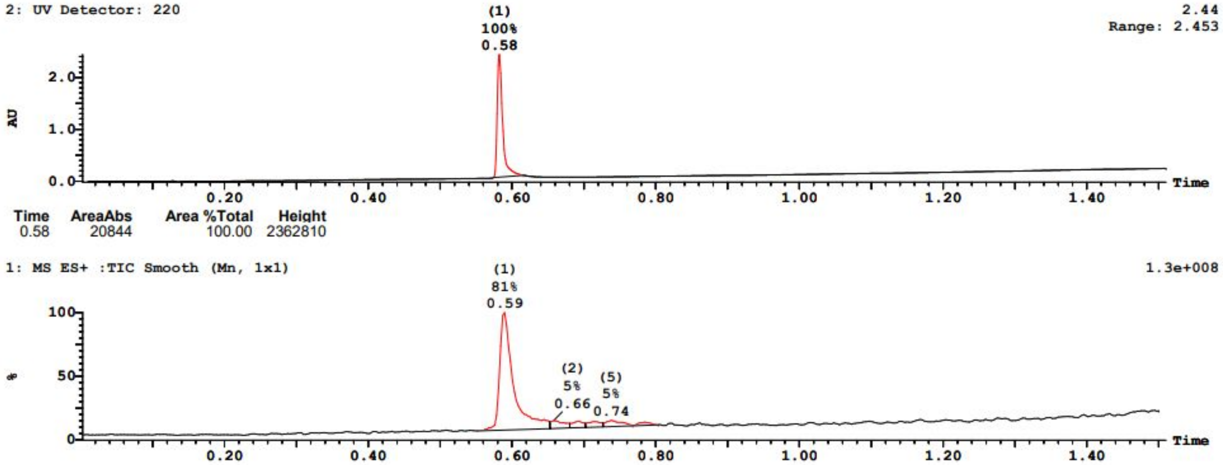

(*R*)-6-bromo-N-(2-fluoro-3-hydroxypropyl)-4-((2-fluorophenyl)amino)quinoline-2-carboxamide (Compound **5**)

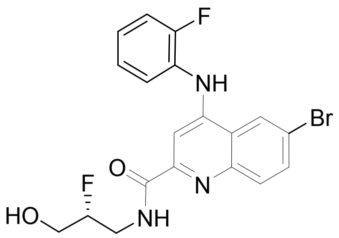

The title compound was prepared according to the procedure of Example 1, using 4-bromoaniline as the starting material, peptide coupling step: To a solution of 6-bromo-4-((2-fluorophenyl)amino)quinoline-2-carboxylic acid (30 mg, 0.083 mmol) and (*R*)-3-amino-2-fluoropropan-1-ol (11.60 mg, 0.125 mmol) in DMF (Volume: 1 mL) was added triethylamine (0.035 mL, 0.249 mmol) and propylphosphonic anhydride in DMF (0.097 mL, 0.166 mmol) and the resulting mixture was stirred at room temperature for 30 min. The mixture was filtered through syringe-tip filter and purified by reverse phase column chromatography to afford (*R*)-6-bromo-N-(2-fluoro-3-hydroxypropyl)-4-((2-fluorophenyl)amino)quinoline-2-carboxamide (21.1 mg, 0.048 mmol, 57.6 % yield) as a pale yellow solid. LCMS (ESI): *m*/*z* = 436.2 $[M+H]^+$; ^1H NMR(400 MHz, DMSO-*d6*) δ 9.27 (s, 1H), 8.91 (t, J = 6.1 Hz, 1H), 8.79 (d, J = 1.6 Hz, 1H), 7.94 (d, J = 1.2 Hz, 2H), 7.57 – 7.31 (m, 4H), 7.07 (d, J = 2.8 Hz, 1H), 4.67 (dt, J = 54.2, 7.3 Hz, 1H), 3.71 – 3.45 (m, 5H).

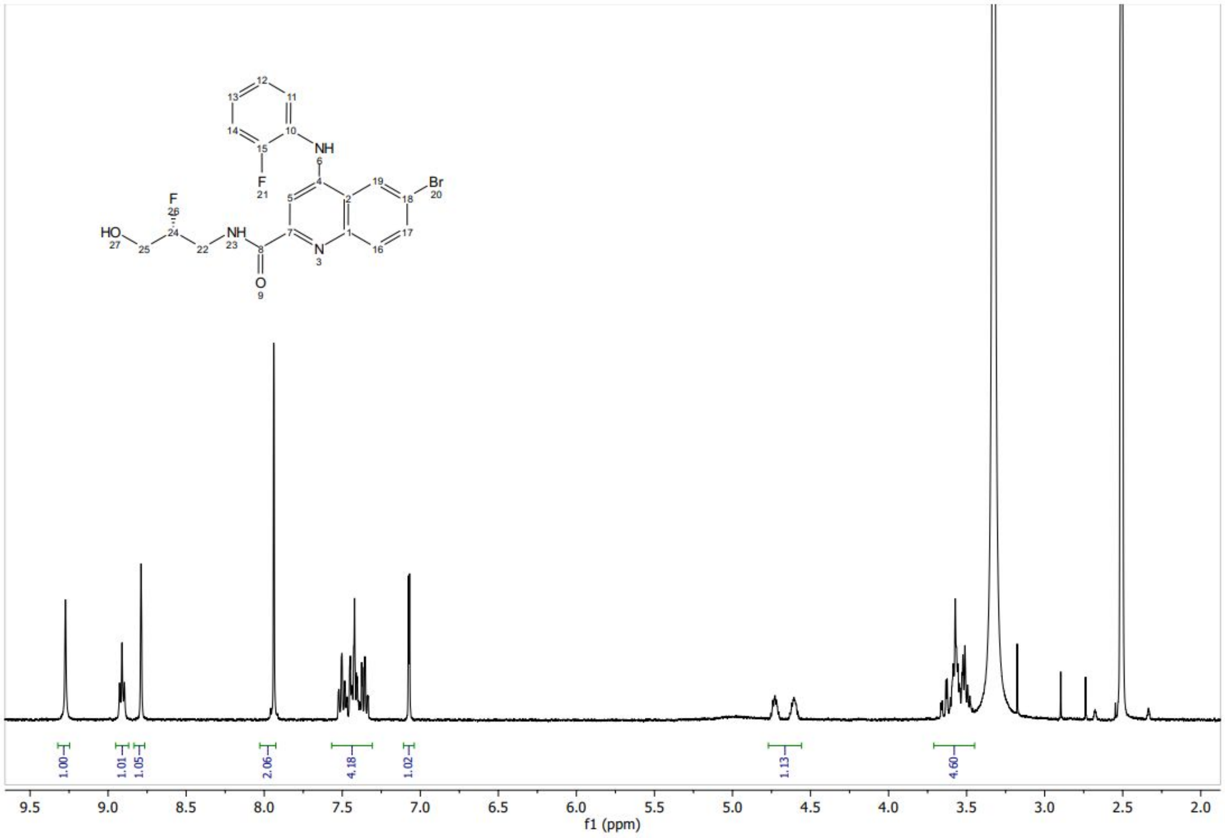

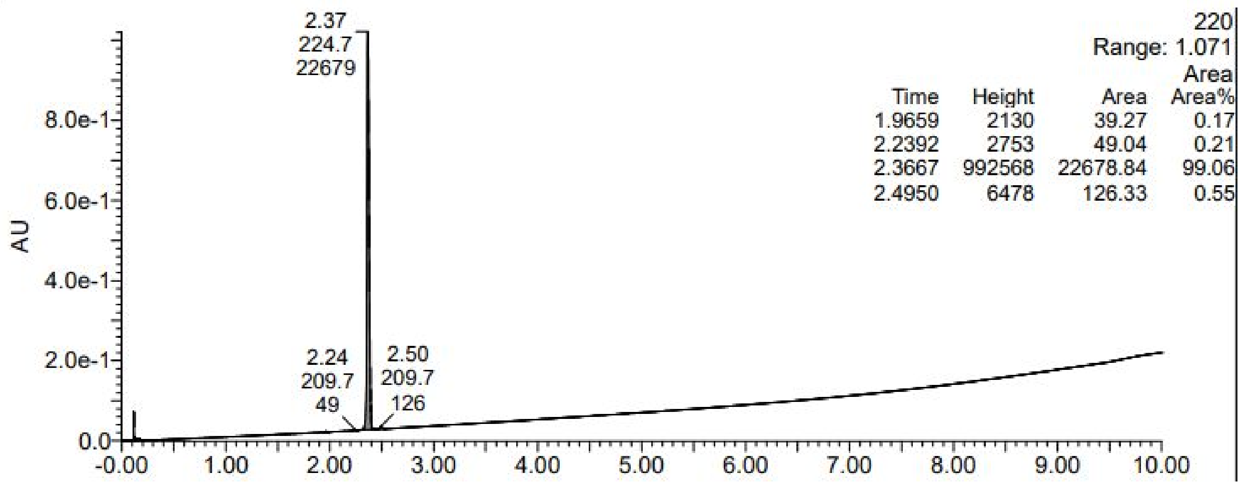

(*R*)-6-chloro-*N*-(2-fluoro-3-hydroxypropyl)-4-((2-fluorophenyl)amino)quinoline-2-carboxamide (Compound **6**)

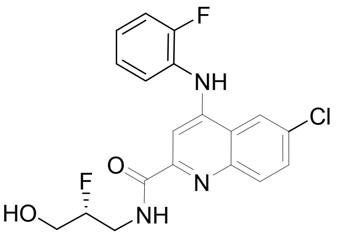

The title compound was prepared according to the procedure of Example 1, using 4-chloroaniline as the starting material and (*R*)-3-amino-2-fluoropropan-1-ol to give (*R*)-6-chloro-*N*-(2-fluoro-3-hydroxypropyl)-4-((2-fluorophenyl)amino)quinoline-2-carboxamide (18% yield) as a pale yellow solid. LCMS (ESI): *m*/*z* = 392.4 $[M+H]^+$; ^1H NMR (500 MHz, DMSO-*d6*) δ 9.27 (s, 1H), 8.93 (t, *J* = 6.2 Hz, 1H), 8.65 (d, *J* = 2.3 Hz, 1H), 8.03 (d, *J* = 9.0 Hz, 1H), 7.84 (dd, *J* = 9.0, 2.3 Hz, 1H), 7.58 - 7.32 (m, 4H), 7.08 (d, *J* = 2.8 Hz, 1H), 4.79 - 4.55 (m, 1H), 3.68 - 3.53 (m, 4H).

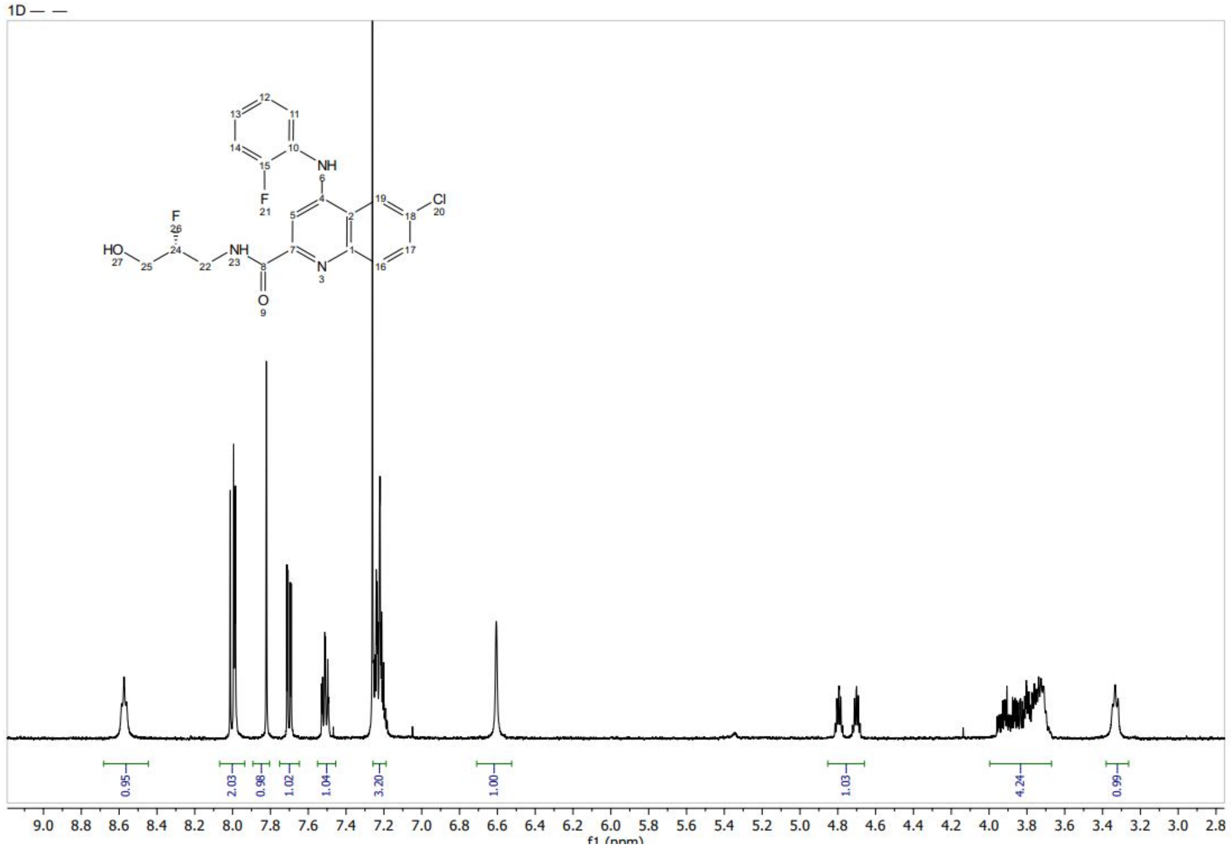

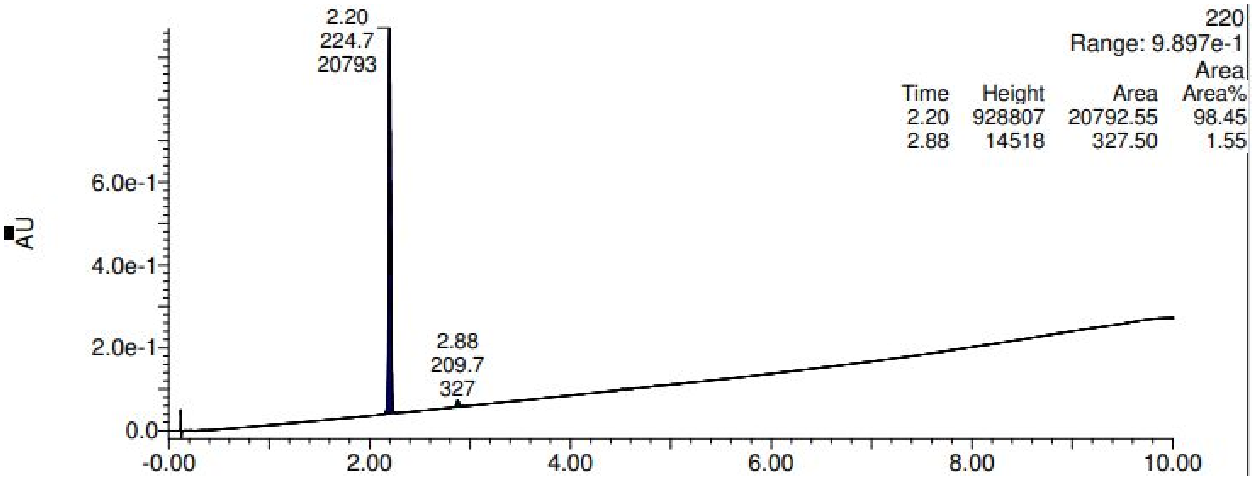

(*R*)-6-chloro-4-((2,3-difluorophenyl)amino)-N-(3-hydroxy-2-methoxypropyl)quinoline-2-carboxamide (Compound **7**)

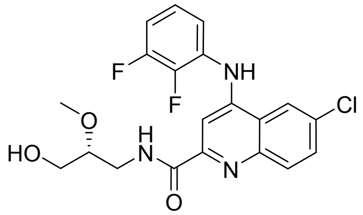

The title compound was prepared according to the procedure of Example 1, using 4-chloroaniline as the starting material and (*R*)-3-amino-2-methoxypropan-1-ol to give (*R*)-6-chloro-4-((2,3-difluorophenyl)amino)-N-(3-hydroxy-2-methoxypropyl)quinoline-2-carboxamide (53% yield) as an offwhite solid. LCMS (ESI): *m*/*z* = 422.3 $[M+H]^+$; ^1H NMR (500 MHz, Chloroform-*d*) δ 8.50 (s, 1H), 8.03 (dd, *J* = 9.0, 0.5 Hz, 1H), 7.98 (d, *J* = 2.2 Hz, 1H), 7.90 (s, 1H), 7.71 (dd, *J* = 9.0, 2.2 Hz, 1H), 7.31 – 7.26 (m, 1H), 7.18 – 7.12 (m, 1H), 7.06 – 6.97 (m, 1H), 6.58 (s, 1H), 3.82 (ddd, *J* = 14.3, 7.2, 4.0 Hz, 1H), 3.69 – 3.58 (m, 3H), 3.53 – 3.50 (m, 1H), 3.49 (s, 3H).

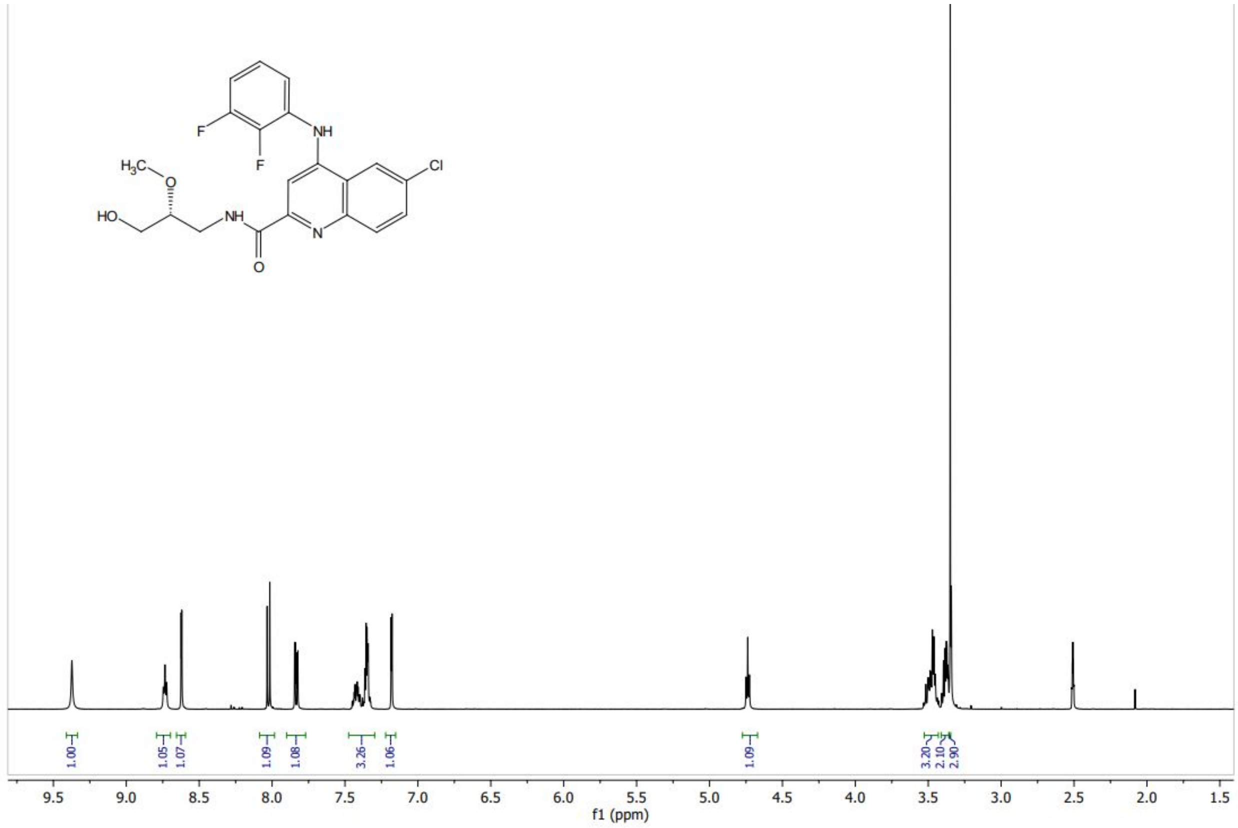

(*R*)-6-fluoro-4-((2-fluorophenyl)amino)-N-(3-hydroxy-2-methoxypropyl)quinoline-2-carboxamide (compound **16**)

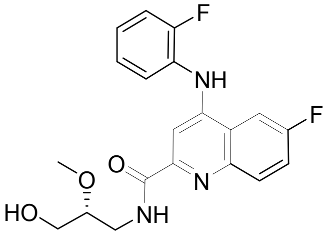

The title compound was prepared according to the procedure of Example 1, using 4-fluoroaniline as the starting material and (*R*)-3-amino-2-methoxypropan-1-ol to give (*R*)-6-fluoro-4-((2-fluorophenyl)amino)-N-(3-hydroxy-2-methoxypropyl)quinoline-2-carboxamide.

Peptide coupling step: To a solution of 6-fluoro-4-((2-fluorophenyl)amino)quinoline-2-carboxylic acid (0.572 g, 1.699 mmol) and (R)-3-amino-2-methoxypropan-1-ol (0.196 g, 1.869 mmol) in DMF (Volume: 4 ml) was added triethylamine (0.710 ml, 5.10 mmol) and propylphosphonic anhydride in ethyl acetate (1.983 ml, 3.40 mmol) and the resulting mixture was stirred at room temperature for 30 min. The reaction was diluted with ethyl acetate, washed with water and brine, dried over magnesium sulfate, filtered and concentrated. The resulting crude oil was purified by column chromatography (30-50% [3:1 AcOEt:EtOH] / heptanes). Combined fractions were concentrated and lyophilyzed to afford (*R*)-6-fluoro-4-((2-fluorophenyl)amino)-N-(3-hydroxy-2-methoxypropyl)quinoline-2-carboxamide (425 mg, 1.70 mmol, 64%) as an offwhite solid. LCMS (ESI): m/z = 388.3 [M+H]+;
^{1}H NMR (500 MHz, DMSO-d6) δ 9.10 (s, 1H), 8.70 (t, J = 5.8 Hz, 1H), 8.30 (dd, J = 10.8, 2.8 Hz, 1H), 8.07 (dd, J = 9.3, 5.6 Hz, 1H), 7.74 (ddd, J = 9.3, 8.1, 2.8 Hz, 1H), 7.54 – 7.32 (m, 4H), 7.08 (d, J = 2.8 Hz, 1H), 3.53 – 3.42 (m, 3H), 3.40 – 3.36 (m, 2H), 3.35 (s, 4H).

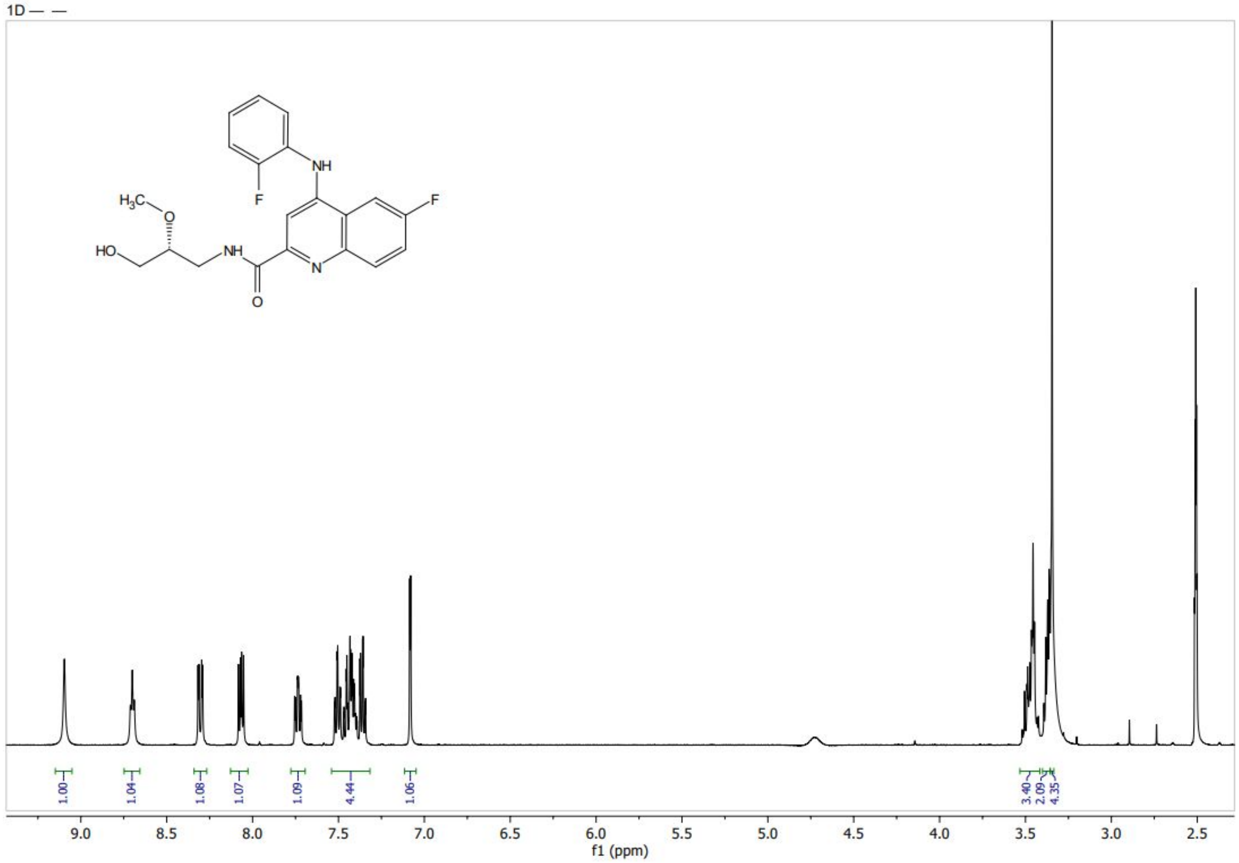
1D — —
NH
F
F
H3C
O
HO
NH
N
O
1.00
1.04
1.08
1.07
1.09
4.44
1.06
3.40
2.09
4.35
9.0
8.5
8.0
7.5
7.0
6.5
6.0
5.5
5.0
4.5
4.0
3.5
3.0
2.5
f1 (ppm)

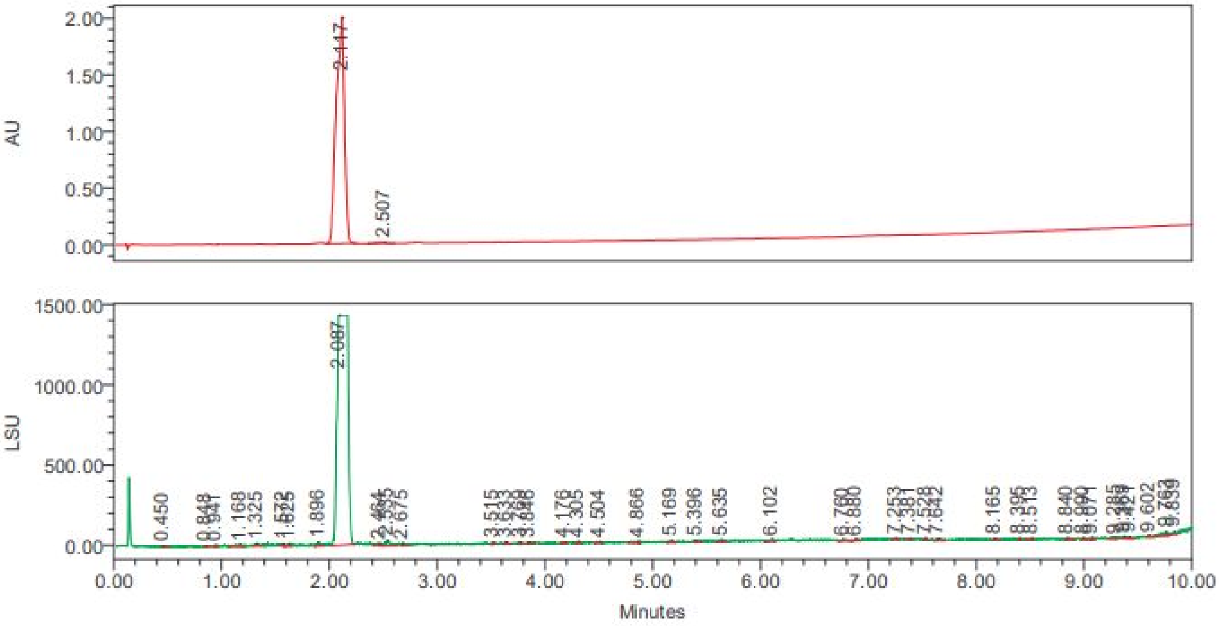

Peak Results
Channel: PDA Spectrum

	RT	% Area	Area	Height	Channel
1	2.117	99.45	10493195	1998665	PDA Spectrum
2	2.507	0.55	57956	10393	PDA Spectrum

N-(2-cyanoethyl)-4-((2-fluorophenyl)amino)-6-methylquinoline-2-carboxamide (compound **20**)

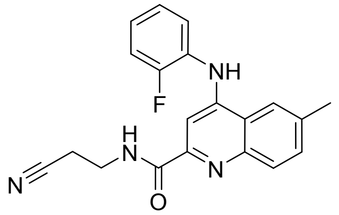

The title compound was prepared according to the procedure of Example 1, using 3-aminopropanenitrile to give N-(2-cyanoethyl)-4-((2-fluorophenyl)amino)-6-methylquinoline-2-carboxamide.

Peptide coupling step: To 4-((2-fluorophenyl)amino)-6-methylquinoline-2-carboxylic acid (20 mg, 0.060 mmol) in DCM (Volume: 1 mL) was added 2-(3H-[1,2,3]triazolo[4,5-b]pyridin-3-yl)-1,1,3,3-tetramethylisouronium hexafluorophosphate(V) [HATU] (28.6 mg, 0.075 mmol) and N-ethyl-N-isopropylpropan-2-amine (0.052 mL, 0.301 mmol). The reaction mixture was stirred at room temperature for 15 min before adding 3-aminopropanenitrile (6.32 mg, 0.090 mmol). The resulting mixture was stirred for additional 15 min. Additional equivalents for HATU, N-ethyl-N-isopropylpropan-2-amine and aminopropanenitrile were needed to achieve full conversion. The reaction mixture was concentrated to dryness. Redissolved in MeOH and filtered through a syringe-tip filter. The crude material was purified by reverse phase chromatography to afford N-(2-cyanoethyl)-4-((2-fluorophenyl)amino)-6-methylquinoline-2-carboxamide (6.6 mg, 0.019 mmol, 31 % yield) as an off-white solid. LCMS (ESI): m/z = 349.4 $[M+H]^+$; ^1H NMR (500 MHz, DMSO-d6) δ 9.10 - 9.02 (m, 2H), 8.29 (t, J = 1.6 Hz, 1H), 7.93 (d, J = 8.6 Hz, 1H), 7.67 (dd, J = 8.8, 1.8 Hz, 1H), 7.50 (td, J = 7.9, 1.8 Hz, 1H), 7.47 - 7.32 (m, 3H), 7.05 (d, J = 2.9 Hz, 1H), 3.55 (q, J = 6.5 Hz, 2H), 2.81 (t, J = 6.6 Hz, 2H), 2.58 (d, J = 0.9 Hz, 3H).

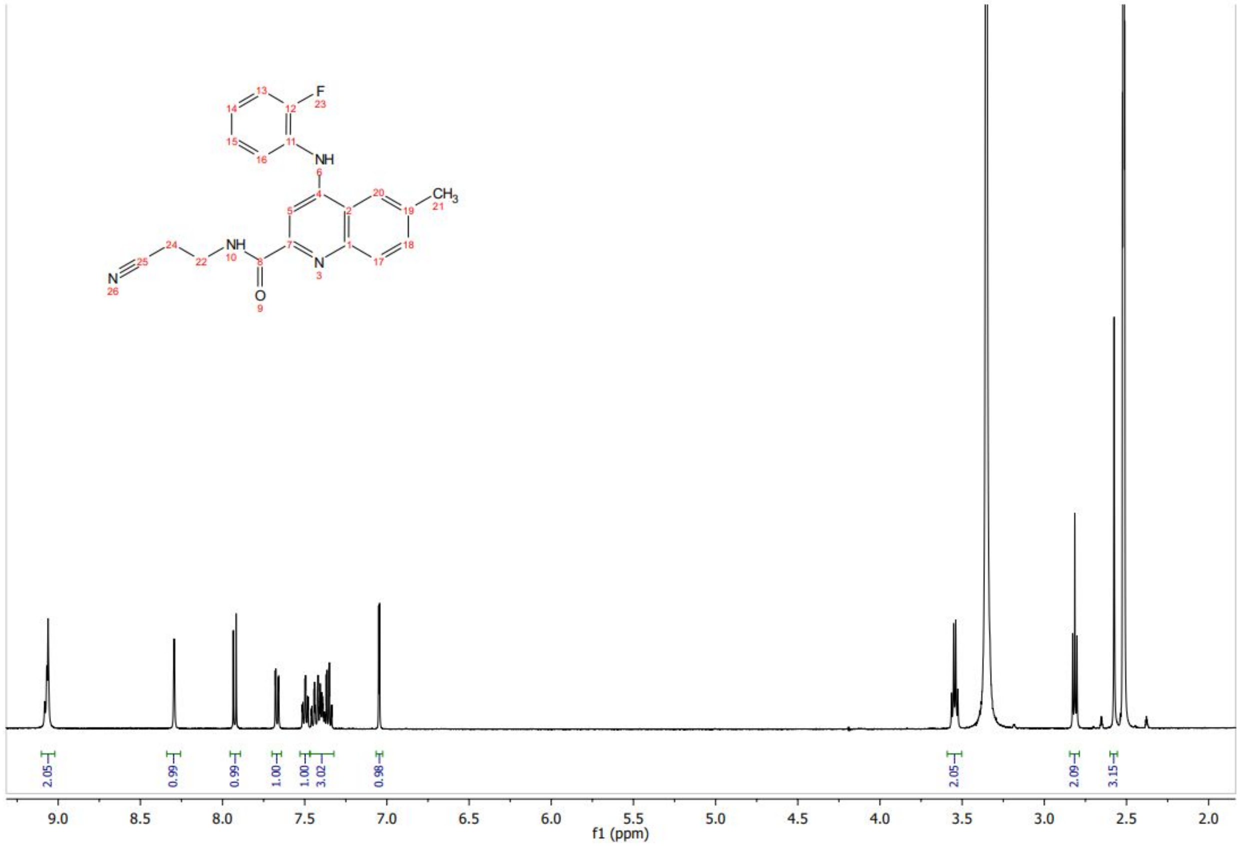

Example 2: *N*-Cyclopropyl-4-((2-fluorophenyl)amino)-6-methyl-1,7-naphthyridine-2-carboxamide (Compound **8**)

Scheme S3. Synthesis of compound **8**.

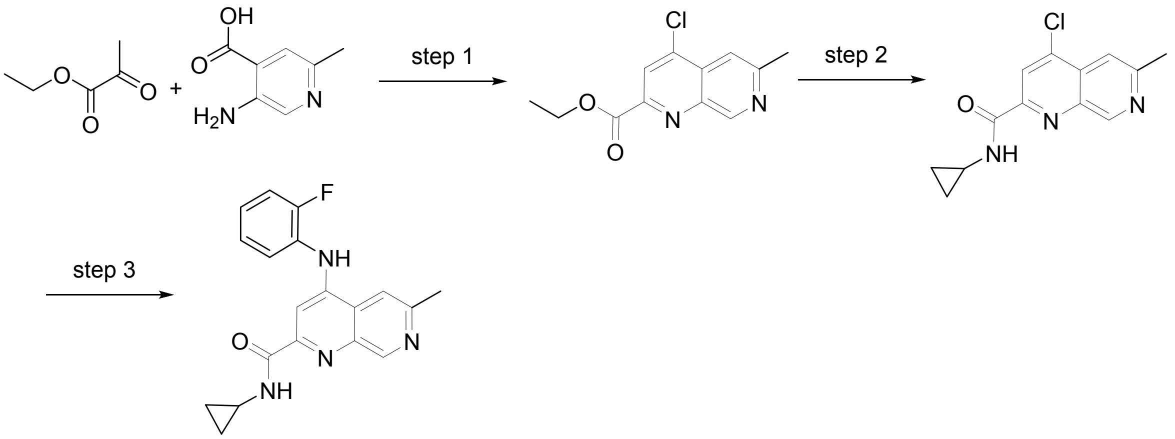

Step 1: **Ethyl 4-chloro-6-methyl-1,7-naphthyridine-2-carboxylate**

To a solution of 5-amino-2-methylisonicotinic acid (431 mg, 2.3 mmol) in phosphoryl chloride (2.5 mL, 27 mmol) was added ethyl pyruvate (0.77 mL, 6.9 mmol). The reaction mixture was heated in the microwave

for 3 hours at 110 °C. The reaction mixture was poured in a 1:1 mixture of water and saturated aqueous sodium bicarbonate solution. The aqueous layer was extracted with ethyl acetate. The organic layers were combined, dried over magnesium sulfate, filtered and concentrated to afford crude as a brown oil. The crude material was purified by column chromatography (10-50% ethyl acetate / heptanes) to afford ethyl 4-chloro-6-methyl-1,7-naphthyridine-2-carboxylate (282 mg, 49% yield) as a brown thick oil. LCMS (ESI): *m*/*z* = 251.2 $[M+H]^+$.

Step 2: **4-Chloro-*N*-cyclopropyl-6-methyl-1,7-naphthyridine-2-carboxamide**

Ethyl 4-chloro-6-methyl-1,7-naphthyridine-2-carboxylate (23 mg, 0.092 mmol) and cyclopropanamine (7.9 mg, 0.14 mmol) were dissolved in dioxane (100 µL). Lithium bis (trimethylsilyl)amide (183 µL, 0.18 mmol) was added and the mixture was stirred in the microwave at 125 °C for 20 min. The mixture was concentrated to dryness and used in the next step without further purification. LCMS (ESI): *m*/*z* = 262.2 $[M+H]^+$.

Step 3: ***N*-Cyclopropyl-4-((2-fluorophenyl)amino)-6-methyl-1,7-naphthyridine-2-carboxamide**

4-Chloro-*N*-cyclopropyl-6-methyl-1,7-naphthyridine-2-carboxamide (28 mg, 0.11 mmol), 2-fluoroaniline (18 mg, 0.16 mmol), sodium *tert*-butoxide (12 mg, 0.13 mmol) and BrettPhos Pd G3 (1.9 mg, 2.1 µmol) were suspended in dioxane (1 mL). The mixture was stirred at 100 °C for 2 hours in the microwave. The crude mixture was diluted with methanol and filtered through 0.45 µm polytetrafluoroethylene syringe-tip filter. The product was purified by reverse-phase HPLC using 0.1% trifluoroacetic acid water and acetonitrile to give *N*-cyclopropyl-4-((2-fluorophenyl)amino)-6-methyl-1,7-naphthyridine-2-carboxamide (2.2%) as a tan solid. LCMS (ESI): *m*/*z* = 337.4 $[M+H]^+$; ^1H NMR (500 MHz, Methanol-d4) δ 9.24 (s, 1H), 8.12 (s, 1H), 7.50 (t, J = 8.0 Hz, 1H), 7.43 – 7.37 (m, 1H), 7.34 (dd, J = 13.3, 5.3 Hz, 3H), 2.89 (tt, J = 7.6, 4.0 Hz, 1H), 2.77 (s, 3H), 0.93 – 0.81 (m, 2H), 0.78 – 0.67 (m, 2H).

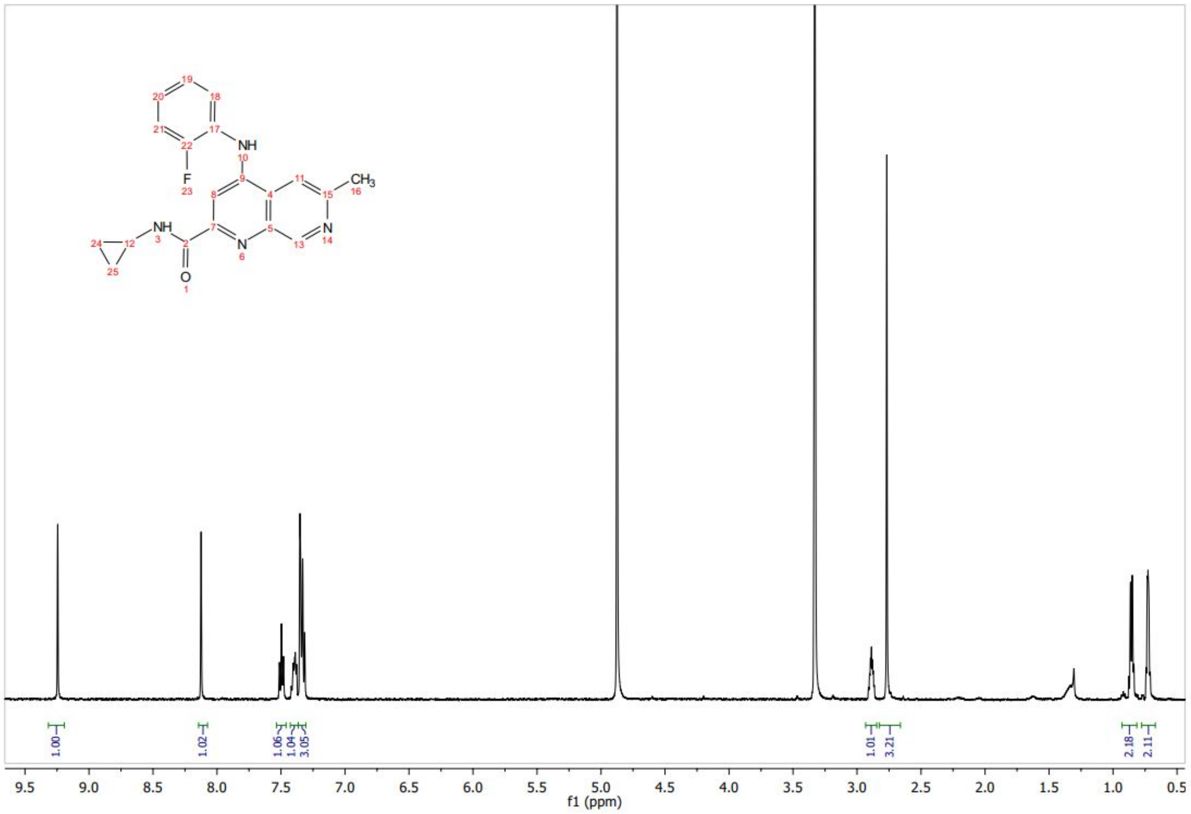

Example 3: *N*-(2-cyanoethyl)-4-((2-fluorophenyl)amino)-6-methyl-1,7-naphthyridine-2-carboxamide (compound **17**)

Scheme S4. Synthesis of compound **17**.

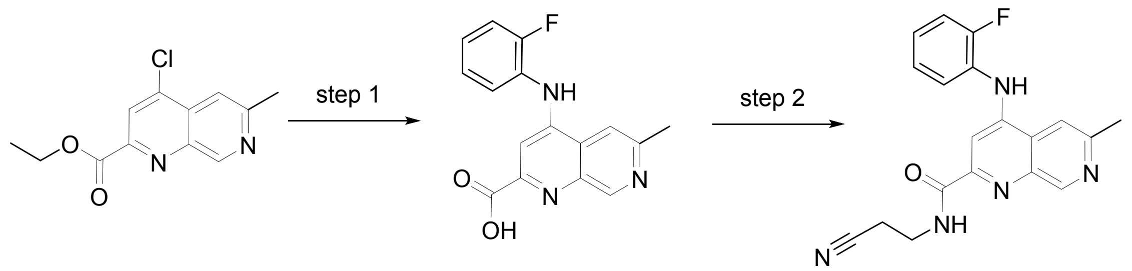

<u>Step 1:</u> **4-((2-fluorophenyl)amino)-6-methyl-1,7-naphthyridine-2-carboxylic acid**

Ethyl 4-chloro-6-methyl-1,7-naphthyridine-2-carboxylate (1.0 g, 4.0 mmol), 2-fluoroaniline (404 µL, 4.2 mmol) and *p*-toluenesulfonic acid monohydrate (15 mg, 0.080 mmol) were dissolved in methanol (4.0 mL). The mixture was stirred in a microwave at 125 °C for 15 min. Then, a 2 M aqueous lithium hydroxide solution (4.0 mL, 8.0 mmol) was added to the reaction, and the resulting mixture was stirred at room temperature for 1 hour. The reaction was acidified to pH 3. The precipitated solid was filtered off and recovered to afford 4-((2-fluorophenyl)amino)-6-methyl-1,7-naphthyridine-2-carboxylic acid (1.1 g, 3.7 mmol, 92 % yield) as a yellow solid.

<u>Step 2:</u> ***N*-(2-cyanoethyl)-4-((2-fluorophenyl)amino)-6-methyl-1,7-naphthyridine-2-carboxamide** (compound **17**)

Propylphosphonic anhydride in ethyl acetate (458 mg, 0.720 mmol) and triethylamine (0.201 mL, 1.440 mmol) were added to a solution of 4-((2-fluorophenyl)amino)-6-methyl-1,7-naphthyridine-2-carboxylic acid (107 mg, 0.360 mmol) in DCM (Volume: 2 mL). The reaction was stirred at room temperature for 15 min before 3-aminopropanenitrile (31.5 mg, 0.450 mmol) was added. The mixture was stirred for an additional 45 min at room temperature. Water and DCM were added to the reaction mixture. The phases were separated and the aqueous layer was extracted with DCM. The combined organic layers were dried over magnesium sulfate, filtered and concentrated. The resulting crude oil was purified by column chromatography (30-100% EtOAc in heptanes). Combined fractions were concentrated and lyophilyzed to afford N-(2-cyanoethyl)-4-((2-fluorophenyl)amino)-6-methyl-1,7-naphthyridine-2-carboxamide (35 mg, 0.099 mmol, 28%) as an offwhite solid. LCMS (ESI): m/z = 350.3 [M+H]+;
1H NMR (500 MHz, Methanol-d4) δ 9.28 (s, 1H), 8.14 (s, 1H), 7.50 (t, J = 8.0 Hz, 1H), 7.43 – 7.29 (m, 4H), 3.71 (t, J = 6.6 Hz, 2H), 2.82 (t, J = 6.7 Hz, 2H), 2.78 (s, 3H).

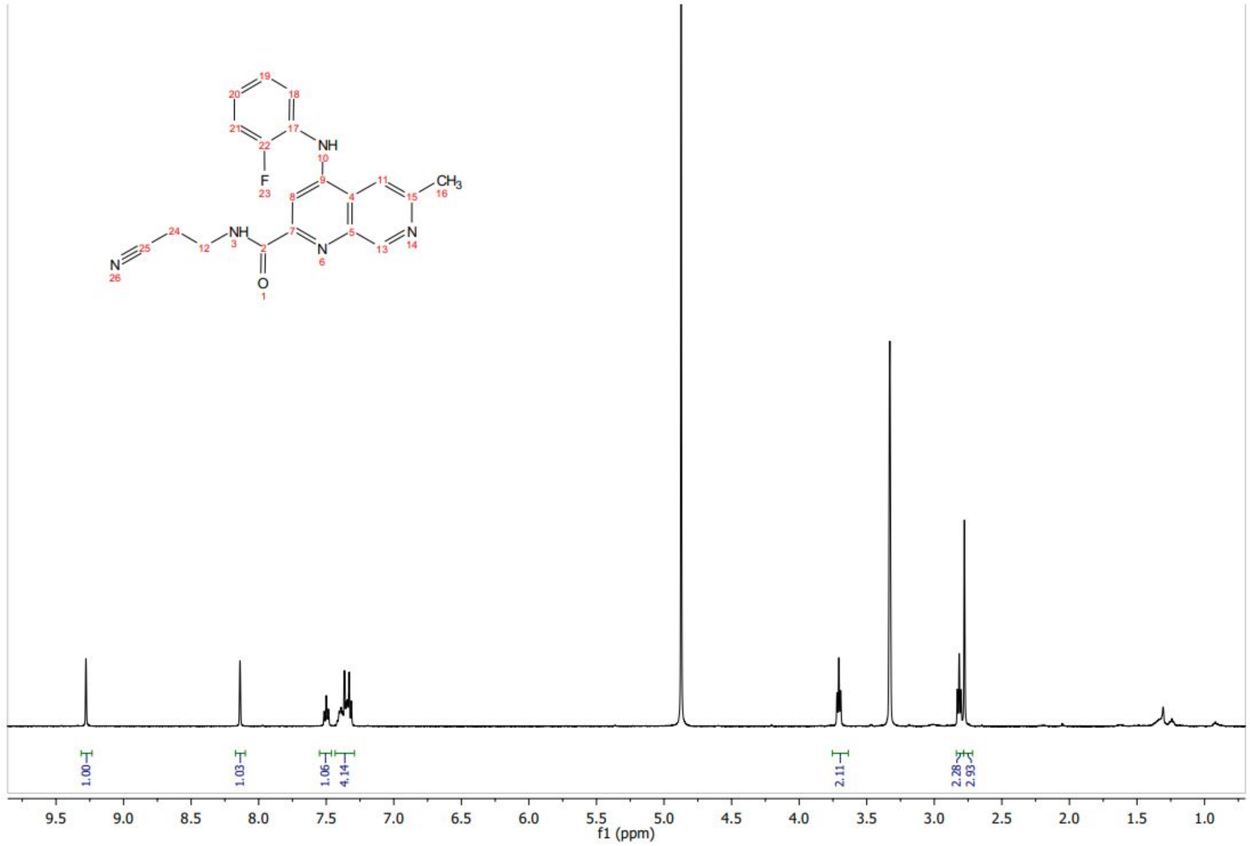

N-cyclopropyl-4-((2,6-difluorophenyl)amino)-6-methyl-1,7-naphthyridine-2-carboxamide (compound **9**)

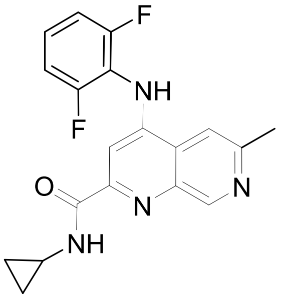

The title compound was prepared according to the procedure of Example 3, using 2,6-difluoroaniline as the starting material and cyclopropanamine to give N-cyclopropyl-4-((2,6-difluorophenyl)amino)-6-methyl-1,7-naphthyridine-2-carboxamide.

Peptide coupling step: Propylphosphonic anhydride in ethyl acetate (202 mg, 0.317 mmol) and triethylamine (0.088 mL, 0.634 mmol) were added to a solution of 4-((2,6-difluorophenyl)amino)-6-methyl-1,7-naphthyridine-2-carboxylic acid (50 mg, 0.159 mmol) in DCM (Volume: 1 mL). The reaction was stirred at room temperature for 15 min before cyclopropanamine (11.32 mg, 0.198 mmol) was added. The mixture was stirred for an additional 45 min at room temperature. Water and DCM were added to the reaction mixture. The phases were separated and the aqueous layer was extracted with DCM. The combined organic layers were dried over magnesium sulfate, filtered and concentrated. The resulting crude oil was purified by column chromatography (30-100% EtOAc in heptanes). Combined fractions were concentrated

and lyophilyzed to afford N-cyclopropyl-4-((2,6-difluorophenyl)amino)-6-methyl-1,7-naphthyridine-2-carboxamide (10 mg, 0.027 mmol, 17%) as an offwhite solid. LCMS (ESI): m/z = 355.3 [M+H]+; 1H NMR (500 MHz, Methanol-d4) δ 9.27 (s, 1H), 8.15 (s, 1H), 7.52 – 7.43 (m, 1H), 7.27 – 7.19 (m, 2H), 7.17 (d, J = 2.0 Hz, 1H), 2.94 – 2.86 (m, 1H), 2.78 (d, J = 1.7 Hz, 3H), 0.85 (t, J = 6.7 Hz, 2H), 0.73 (dd, J = 4.2, 2.2 Hz, 2H).

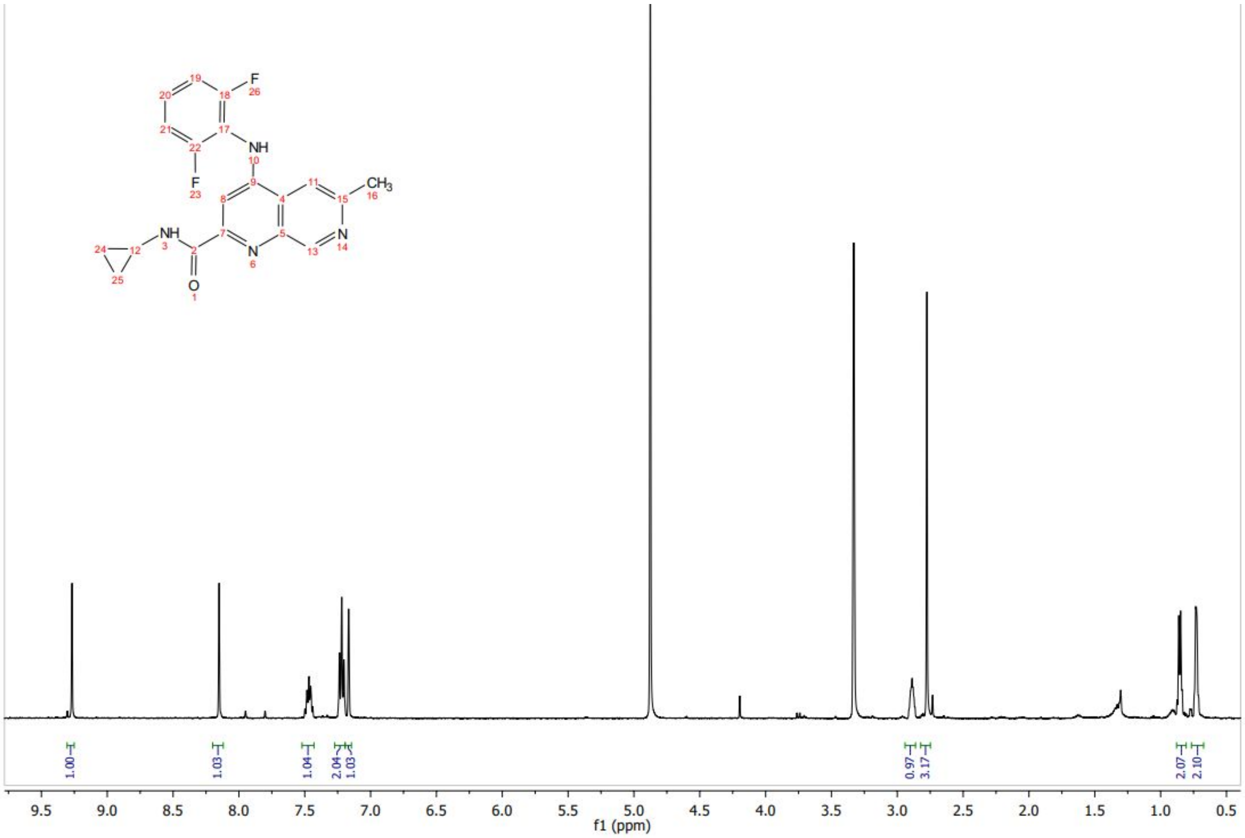

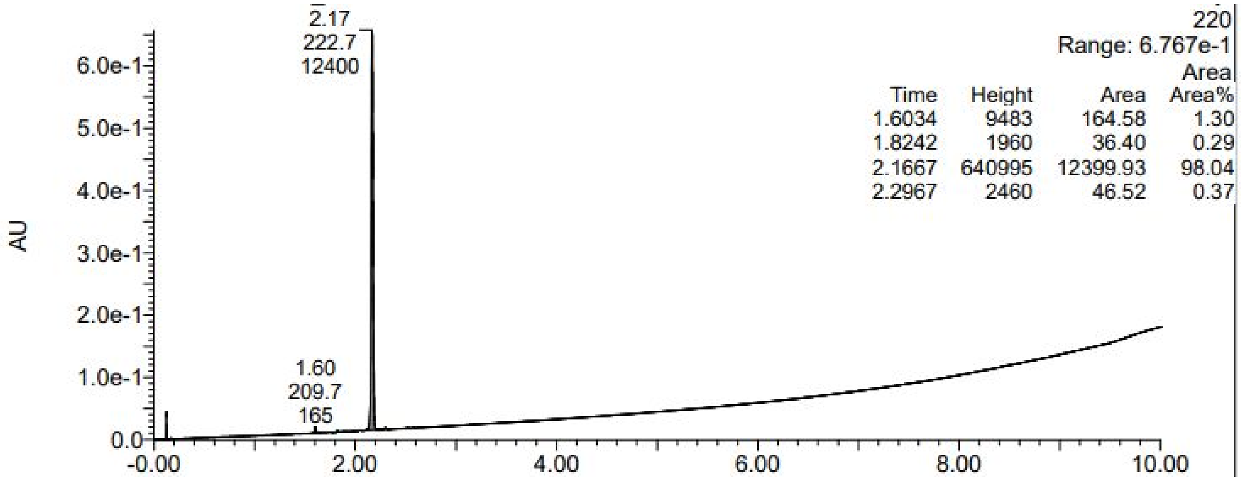

Example 4: *N*-((3*R*,4*S*)-3-fluoro-1-methylpiperidin-4-yl)-4-((2-fluorophenyl)amino)-6-methyl-1,7-naphthyridine-2-carboxamide (compound **10**)

Scheme S5. Synthesis of compound **10**.

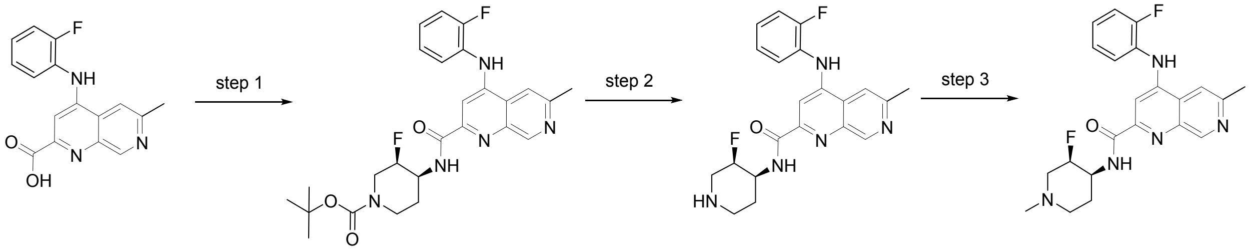

Step 1: ***tert*-Butyl (3*R*,4*S*)-3-fluoro-4-(4-((2-fluorophenyl)amino)-6-methyl-1,7-naphthyridine-2-carboxamido)piperidine-1-carboxylate**

To a solution of 4-((2-fluorophenyl)amino)-6-methylquinoline-2-carboxylic acid (70 mg, 0.24 mmol) and *N*,*N*-diisopropylethylamine (0.12 mL, 0.71 mmol) in *N*,*N*-dimethylformamide (5 mL) was added 1-bis(dimethylamino)methylene]-1*H*-1,2,3-triazolo[4,5-*b*]pyridinium 3-oxide hexafluorophosphate (134 mg, 0.35 mmol) and the mixture was stirred at room temperature for 1 minute. *tert*-butyl (3*R*,4*S*)-4-amino-3-fluoropiperidine-1-carboxylate (103 mg, 0.47 mmol) was then added and the reaction was stirred at room temperature for 20 minutes. The reaction and concentrated under reduced pressure to afford the title compound which was used without purification. LCMS (ESI): m/z = 498 $[M+H]^+$.

Step 2: **4-((2-Fluorophenyl)amino)-*N*-((3*R*,4*S*)-3-fluoropiperidin-4-yl)-6-methyl-1,7-naphthyridine-2-carboxamide**

Crude *tert*-butyl (3*R*,4*S*)-3-fluoro-4-(4-((2-fluorophenyl)amino)-6-methyl-1,7-naphthyridine-2-carboxamido)piperidine-1-carboxylate was dissolved in dichloromethane (3 mL) and trifluoroacetic acid (3 mL) was added at room temperature. The reaction was stirred for 30 minutes at room temperature. The resulting mixture was partitioned between dichloromethane and saturated aqueous potassium carbonate, the organic layer was isolated and concentrated to give the title compound (92 mg, 0.23 mmol, 98 % yield) which was used purification. LCMS (ESI): m/z = 398 $[M+H]^+$.

Step 3: ***N*-((3*R*,4*S*)-3-fluoro-1-methylpiperidin-4-yl)-4-((2-fluorophenyl)amino)-6-methyl-1,7-naphthyridine-2-carboxamide**

To a solution of 4-((2-fluorophenyl)amino)-*N*-((3*R*,4*S*)-3-fluoropiperidin-4-yl)-6-methyl-1,7-naphthyridine-2-carboxamide (94 mg, 0.24 mmol) in methanol (6.8 mL) was added formaldehyde (0.53 mL, 7.1 mmol) and acetic acid (0.081 mL, 1.4 mmol) and the reaction was stirred at room temperature for 5 minutes. Sodium triacetoxyborohydride (150 mg, 0.71 mmol) was then added and the reaction was stirred at room temperature for 30 minutes. The product was purified by reverse-phase HPLC (0.1% trifluoroacetic acid water and acetonitrile) to give the title compound (59.4 mg, 0.14 mmol, 59 % yield) as a white solid. LCMS (ESI): m/z = 412.4 $[M+H]^+$; ^1H NMR (500 MHz, DMSO-*d6*) δ 9.38 (s, 1H), 9.24 (s, 1H), 8.45 - 8.43 (m, 1H), 8.21 (s, 1H), 7.50 – 7.30 (m ,4H), 7.07 – 7.11 (m ,1H), 4.80 – 4.70 (m , 1H), 4.0 – 3.80 (m , 1H), 3.10 – 3.00 (m, 1H), 2.80 – 2.76 (m, 1H), 2.68 (s, 3H), 2.54 (s, 1H), 2.18 (s, 3H), 2.15 – 2.00 (m, 1H), 1.95 – 1.85 (m ,1H), 1.75 – 1.65 (m, 1H).

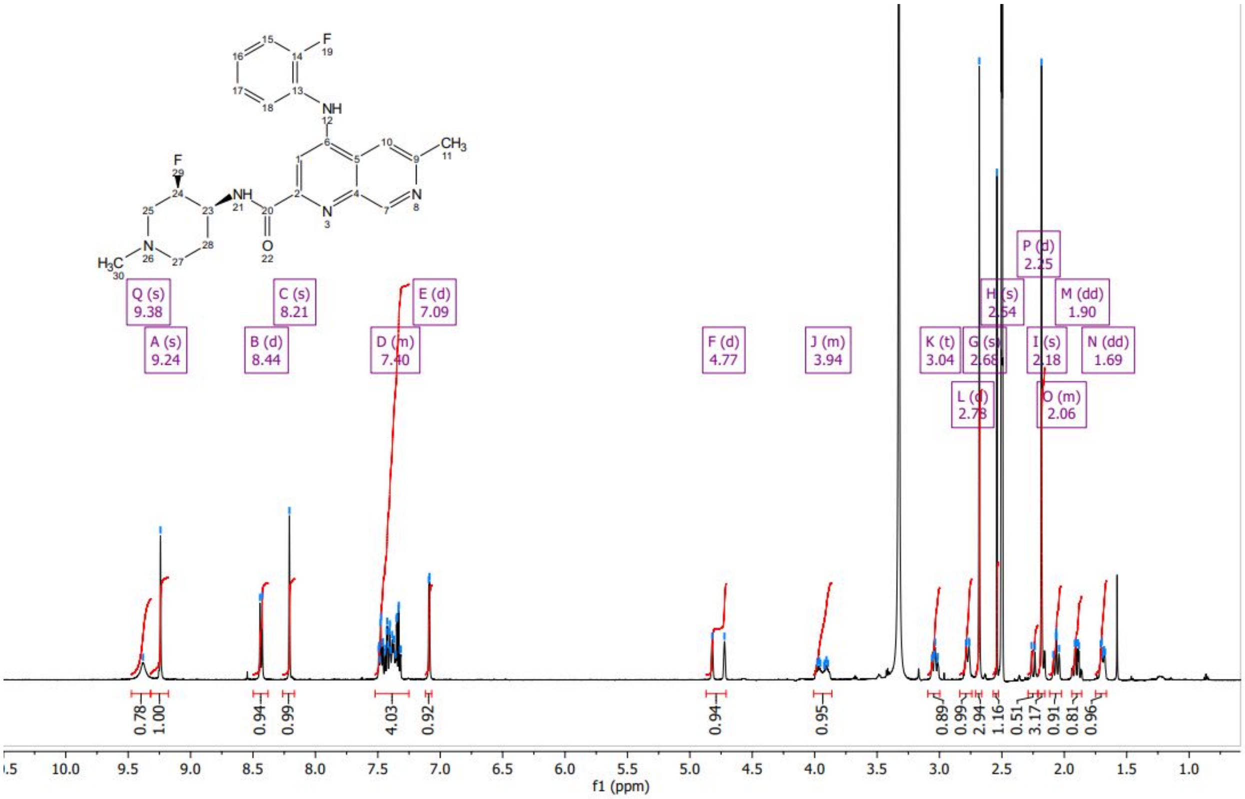

(R)-N-(2,3-dihydroxypropyl)-4-((2-fluorophenyl)amino)-6-methyl-1,7-naphthyridine-2-carboxamide (compound **11**)

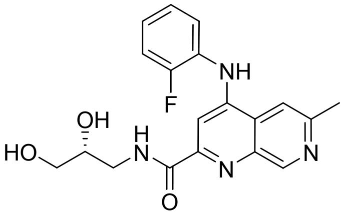

The title compound was prepared according to the procedure of Example 3, using 2-fluoroaniline as the starting material.

Peptide coupling step: A mixture of 4-((2-fluorophenyl)amino)-6-methyl-1,7-naphthyridine-2-carboxylic acid (25 mg, 0.084 mmol), 2-(3*H*-[1,2,3]triazolo[4,5-*b*]pyridin-3-yl)-1,1,3,3-tetramethylisouronium hexafluorophosphate(V) (38 mg, 0.10 mmol) and (*R*)-3-aminopropane-1,2-diol (9.1 mg, 0.10 mmol) was dissolved in dichloromethane (500 µL). *N*-ethyl-*N*-isopropylpropan-2-amine (44 µL, 0.25 mmol) was added and the reaction was stirred at room temperature for 30 min. The mixture was concentrated to dryness, re-dissolved in dimethyl sulfoxide and filtered through a 0.45 µm polytetrafluoroethylene syringe-tip filter. The product was purified by reverse-phase HPLC (0.1% formic acid water and acetonitrile) to afford (*R*)-N-(2,3-dihydroxypropyl)-4-((2-fluorophenyl)amino)-6-methyl-1,7-naphthyridine-2-carboxamide (13.2 mg, 0.031 mmol, 37 % yield) as a yellow solid.

LCMS (ESI): m/z = 371.4 $[M+H]^+$; ^1H NMR (500 MHz, DMSO) δ 9.32 (s, 1H), 9.24 (s, 1H), 8.70 – 8.67 (m, 1H), 8.22 (s, 1H), 7.51 – 7.36 (m, 4H), 7.14 (s, 1H), 4.97 (s, 1H), 4.68 – 4.65 (m, 1H), 3.63 – 3.59 (m, 1H), 3.53 – 3.46 (m, 1H), 3.41 – 3.37 (m, 1H), 3.32 – 3.28 (m, 1H), 3.25 – 3.17 (m, 1H), 2.69 (s, 3H).

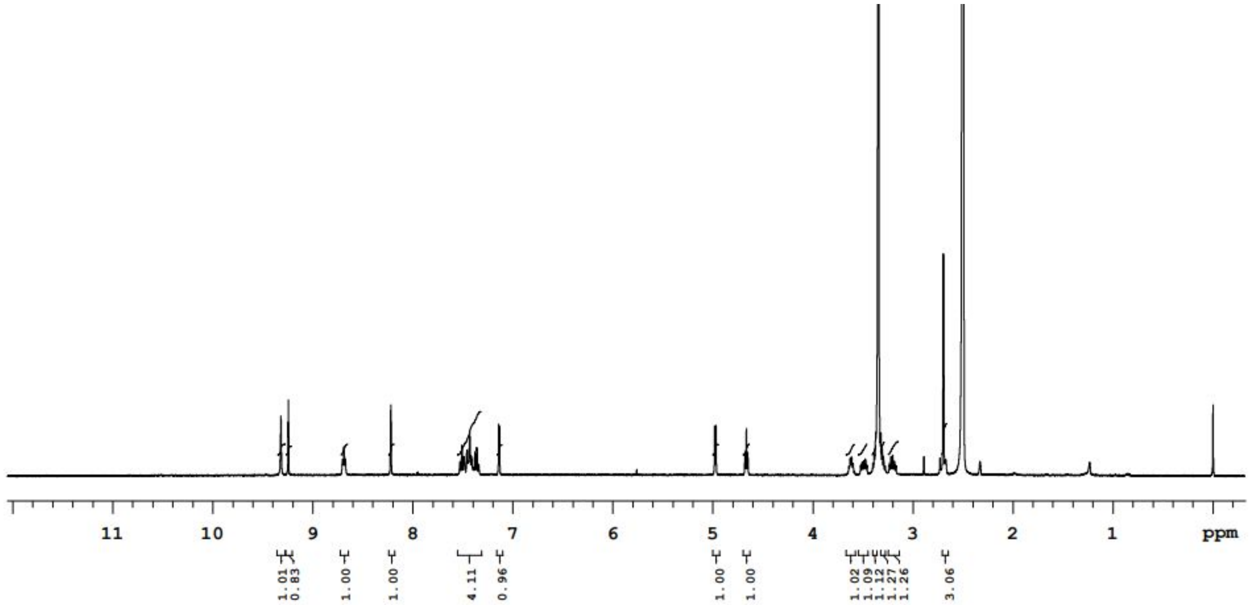

N-(2-cyano-2-methylpropyl)-4-((2-fluorophenyl)amino)-6-methyl-1,7-naphthyridine-2-carboxamide (compound **12**)

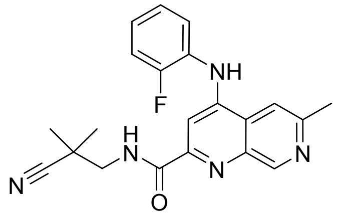

The title compound was prepared according to the procedure of Example 3, using 2-fluoroaniline as the starting material.

Peptide coupling step: A mixture of 4-((2-fluorophenyl)amino)-6-methyl-1,7-naphthyridine-2-carboxylic acid (25 mg, 0.084 mmol), 2-(3*H*-[1,2,3]triazolo[4,5-*b*]pyridin-3-yl)-1,1,3,3-tetramethylisouronium hexafluorophosphate(V) (38 mg, 0.10 mmol) and 3-amino-2,2-dimethylpropanenitrile (9.9 mg, 0.10 mmol) was dissolved in dichloromethane (500 µL). *N*-ethyl-*N*-isopropylpropan-2-amine (44 µL, 0.25 mmol) was added and the reaction was stirred at room temperature for 30 min. The mixture was concentrated to dryness, re-dissolved in dimethyl sulfoxide and filtered through a 0.45 µm polytetrafluoroethylene syringe-tip filter. The product was purified by reverse-phase HPLC (0.1% formic acid water and acetonitrile) to afford *N*-(2-cyano-2-methylpropyl)-4-((2-fluorophenyl)amino)-6-methyl-1,7-naphthyridine-2-carboxamide (13.4 mg, 0.031 mmol, 37 % yield) as a yellow solid.

LCMS (ESI): *m/z* = 378.4 $[M+H]^+$; ^1H NMR (500 MHz, DMSO) δ 9.35 (s, 1H), 9.30 (s, 1H), 9.06 (t, *J* = 6.8 Hz, 1H), 8.25 (d, *J* = 0.9 Hz, 1H), 7.53 (td, *J* = 8.0, 1.8 Hz, 1H), 7.50 – 7.41 (m, 2H), 7.38 (td, *J* = 7.5, 1.8 Hz, 1H), 7.15 (d, *J* = 2.9 Hz, 1H), 3.52 (d, *J* = 6.8 Hz, 2H), 2.72 (s, 3H), 1.34 (s, 6H).

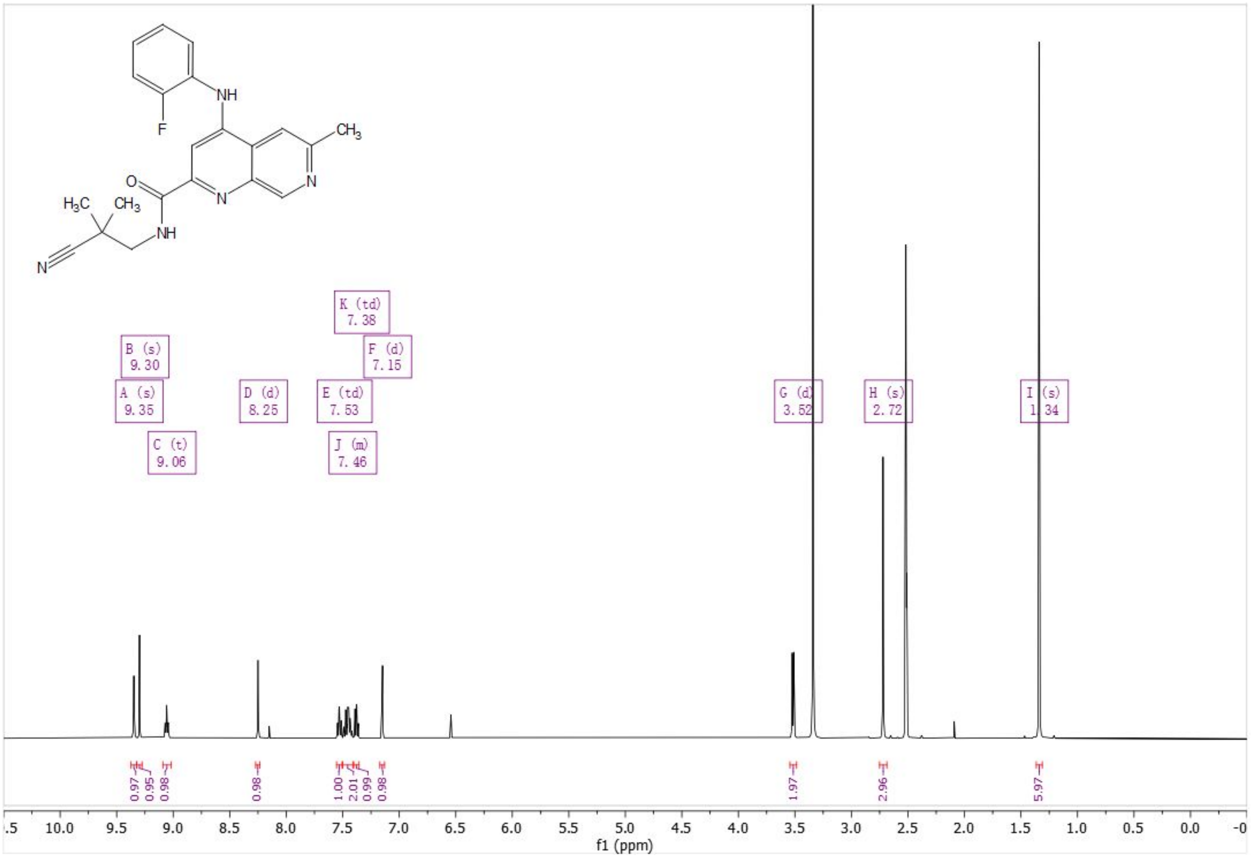

4-((2-Fluorophenyl)amino)-*N*-(2-methoxyethyl)-6-methyl-1,7-naphthyridine-2-carboxamide (compound **18**)

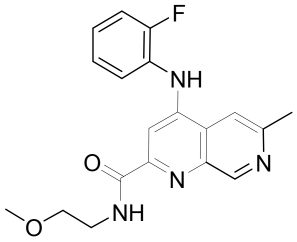

The title compound was prepared according to the procedure of Example 3, using 2-methoxyethan-1-amine, to give title compound (56% yield) as a brown solid. LCMS (ESI): m/z = 355.4 $[M+H]^+$; ^1H NMR (400 MHz, Chloroform-d) δ 9.38 (s, 1H), 8.50 - 8.40 (m, 1H), 7.95 (s, 1H), 7.59 (s, 1H), 7.58 – 7.50 (m ,1H), 7.26 – 7.22 (m, 3H). 6.64 (s, 1H), 3.70 – 3.68 (m, 2H), 3.62 – 3.59 (m, 2H), 3.43 (3H), 2.79 (s, 3H).

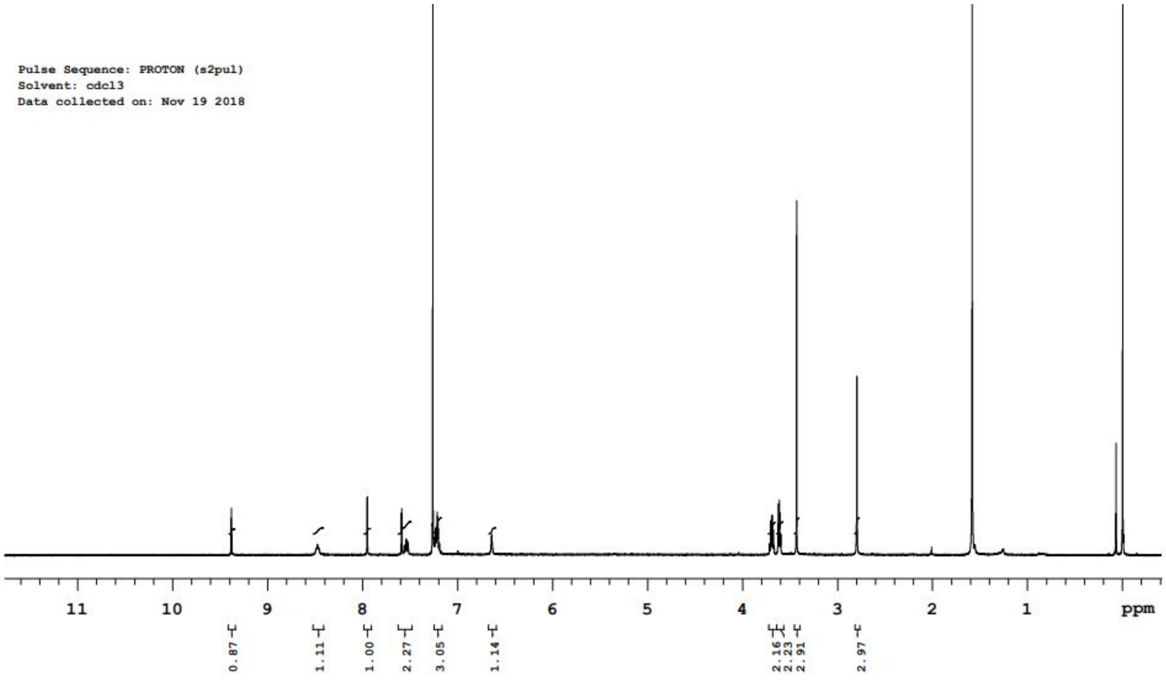

4-((2-fluorophenyl)amino)-6-methyl-N-(tetrahydro-2H-pyran-4-yl)-1,7-naphthyridine-2-carboxamide (compound **19**)

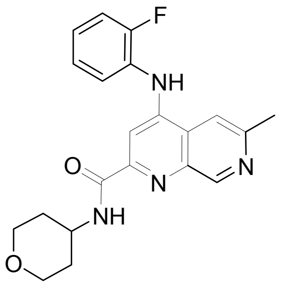

The title compound was prepared according to the procedure of Example 3, using 2-fluoroaniline as the starting material.

Peptide coupling step: To a solution of 4-((2-fluorophenyl)amino)-6-methyl-1,7-naphthyridine-2-carboxylic acid (85 mg, 0.29 mmol) in dichloromethane (1.4 mL) was added pivaloyl chloride (53 µl, 0.43 mmol) and triethylamine (120 µl, 0.86 mmol). The mixture was stirred at room temperature for 5 min. Then, tetrahydro-2*H*-pyran-4-amine (36 µl, 0.34 mmol) was added. The reaction was further stirred at room temperature for 5 min. The reaction was then diluted with dichloromethane and water. The phases were separated and the aqueous layer was further extracted with dichloromethane. The combined organic layers were dried over magnesium sulfate, filtered and concentrated. The resulting crude oil was purified by column chromatography (0-100% ethyl acetate in heptanes) and lyophilized to afford 4-((2-fluorophenyl)amino)-6-methyl-*N*-(tetrahydro-2H-pyran-4-yl)-1,7-naphthyridine-2-carboxamide (76 mg, 0.20 mmol, 69 % yield) as a white solid.

LCMS (ESI): *m/z* = 381.4 $[M+H]^+$; ^1H NMR (500 MHz, $CDCl_3$) δ 9.41 (s, 1H), 8.17 (d, *J* = 8.3 Hz, 1H), 7.97 (s, 1H), 7.62 (s, 1H), 7.56 (t, *J* = 7.5 Hz, 1H), 7.27 – 7.20 (m, 3H), 6.67 (s, 1H), 4.26 – 4.14 (m, 1H), 4.06 (d, *J* = 12.3 Hz, 2H), 3.59 (t, *J* = 11.5 Hz, 2H), 2.83 (s, 3H), 2.05 (d, *J* = 12.9 Hz, 2H), 1.75 (qd, *J* = 11.6, 4.5 Hz, 2H).

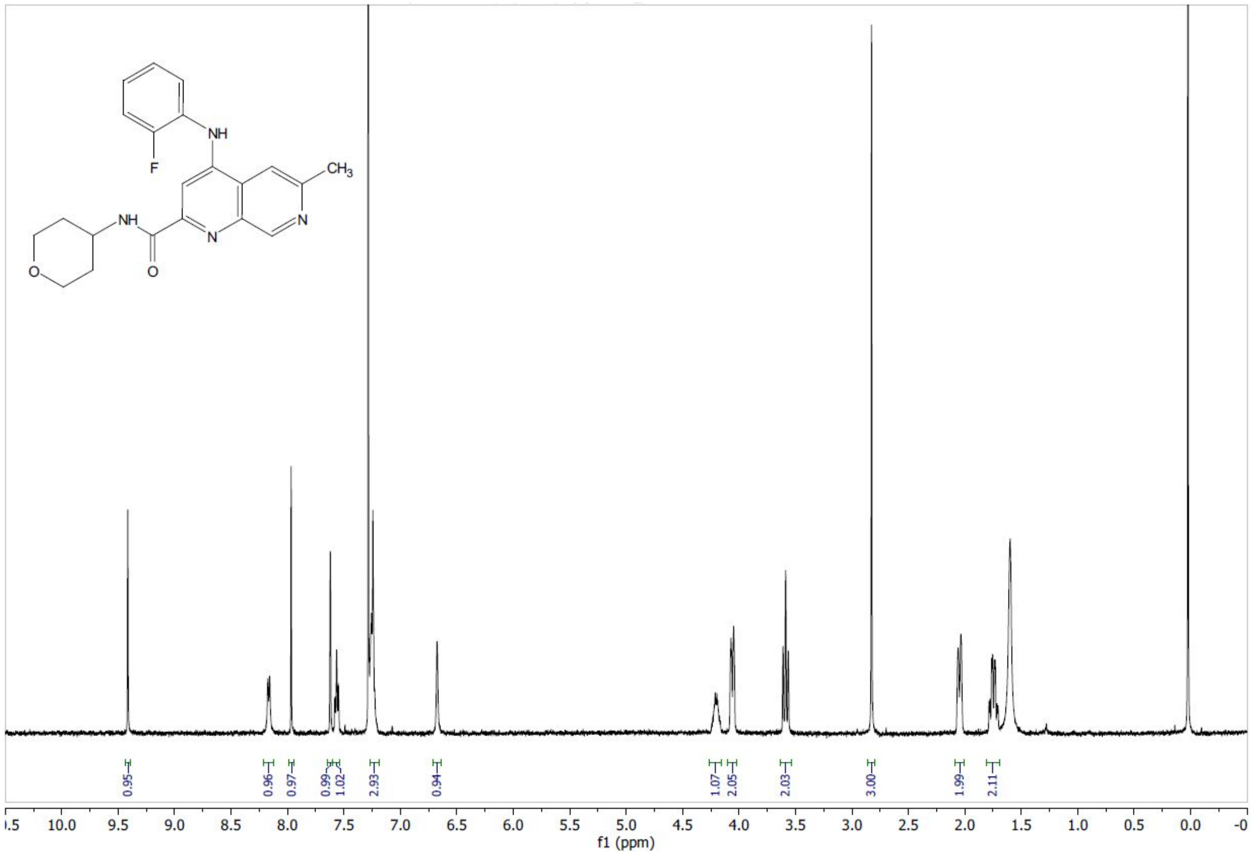

Example 5: N-cyclopropyl-6-methyl-4-(phenylamino)-1,7-naphthyridine-2-carboxamide (compound **21**)

Scheme S6. Synthesis of compound **21**.

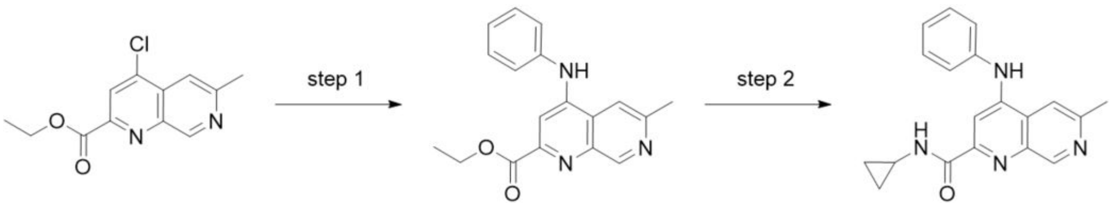

<u>Step 1:</u> **Ethyl 6-methyl-4-(phenylamino)-1,7-naphthyridine-2-carboxylate**

Ethyl 4-chloro-6-methyl-1,7-naphthyridine-2-carboxylate (80 mg, 0.32 mmol) and p-toluenesulfonic acid monohydrate (1.2 mg, 6.4 μmol) were dissolved in methanol (1.0 mL). Aniline (29 μL, 0.32 mmol) was added and the mixture was stirred in a microwave at 125 °C for 20 min. The reaction was concentrated to dryness.

<u>Step 2:</u> **N-cyclopropyl-6-methyl-4-(phenylamino)-1,7-naphthyridine-2-carboxamide**

To ethyl 6-methyl-4-(phenylamino)-1,7-naphthyridine-2-carboxylate (33 mg, 0.11 mmol) in 1,4-dioxane (250 μL) was added cyclopropylamine (11 μL, 0.16 mmol) followed by a 1.0 M solution of lithium bis(trimethylsilyl)amide in THF (322 μL, 0.32 mmol). The reaction was stirred for 15 min at room temperature. The crude mixture was diluted with methanol and filtered through a 0.45 μm polytetrafluoroethylene syringe-tip filter. The product was purified by reverse-phase HPLC (0.1%

trifluoroacetic acid water and acetonitrile) to afford *N*-cyclopropyl-6-methyl-4-(phenylamino)-1,7-naphthyridine-2-carboxamide (10.7 mg, 0.024 mmol, 23 % yield) as a bright yellow solid.

LCMS (ESI): *m*/*z* = 319.3 [M+H]$^+$; ^{1}H NMR (500 MHz, DMSO) δ 9.43 (s, 1H), 9.27 (s, 1H), 8.77 (d, *J* = 4.8 Hz, 1H), 8.25 (s, 1H), 7.63 (s, 1H), 7.56 – 7.48 (m, 2H), 7.45 – 7.39 (m, 2H), 7.32 – 7.25 (m, 1H), 2.93 – 2.84 (m, 1H), 2.71 (s, 3H), 0.77 – 0.66 (m, 4H).

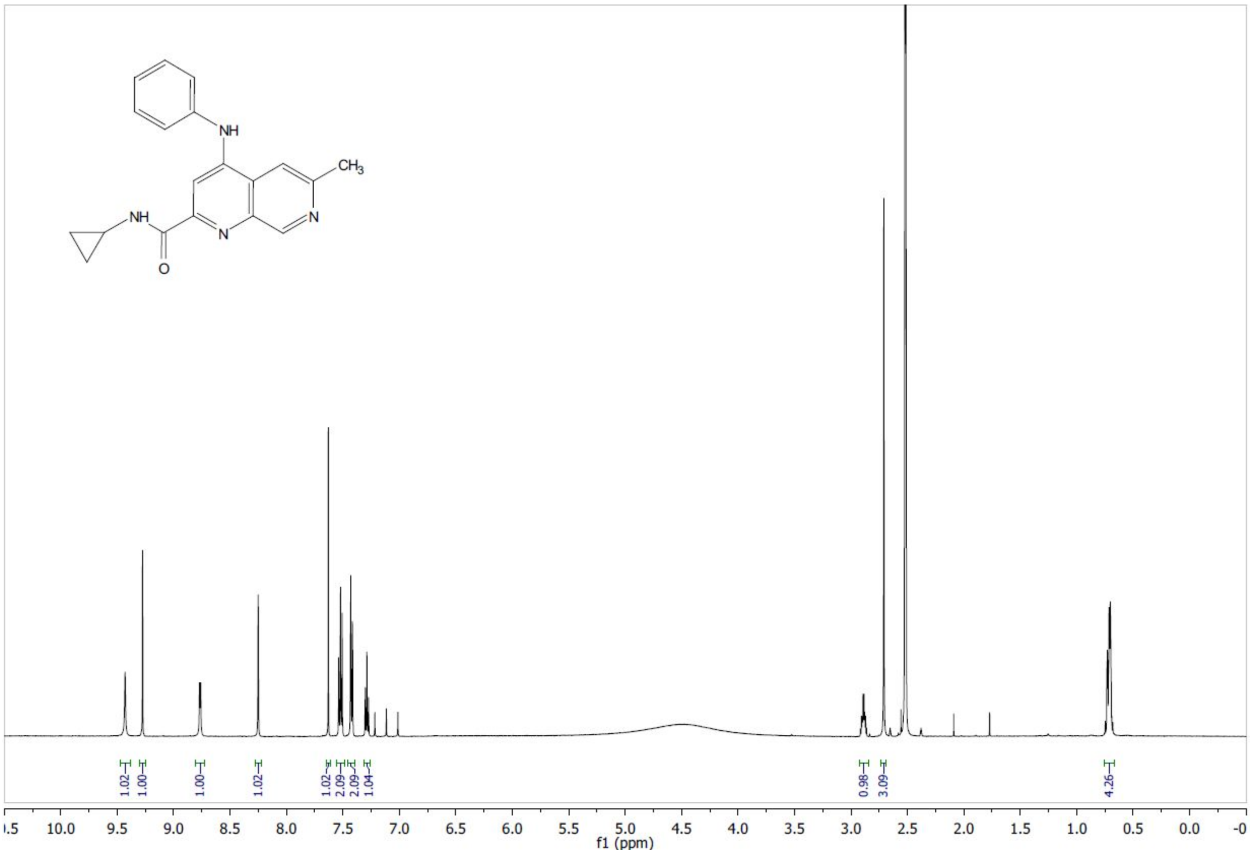

Example 6: (*E*)-4-((2-fluorophenyl)amino)-*N*-(2-methoxyethyl)-5-(prop-1-en-1-yl)picolinamide (compound **13**)

Scheme S7. Synthesis of compound **13**.

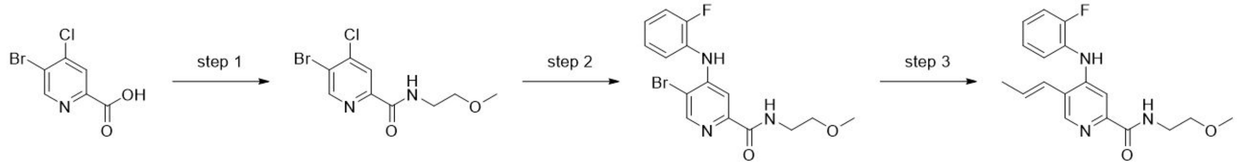

Step 1: **5-bromo-4-chloro-*N*-(2-methoxyethyl)picolinamide**

A mixture of 5-bromo-4-chloropicolinic acid (400 mg, 1.7 mmol), 2-(3*H*-[1,2,3]triazolo[4,5-*b*]pyridin-3-yl)-1,1,3,3-tetramethylisouronium hexafluorophosphate(V) (971 mg, 2.6 mmol) and 2-methoxyethan-1-amine (153 mg, 2.0 mmol) was dissolved in dimethylformamide (15 mL). *N*-ethyl-*N*-isopropylpropan-2-amine (888 µL, 5.1 mmol) was added and the reaction was stirred at room temperature for 3 hours. The mixture was diluted with ethyl acetate and washed with water and brine. The organic layer was dried over sodium sulfate

and concentrated to dryness to afford 5-bromo-4-chloro-*N*-(2-methoxyethyl)picolinamide (350 mg, 1.2 mmol, 70 % yield) as a brown solid.

Step 2: **5-bromo-4-((2-fluorophenyl)amino)-*N*-(2-methoxyethyl)picolinamide**

A solution of 5-bromo-4-chloro-*N*-(2-methoxyethyl)picolinamide (200 mg, 0.68 mmol), 2-fluoroaniline (152 mg, 1.4 mmol) and 4-methylbenzenesulfonic acid monohydrate (3.9 mg, 0.020 mmol) in methanol (0.80 mL) was heated in a microwave at 150 ^{o}C for 3 hours. The volatiles were removed under vacuum. The residue was taken up in ethyl acetate and washed with water and brine. The organic layer was dried over sodium sulfate and concentrated to dryness. The resulting crude material was purified by column chromatography (0-40% ethyl acetate in hexane) to afford 5-bromo-4-((2-fluorophenyl)amino)-*N*-(2-methoxyethyl)picolinamide (60 mg, 0.18 mmol, 47 % yield) as a pale yellow gum.

Step 3: **(*E*)-4-((2-fluorophenyl)amino)-*N*-(2-methoxyethyl)-5-(prop-1-en-1-yl)picolinamide**

A solution of 5-bromo-4-((2-fluorophenyl)amino)-*N*-(2-methoxyethyl)picolinamide (150 mg, 0.41 mmol), (*E*)-prop-1-en-1-ylboronic acid (53 mg, 0.61 mmol) and potassium carbonate (173 mg, 0.82 mmol) in 1,4-dioxane (5.0 mL) and water (2.0 mL) was sparged with argon for 10 minutes. Then, [1,1′-bis(diphenylphosphino)ferrocene]dichloropalladium(II)-dichloromethane (50 mg, 0.061 mmol) was added. The resulting mixture was heated at 95 ^{o}C for 3 hours. The reaction was diluted with ethyl acetate and washed with water and brine. The organic layer was dried over anhydrous sodium sulfate and concentrated. The resulting crude oil was purified by column chromatography (0-40% ethyl acetate in hexane) to afford (*E*)-4-((2-fluorophenyl)amino)-*N*-(2-methoxyethyl)-5-(prop-1-en-1-yl)picolinamide (60 mg, 0.18 mmol, 47 % yield) as a pale yellow gum.

LCMS (ESI): *m*/*z* = 330.4 $[M+H]^+$; ^{1}H NMR (400 MHz, DMSO) δ 8.55 – 8.48 (m, 1H), 8.38 (s, 1H), 8.24 (s, 1H), 7.37 – 7.27 (m, 4H), 7.05 (d, J = 3.1 Hz, 1H), 6.74 (dd, J = 15.7, 1.4 Hz, 1H), 6.40 – 6.30 (m, 1H), 3.43 – 3.38 (m, 4H), 3.25 (s, 3H), 1.94 (dd, J = 6.6, 1.7 Hz, 3H).

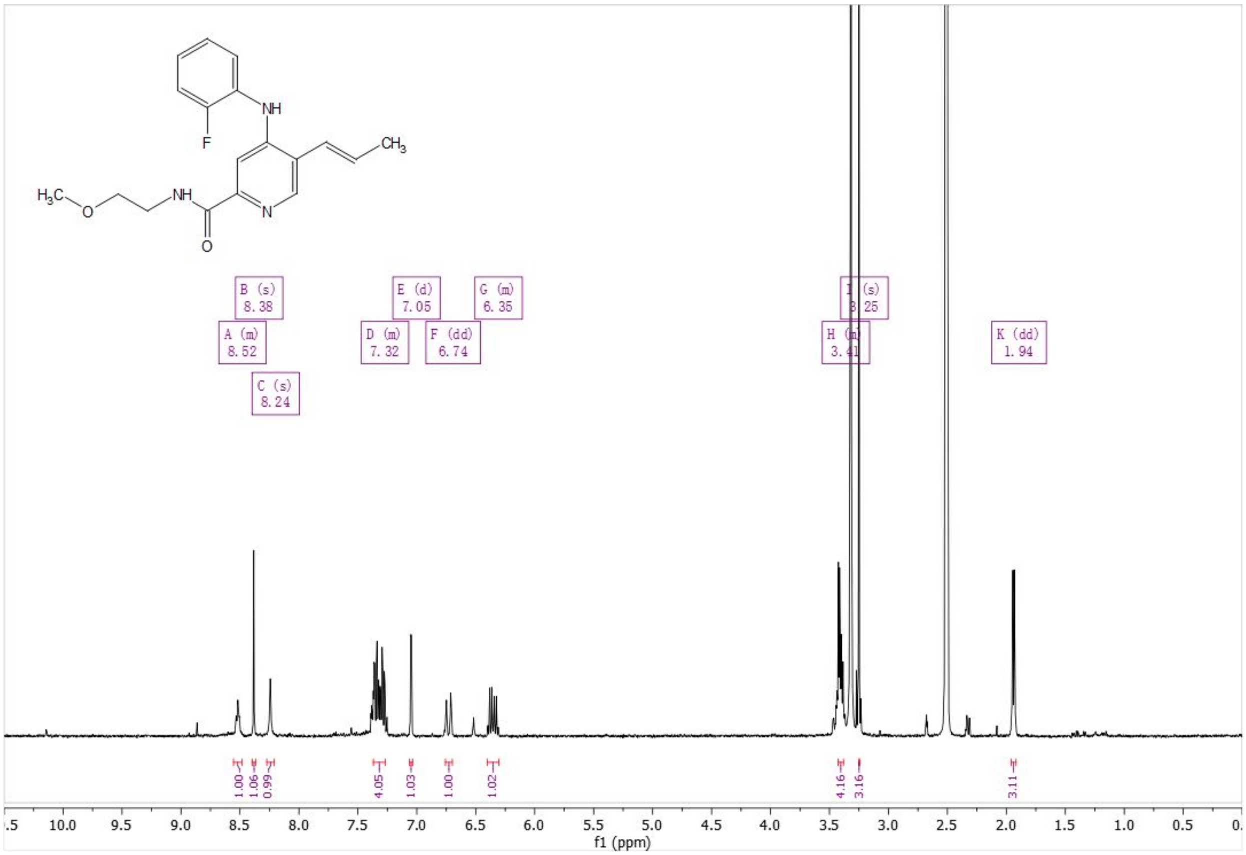

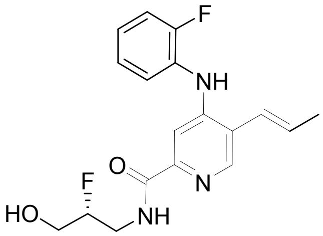

Example 7: (*R,E*)-N-(2-fluoro-3-hydroxypropyl)-4-((2-fluorophenyl)amino)-5-(prop-1-en-1-yl)picolinamide (compound **14**)

Scheme S8. Synthesis of compound **14.**

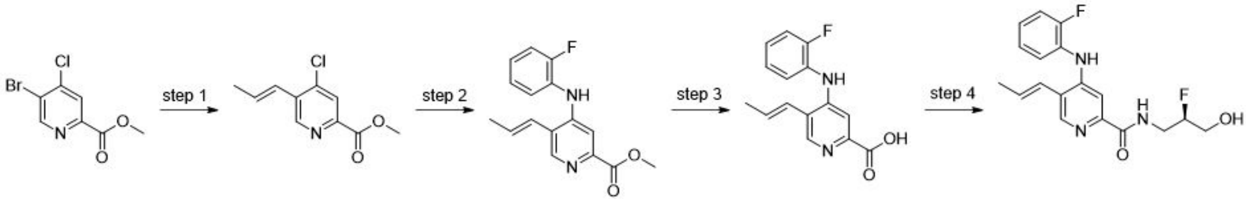

Step 1: **methyl (*E*)-4-chloro-5-(prop-1-en-1-yl)picolinate**

A solution of methyl 5-bromo-4-chloropicolinate (2.2 g, 8.8 mmol), potassium (*E*)-trifluoro(prop-1-en-1-yl)borate (1.6 g, 11 mmol) and triethylamine (2.5 mL, 18 mmol) in 1,4-dioxane (20 mL) was sparged with argon for 10 minutes. Then, [1,1′-bis(diphenylphosphino)ferrocene]dichloropalladium(II)-dichloromethane (1.0 g, 1.3 mmol) was added. The resulting mixture was heated at 95 ^{o}C for 1 hour. The reaction was diluted with ethyl acetate and washed with water and brine. The organic layer was dried over anhydrous sodium sulfate and concentrated. The resulting crude oil was purified by column chromatography (0-30% ethyl

acetate in hexane) to afford methyl (*E*)-4-chloro-5-(prop-1-en-1-yl)picolinate (1.3 g, 6.2 mmol, 70% yield) as an offwhite solid.

Step 2: **methyl (*E*)-4-((2-fluorophenyl)amino)-5-(prop-1-en-1-yl)picolinate**

A solution of methyl (*E*)-4-chloro-5-(prop-1-en-1-yl)picolinate (500 mg, 2.4 mmol), 2-fluoroaniline (394 mg, 3.6 mmol) and cesium carbonate (1.5 g, 4.7 mmol) in toluene (25 mL) was sparged with argon for 10 minutes. Then, tris(dibenzylideneacetone)dipalladium(0) (108 mg, 0.12 mmol) and 2-(dicyclohexylphosphino)-3,6-dimethoxy-2′,4′,6′-triisopropyl-1,1′-biphenyl (190 mg, 0.36 mmol) were added. The resulting mixture was heated at 100 ^{o}C for 1 hour. The reaction was diluted with ethyl acetate and washed with water and brine. The organic layer was dried over anhydrous sodium sulfate and concentrated. The resulting crude oil was purified by column chromatography (0-30% ethyl acetate in hexane) to afford (*E*)-4-((2-fluorophenyl)amino)-5-(prop-1-en-1-yl)picolinate (450 mg, 1.6 mmol, 66% yield) as an offwhite solid.

Step 3: **(*E*)-4-((2-fluorophenyl)amino)-5-(prop-1-en-1-yl)picolinic acid**

A solution of (*E*)-4-((2-fluorophenyl)amino)-5-(prop-1-en-1-yl)picolinate (450 mg, 1.6 mmol) and lithium hydroxide monohydrate (99 mg, 2.4 mmol) in methanol (15 mL) and water (10 mL) was stirred at room temperature for 2 hours. The volatiles were removed under vacuum and the pH of the resulting solution was acidified with an aqueous saturated solution of potassium hydrogen sulfate, which caused precipitation. The solids were recovered by filtration and further dried under vacuum to afford (*E*)-4-((2-fluorophenyl)amino)-5-(prop-1-en-1-yl)picolinic acid as an offwhite solid (400 mg, 1.5 mmol, 94% yield).

Step 4: **(*R,E*)-N-(2-fluoro-3-hydroxypropyl)-4-((2-fluorophenyl)amino)-5-(prop-1-en-1-yl)picolinamide**

To a solution of (*E*)-4-((2-fluorophenyl)amino)-5-(prop-1-en-1-yl)picolinic acid (50 mg, 0.18 mmol) and (*R*)-3-amino-2-fluoropropan-1-ol (26 mg, 0.28 mmol) in dimethylformamide (800 µL) was added triethylamine (77 µL, 0.55 mmol) and a 50% weight solution of 2,4,6-tripropyl-1,3,5,2,4,6-trioxatriphosphinane 2,4,6-trioxide in dimethylformamide (214 µL, 0.37 mmol). The reaction was stirred at room temperature for 3 hours. The mixture was filtered through a 0.45 µm polytetrafluoroethylene syringe-tip filter. The product was purified by reverse-phase HPLC (0.1% formic acid water and acetonitrile) to afford *(R,E)-N*-(2-fluoro-3-hydroxypropyl)-4-((2-fluorophenyl)amino)-5-(prop-1-en-1-yl)picolinamide (29 mg, 0.082 mmol, 45% yield) as a white solid.

LCMS (ESI): m/z = 348.3 $[M+H]^+$; ^{1}H NMR (500 MHz, DMSO) δ 8.74 (t, *J* = 6.2 Hz, 1H), 8.40 (s, 1H), 8.27 (s, 1H), 7.40 – 7.26 (m, 4H), 7.06 (d, *J* = 3.0 Hz, 1H), 6.75 (d, *J* = 15.7 Hz, 1H), 6.37 (dq, *J* = 15.7, 6.6 Hz, 1H), 4.70 – 4.55 (m, 1H), 3.63 – 3.41 (m, 5H), 1.95 (dd, *J* = 6.6, 1.8 Hz, 3H).

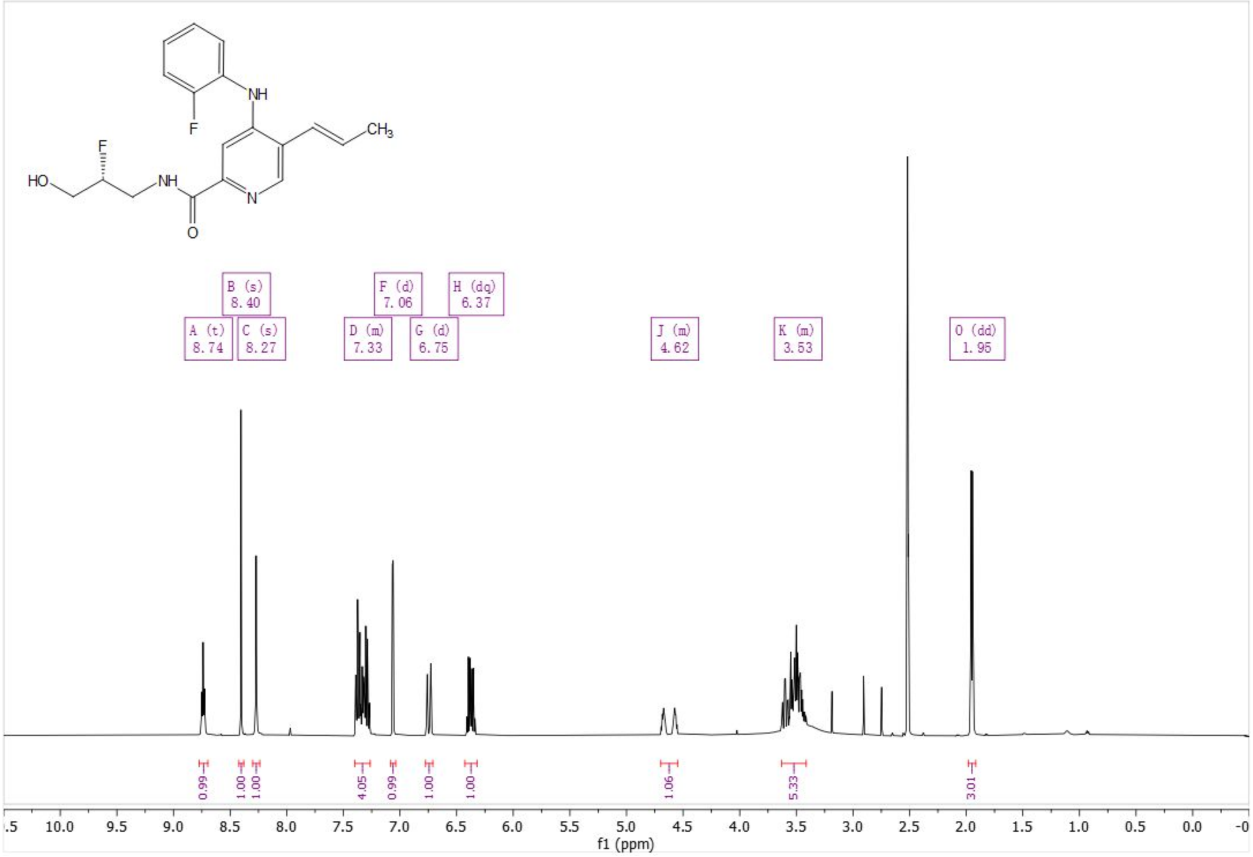

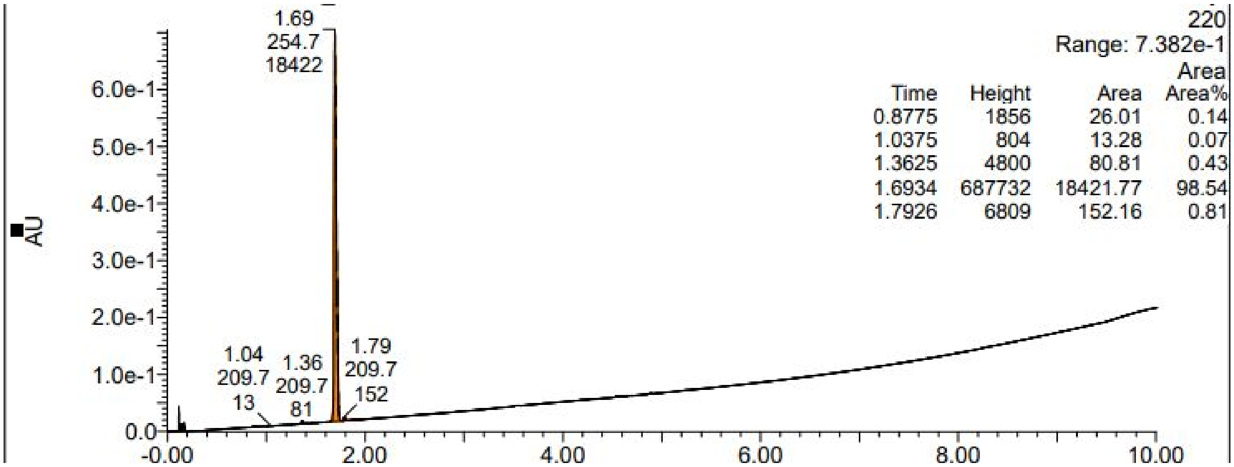

(R)-4-((2,3-difluorophenyl)amino)-5-ethoxy-N-(2-fluoro-3-hydroxypropyl)picolinamide (compound **15**)

Scheme S9. Synthesis of compound **15**.

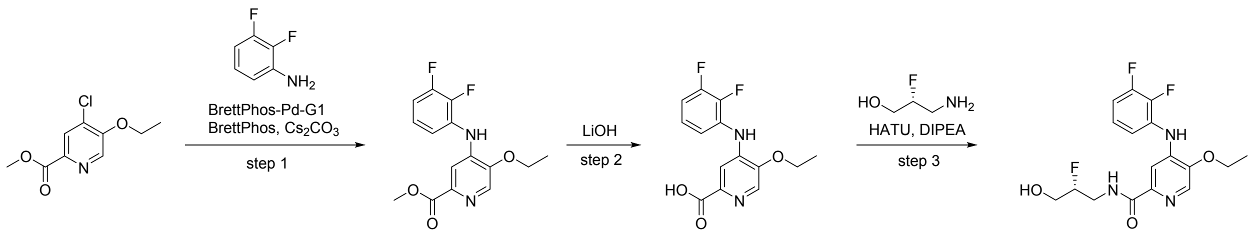

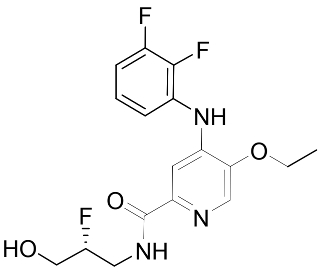

Step 1: To a dry MW vial under N2 was added methyl 4-chloro-5-ethoxypicolinate (100 mg, 0.464 mmol), Pd-BrettPhos-G1 (37 mg, 0.046 mmol), BrettPhos (25 mg, 0.0046 mmol), and Cs2CO3 (378 mg, 1.16 mmol). Dioxane (4.6 mL) was added to the flask and the flask was evacuated/refilled with N2 3x. 2,3-difluoroaniline (94 uL, 120 mg, 0.93 mmol) was introduced and the flask was sealed and stirred in a MW at 100 °C for 2 h. LCMS indicated full conversion. The mixture was diluted with EtOAc and filtered through a pad of SiO2. The filtrate was concentrated and purified on SiO2 column on the combiflash with a gradient of 50-100% EtOAc in hept to isolate methyl 4-((2,3-difluorophenyl)amino)-5-ethoxypicolinate as white solids (82 mg, 57%). LCMS (ESI): m/z = 309.2 $[M+H]^+$

Step 2: ethyl 4-((2,3-difluorophenyl)amino)-5-ethoxypicolinate (80 mg, 0.248 mmol) and LiOH.H2O (52 mg, 1.24 mmol) were suspended in MeOH (2 mL) and stirred at 50 °C for 2 h. LCMS indicated full conversion. The mixture was quenched with formic acid and concentrated in vacuo. The solid mass as then suspended in water, filtered, collected, and dried to afford 4-((2,3-difluorophenyl)amino)-5-ethoxypicolinic acid as a beige solid (72 mg, 99%). LCMS (ESI): m/z = 295.1 $[M+H]^+$

Step 3: To a suspension of 4-((2,3-difluorophenyl)amino)-5-ethoxypicolinic acid (29.4 mg, 0.10 mmol) and (R)-3-amino-2-fluoropropan-1-ol (13.97 mg, 0.150 mmol) in DMF (Volume: 0.5 mL) was added DIPEA (0.052 mL, 0.300 mmol) and HATU (45.6 mg, 0.120 mmol). The mixture was stirred at RT for 1 h. LCMS indicated full conversion. The mixture was diluted with DCM, separated with water, washed with water and brine, dried over Na2SO4, filtered, and concentrated. The was purified by reverse-phase HPLC (0.1% trifluoroacetic acid water and acetonitrile), lyophilized, and freebased with EtOAc/NaHCO3 to afford the desired product (18.6 mg, 0.050 mmol, 49.9 % yield) as a yellow powder. LCMS (ESI): m/z = 370.2 $[M+H]^+$; ^1H NMR (500 MHz, DMSO-*d6*) δ 8.67 (s, 1H), 8.15 – 8.03 (m, 1H), 7.38 – 7.07 (m, 4H), 4.72 – 4.50 (m, 1H), 4.28 (q, J = 7.0 Hz, 2H), 3.63 – 3.42 (m, 4H), 1.42 (t, J = 6.9 Hz, 3H).

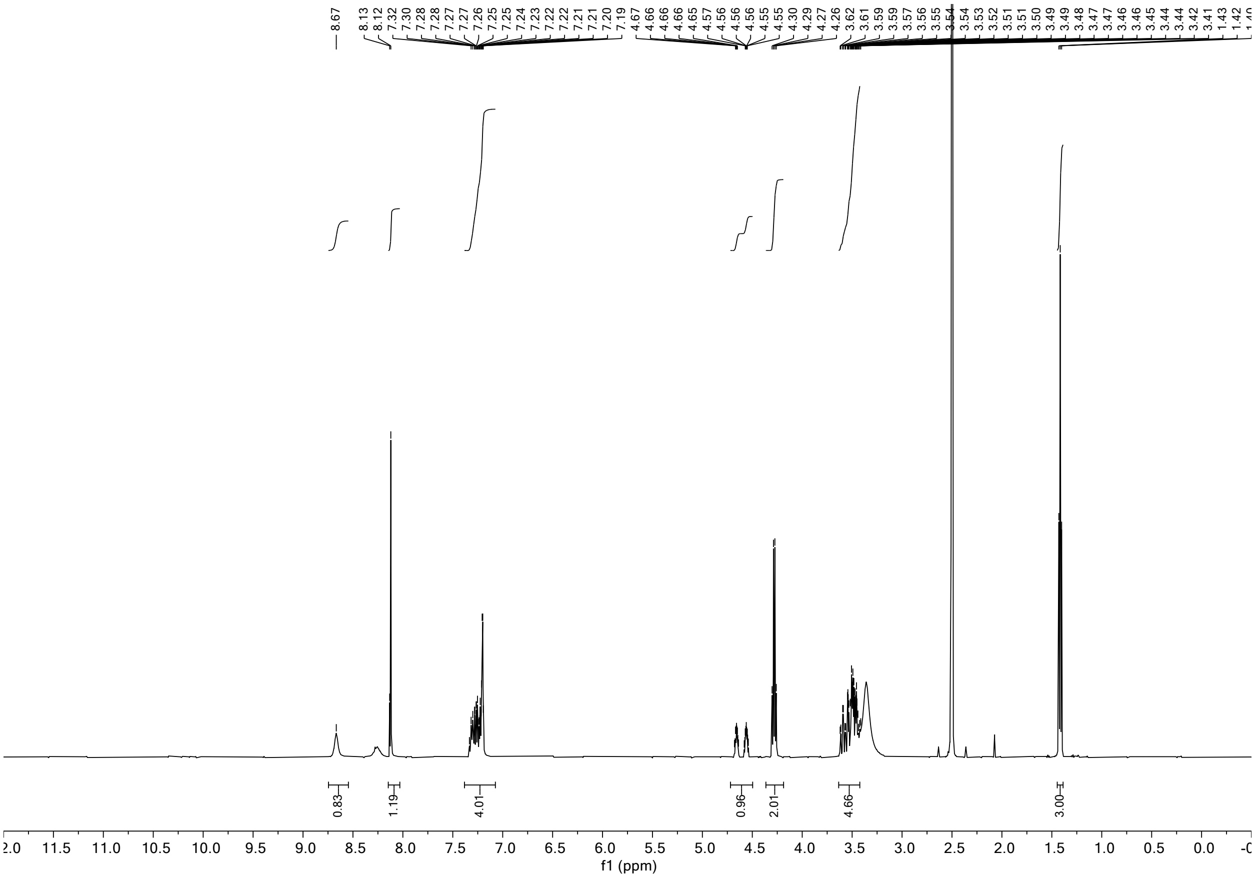

2) Potency and clearance of additional compounds

Table S1. Structure and in vitro data for 1,5- and 1,8-naphthyridines.

Compound	Structure	T. b. brucei EC_{50} [µM]	LM CL_{int} R/H [µL/min/mg]	SMILES
SI1	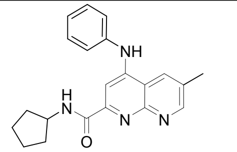	10.78	55 / <25	Cc3cnc2nc(cc(Nc1ccccc1)c2c3)C(=O)N4CCCC4
SI2	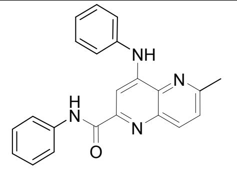	12.41	60 / 61	Cc3ccc2nc(cc(Nc1ccccc1)c2n3)C(=O)Nc4ccccc4

3) Predicted and measured K_p and $K_{p,uu}$

Table S2. Predicted and measured K_p. PPB and BTB and calculated $K_{pu,u}$.

Cmp	LogBBB (predicted)	K_p (Measured, 5 min, 60 min)	PPB mouse	BTB rat	$K_{pu,u}$ (5 min, 60 min)
4	0.11	1.2, 0.61	98.0	98.5	0.90, 0.45
7	0.36	0.61, 0.42	99.0	99.1	0.55, 0.38
5	0.23	1.2, 1.5	99.2	99.4	0.90, 1.1
6	0.46	1.2, 0.68	98.8	99.3	0.70, 0.40
16	0.08	0.71, 0.56	97.7	95.7	1.3, 1.0
9	0.21	0.62, 0.38	92.7	95.7	0.37, 0.22
14	0.09	1.1, 0.99	92.4	95.9	0.59, 0.53
17	-0.12	0.34, 0	89.7	91.3	0.28, ND
12	-0.05	0.50, 0.20	95.5	96.0	0.44, 0.18
18	0.06	0.88, 0	91.3	91.2	0.89, ND
3	-0.27	0.17, 0.26	97.0	96.0	0.22, 0.34
19	0.03	1.0, 1.1	88.2	94.4	0.47, 0.52
20	0.05	0.85, 0	98.2	98.5	0.71, ND
21	0.00	0.58, 0	95.1	95.4	0.54, ND
8	0.10	0.93, 0.89	94.5	97.0	0.51, 0.49
13	0.23	1.4, 0	96.1	97.0	1.1, ND
